# A quantitative *Streptococcus pyogenes*–human protein–protein interaction map reveals localization of opsonizing antibodies

**DOI:** 10.1038/s41467-019-10583-5

**Published:** 2019-06-21

**Authors:** Lotta Happonen, Simon Hauri, Gabriel Svensson Birkedal, Christofer Karlsson, Therese de Neergaard, Hamed Khakzad, Pontus Nordenfelt, Mats Wikström, Magdalena Wisniewska, Lars Björck, Lars Malmström, Johan Malmström

**Affiliations:** 1 Lund University, Faculty of Medicine, Department of Clinical Sciences, Division of Infection Medicine, Lund University, SE-22184 Lund, Sweden; 2 S3IT, University of Zurich, CH-8057 Zurich, Switzerland; 3 Institute of Computational Science, University of Zurich, CH-8057 Zurich, Switzerland; 4 Swiss Institute of Bioinformatics, Lausanne, CH-1015 Switzerland; 5 Novo Nordisk Foundation Center for Protein Research, University of Copenhagen, DK-2200 Copenhagen, Denmark; 6 Present Address: Cantargia AB, SE-22381 Lund, Sweden; 7 Present Address: Amgen Inc., Attributes Sciences, One Amgen Center Drive, Thousand Oaks, CA 91320 USA

**Keywords:** Proteomics, Bacterial host response, Pathogens, Protein-protein interaction networks

## Abstract

A fundamental challenge in medical microbiology is to characterize the dynamic protein–protein interaction networks formed at the host–pathogen interface. Here, we generate a quantitative interaction map between the significant human pathogen, *Streptococcus pyogenes*, and proteins from human saliva and plasma obtained via complementary affinity-purification and bacterial-surface centered enrichment strategies and quantitative mass spectrometry. Perturbation of the network using immunoglobulin protease cleavage, mixtures of different concentrations of saliva and plasma, and different *S. pyogenes* serotypes and their isogenic mutants, reveals how changing microenvironments alter the interconnectivity of the interaction map. The importance of host immunoglobulins for the interaction with human complement proteins is demonstrated and potential protective epitopes of importance for phagocytosis of *S. pyogenes* cells are localized. The interaction map confirms several previously described protein–protein interactions; however, it also reveals a multitude of additional interactions, with possible implications for host–pathogen interactions involving other bacterial species.

## Abstract (web summary)

Characterizing host-pathogen protein interactions can help elucidate the molecular basis of bacterial infections. Here, the authors use an integrative proteomics approach to generate a quantitative map of protein interactions between *Streptococcus pyogenes* and human saliva and plasma.

## Introduction

Many significant bacterial pathogens produce proteins, that form interactions with human host proteins to evade the immune system^1^, acquire metabolites^2^, and facilitate adherence^3^. At the same time, protein components from the adaptive and innate immune system of the host, such as immunoglobulins and proteins of the complement system, interact with bacterial surfaces and effector proteins to promote bacterial clearance. This multitude of protein interactions can result in the formation of complex protein–protein interaction networks via inter-species and intra-species protein connections, a reflection of the evolutionary interplay between host and pathogen^3^. A quantitative characterization of these host–pathogen protein interaction networks is central to understanding the molecular basis of bacterial infections.

Mass spectrometry (MS) has evolved as a key technology in the large-scale characterization of protein interactions^4^. As recently reviewed, affinity purification–MS (AP–MS), cross-linking MS, and proximity-dependent labeling–MS have been used to characterize host–pathogen protein interactions^5^. However, these efforts have typically focused on characterizing static protein interaction networks between human host proteins and microbial species. There is increasing awareness that quantification of protein interaction networks will provide a more dynamic understanding of these interactions^6^. The recent development of data independent analysis (DIA)-MS^7^ has provided new opportunities to consistently quantify protein–protein interactions in different states^6^. In DIA-MS, proteome maps are generated based on data-independent acquisition, followed by protein quantification using previously established assay libraries. Importantly, DIA-MS can provide accurate protein quantification with a high degree of data completeness and dynamic range without specifying target peptides prior to data acquisition^6^.

*Streptococcus pyogenes* is an important human pathogen, capable of forming a dense and protein-rich inter-species interaction network outside its cell wall^2,8,9^. This Gram-positive bacterium has diverse clinical manifestations, ranging from mild and common local infections, such as tonsillitis, impetigo, and erysipelas to life-threating systemic diseases like sepsis, meningitis, and necrotizing fasciitis^10^. The incidence of tonsillitis caused by *S. pyogenes* is 1000-fold higher than invasive *S. pyogenes* infections^11^; even so, *S. pyogenes* is responsible for over 160,000 deaths every year^12^. Infection of the upper respiratory tract by *S. pyogenes* is characterized by vascular leakage, activation of innate and adaptive immunity, and by infiltration of inflammatory cells^13^, resulting in a dramatic increase in protein mass at the infection site^14^. Many of the most abundant plasma proteins infiltrating the site of infection, such as fibrinogen, albumin, and immunoglobulins, have previously been shown to form protein interactions with the M1 protein^8,9,15^. M proteins form a fibrillar layer on the surface of *S. pyogenes*, and depending on serotype, harbor a variety of repeat regions that are used to bind human proteins, including fibrinogen, fibronectin, albumin, plasminogen, proteins of the complement system, and immunoglobulins (IgA, IgG1–4)^16^, and can mediate interactions with additional human proteins creating large inter-species protein complexes^17^. The binding can be environment specific, as IgGs can bind to some M proteins via their antigen-binding fragments (Fab) under antibody-rich conditions, such as plasma, or via their Fc fragment in an antibody-poor scenario, such as saliva^18^. *S. pyogenes* encodes a wide variety of other virulence factors, such as adhesins and exotoxins, which are used in host cell adherence, internalization, and invasion during infection^10^, though many of these are poorly characterized. *S. pyogenes* also produces a handful of specific enzymes, such as the immunoglobulin specific protease IdeS^19^, the glycosidases EndoS and EndoS2^20,21^ and the cysteine protease SpeB^22^. These and other secreted proteins can distort the interactions with human proteins; thereby, result in a highly dynamic interaction network at the *S. pyogenes* cell surface^8^.

In this study, we generate a quantitative *S. pyogenes*–human plasma and saliva interaction map to determine dynamic protein interactions at the host–pathogen interface. In contrast to previous studies^8,9^, we use here DIA-MS and a combined affinity-purification and bacterial-surface-centered host protein enrichment strategies to determine how the human–pathogen interaction networks are regulated. Collectively, the interaction map reveals how changing microenvironments alter the interconnectivity of protein networks based on the formation of both inter-species and intra-species protein interactions, which facilitates the detection of protective epitopes important for *S. pyogenes* interaction and internalization during phagocytosis.

## Results

### The streptococcal–human protein interaction network

**Fig. 1:**
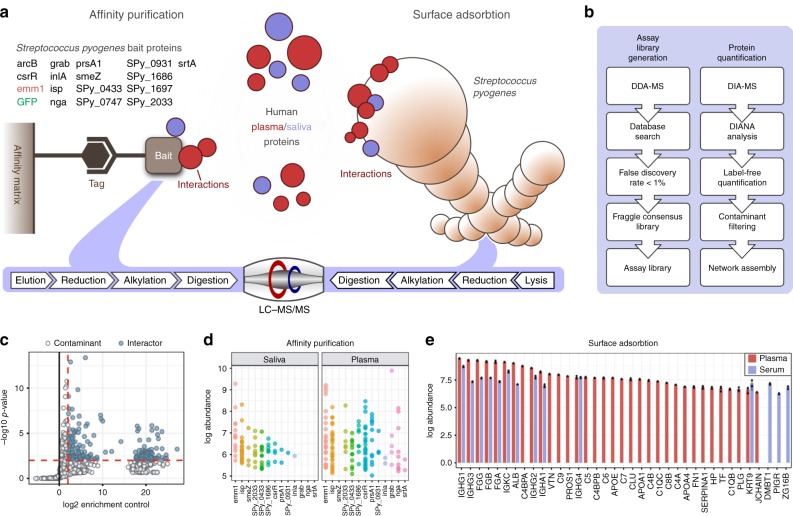
Construction of the combined AP–DIA and SA–DIA interaction maps. **a** The affinity tagged streptococcal bait proteins used in the AP–DIA approach are indicated in black with their respective gene names, the M1 protein (*emm1*) is highlighted in red, and sfGFP used as a negative control is shown in green. Eight of the proteins included in this study are uncharacterized (Supplementary Data 1); albeit some of them have homologs in other species. Additionally, we included seven characterized or partly characterized proteins (Supplementary Data 1). Throughout this paper all proteins are referred to by their gene names, as the majority of the streptococcal proteins included in this study are uncharacterized proteins without defined protein names. All proteins were expressed as recombinant proteins, attached to an affinity-matrix via an affinity-tag, and interacting proteins from plasma and saliva were captured, prior to sample elution and reduction, alkylation and trypsin-digestion for mass spectrometry (MS) analysis. In the SA–DIA approach, intact bacteria were used as an affinity platform to capture human plasma and saliva proteins, prior to cell lysis and reduction, alkylation, and trypsin-digestion. **b** The samples from the AP–DIA and SA–DIA approaches were analyzed via data-dependent acquisition-MS (DDA-MS) at a false discovery rate (FDR) of <1%. Fraggle^60^ was used to construct an assay library used for label-free quantification of identified peptides via DIA-MS using the DIANA software^61^, with subsequent contaminant filtering of AP–DIA data and network assembly. **c** These AP–DIA interactions were strongly filtered to include only high-confidence interactions (blue dots) that were identified with three proteotypic peptides, which were >4-fold enriched and had an adjusted *P-*value < 0.01 compared to proteins enriched using sfGFP using the Student’s *t*-test^6^ in *n* = 3 biologically independent samples. White dots indicate contaminating proteins falling below the set thresholds. **d** Intensity distribution of the 226 interacting proteins per bait in the AP–DIA approach. Each dot represents the intensity value for an interacting protein, and colors indicate the respective *S. pyogenes* bait protein. **e** Human plasma and saliva proteins identified as associated with the streptococcal surface in the SA–DIA approach. Error bars are expressed as standard deviation (s.d.) from the mean. Source data are provided as a Source Data file

To catalog the interaction network formed between *S. pyogenes* and human proteins we used a combined protein-centered and bacterial-surface centered AP strategy to isolate interacting proteins, followed by label-free quantitative MS (DIA-MS; Fig. 1a, b). In the protein-centered AP–DIA experiments 16 putative virulence factors were produced as bait proteins (Fig. 1a, Supplementary Data 1, 2). The bait proteins were selected from previous proteomic screens of *S. pyogenes* serotype M1 strains SF370, AP1, 5448, and 5448AP, MGAS5005, as well as 34 clinical strains isolated in 2012^2,8,9,23–25^. They were expressed as recombinant proteins with an affinity tag and used to capture interacting proteins from both pooled normal human plasma and saliva (Fig. 1a). Quantitative DIA-MS analysis of the captured human proteins identified 226 high-confidence specific interactions between 107 human and the 16 streptococcal bait proteins, when compared to the superfolder green fluorescent protein (sfGFP) control samples (Fig. 1c, Supplementary Fig. 1). The 226 interactions were distributed unevenly between the baits (Fig. 1d), with 81 interactions identified in saliva and 145 in plasma. Protein interaction networks incorporating the interactions between the individual baits and co-purified human proteins are given in Supplementary Fig. 2 and Supplementary Data 3. The complementary experiments using *S. pyogenes* M1 serotype strain AP1 as bait by surface adsorption (SA–DIA) resulted in the identification of 55 high-confidence human interacting proteins in either plasma or saliva, though most were identified in plasma (Fig. 1e).

**Fig. 2:**
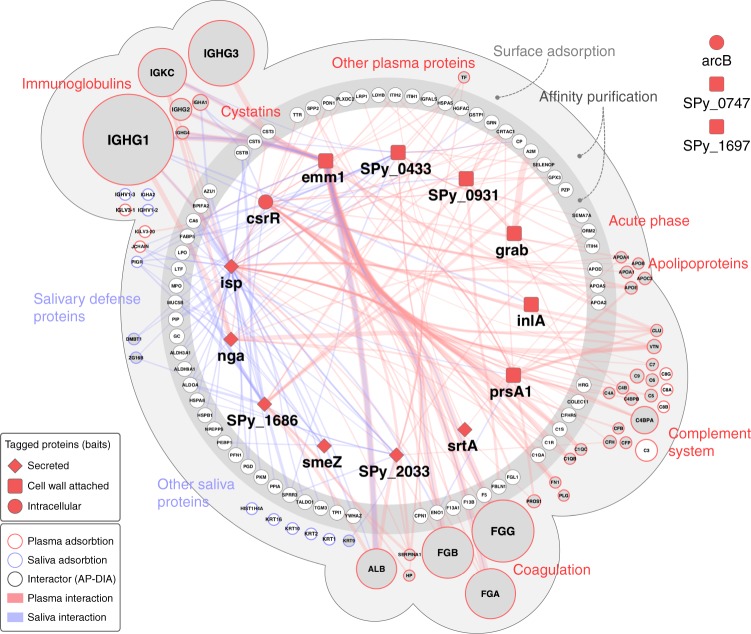
A combined network layout of the AP–DIA and SA–DIA interaction maps. Overlap of the AP–DIA and SA–DIA plasma and saliva interactomes. Streptococcal bait proteins are presented as dark red squares (cell wall-attached proteins), diamond shapes (secreted proteins) or spheres (intracellular proteins). The AP–DIA interactome is represented as the inner dark gray circle according to functional protein class, with the protein interactions indicated in red for human plasma and blue for saliva. The thickness of the edges connecting the nodes indicates the abundance of the captured human proteins in relation to the respective bait proteins. Proteins identified both in the AP–DIA and SA–DIA approaches are enclosed in the outer light gray sphere, where the circle sizes represent protein abundance derived from the SA–DIA experiments and the colors (red or blue) indicates predominant plasma or saliva protein interactions. The nodes of these overlapping interaction networks are filled in gray for easier visualization. Proteins exclusively identified in SA–DIA are placed in the outer light gray sphere and are not connected with edges. Streptococcal bait proteins without any identified human interactions are depicted outside the interactome area, being arcB, and the uncharacterized proteins Q9A0J7 (*Spy_0747*) and Q99YH8 (*Spy_1697*). The interactome views were generated using Cytoscape^69^ and modified in Adobe Illustrator. Source data are provided as a Source Data file

To visualize the overlap between the saliva and plasma interaction maps, we generated an interconnected view of the AP–DIA and SA–DIA human interacting proteins, providing captured protein abundance compared to the respective baits (Fig. 2). Of the 226 AP–DIA protein interactions identified, the 67 human proteins exclusively identified in AP–DIA are organized according to functional protein class. In total, 40 out of the 55 proteins identified by SA–DIA overlap with those identified by AP–DIA (Fig. 2). The combined AP/SA–DIA network consists of 122 human proteins subdivided into nine major functional groups, such as immunoglobulins, cystatins, acute phase proteins, apolipoproteins, and proteins involved in the coagulation and complement system (Fig. 2). The most abundant proteins adhering to the M1 streptococcal surface in plasma are coagulation (fibrinogen) proteins, complement system proteins, apolipoproteins, and immunoglobulins. In saliva, other protein groups are more abundant, such as salivary defense proteins. Comparative analysis of the interactions formed in saliva and plasma reveals a relatively small overlap of known plasma proteins, such as fibrinogen, albumin, immunoglobulins (IgG1–2 and IgG4), and ceruloplasmin (Fig. 2, Supplementary Data 3). Notably, secreted streptococcal proteins tend to interact with human saliva proteins, whereas cell wall-attached streptococcal proteins interact with human plasma proteins (Fig. 2). This suggests that *S. pyogenes* has developed distinct strategies for host defense evasion in the different ecological niches of the upper respiratory tract and plasma. M1 protein showed the highest number of protein interactions in the interaction network (Fig. 2, Supplementary Data 3), and is responsible for interactions with the most abundant human proteins at the bacterial surface, such as immunoglobulins, fibrinogen, and albumin, consistent with previous findings^8^.

Some of the interactions generated by AP–DIA and SA–DIA were assessed in deletion mutagenesis experiments, western blot analysis of the original AP reactions, reverse affinity-capture using human proteins as baits and by targeted crosslinking MS (TX-MS)^17^. These verification experiments confirms that proteins ISP and CovR bound to the complement membrane attack complex (MAC) composed of proteins C5b-C9^26^ (Fig. 2, Supplementary Fig. 2), in a manner previously described for streptococcal inhibitor of complement (SIC)^27^. Additional AP experiments in human plasma with truncated versions of ISP demonstrate that the N-terminus of ISP is responsible for MAC binding, whereas the C-terminus binds the complement system proteins C1 and clusterin (Supplementary Fig. 3). In addition, TX-MS together with western blot analysis of AP reactions demonstrate that ISP, its truncated version ISP-N and CovR interact with human fibrinogen (Supplementary Figs. 2, 3, 4; Supplementary Data 4–6). Reverse affinity-capture using tagged human serum albumin as the bait, and western blot analysis of the original AP reactions in human plasma confirms that the uncharacterized protein Q99XU1 (*SPy_2033*) interacts directly with albumin, whereas the uncharacterized protein Q99YI6 (*SPy_1686*) only does so in plasma (Supplementary Figs. 5 and 6), indicating either an indirect interaction or the need for auxiliary proteins to capture the interaction.

### Dynamic changes at the streptococcal–human interface

As a next step, the dynamics of the protein interaction networks were assessed in two separate experiments. In the first experiment, SA–DIA was used to quantitatively analyze the interaction networks formed in different mixtures of plasma and saliva to measure any inherent competition between plasma and saliva protein interactions. In the second experiment, the effect of different serotypes and their isogenic M protein (*emm*) deletion mutants on plasma protein interactions was determined.

**Fig. 3:**
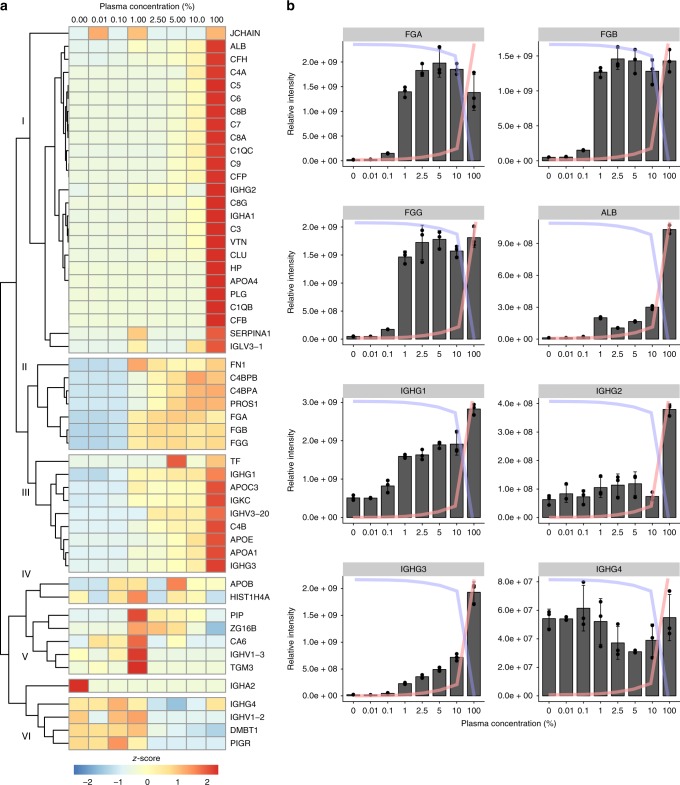
SA–DIA in a mixed plasma–saliva environment. Different mixtures of plasma in saliva (0.01%, 0.1%, 1%, 2.5%, 5%, and 10% plasma) were used to study the effect of host environment on protein–protein interactions. **a** Cluster analysis of the SA–DIA data with the M1 serotype *S. pyogenes* strain AP1 in a mixed plasma and saliva environment based on *z*-score. **b** SA–DIA interaction patterns of fibrinogen, albumin, and the different IgG subclasses in mixed plasma and saliva environments in *n* = 3 biologically independent samples. The *y*-axis represents relative intensity and *x*-axis plasma concentration (%) in panel **b**. The graph overlaid blue lines represent relative saliva concentration and the red lines relative plasma concentration in a given sample. Error bars are expressed as standard deviation (s.d.) from the mean. Source data are provided as a Source Data file

In the first experiment, SA–DIA protein interaction analysis was performed using mixtures of plasma in saliva (0.01%, 0.1%, 1%, 2.5%, 5%, and 10% plasma; Fig. 3, Supplementary Fig. 7) that reflects the range of plasma leakage in tonsil swabs from patients with tonsillitis^14,15^. Six clusters of proteins with different binding patterns were identified (Fig. 3a). Protein interactions in the most numerous clusters (clusters I and II in Fig. 3a) correlates with the increase in plasma concentration, and includes proteins that interact in a nonspecific manner or adhere to several binding sites. An example of a complex in cluster I is the complement MAC, composed of the complement proteins C5b-C9^28,29^. Other proteins in these clusters are IgGs of the 1, 2, and 3 subclasses, which recognize several different epitopes due to their polyclonal nature (Fig. 3a, b). By contrast, several of the known M1 protein binders, such as fibrinogen and fibronectin, display saturated binding profiles at 1% plasma (cluster III in Fig. 3a). The saturated mode of binding indicates that these proteins have specific binding sites that become saturated at low plasma concentrations (Fig. 3b). Cluster IV and V have a variable binding pattern, while proteins in cluster VI mostly consist of saliva proteins that are outcompeted by increasing plasma concentration at around 1% plasma (Fig. 3a). The saturated mode of binding observed at relatively low concentrations of plasma possibly reflects the typical situation encountered in vivo during plasma leakage at local infection sites.

**Fig. 4:**
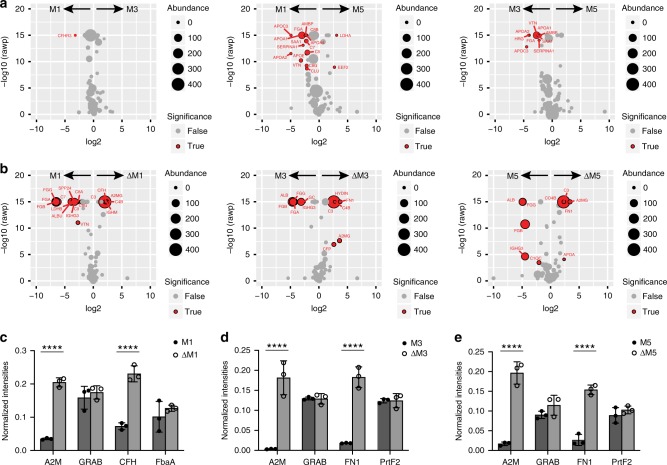
Plasma protein interactomes of *S. pyogenes* M1, M3, and M5 serotypes. **a** Volcano plots comparing the plasma interactomes of the different M protein serotype strains, and **b** volcano plots of the different M protein serotypes compared to their respective M protein deletion strains. In **a**, **b** human proteins are indicated that do (red sphere) and do not (gray sphere) show a significant difference in the compared interactomes. The data was filtered using a log2 fold enrichment of >2 and an adjusted *P-*value < 0.05 using the Welch’s *t*-test. The size of the spheres indicates protein abundance as measured in SA–DIA. **c–e** Quantitative Baccus peptide-based^8^ SRM-MS comparison between the different M protein serotypes as compared to their respective M protein deletion strains. Here we compare the interaction with human alpha-2-macroglobulin (A2M) in relation to the amount of expressed streptococcal protein GRAB, the amount of complement factor H (CFH) and fibronectin (FN1) in relation to fibronectin-binding proteins (FabA and Prtf2). Two-way ANOVA of the data indicates that there is a significant difference in the binding of the human proteins between the different strains as indicated by four asterisks *P* < 0.0001 using Sidak’s multiple comparison test and *n* = 3 biologically independent samples. Error bars are expressed as standard deviation (s.d.) from the mean. Source data are provided as a Source Data file

In the second experiment, we compared the interaction networks in plasma formed around three M protein serotypes: M1 strain (SF370), with a wild-type *csrR* regulon genotype; M3 (950771); and M5 (Manfredo; Fig. 4). Collectively, the M1 and M3 serotypes have been estimated to account for a third of all clinical isolates in high-income countries in the world, and are prevalent in cases of invasive disease^30^, while the less common M5 strains are associated with acute rheumatic fever^31^. SA–DIA experiments with the M1, M3, and M5 strains in human plasma revealed that the surface interaction maps are similar, although there are areas of statistically significant differences; for example, M1 binds more of complement factor H-related protein 3 (CFHR3) compared to M3 (Fig. 4a). SF370 binds many of the interactors identified with AP1 (Fig. 2), and which have reduced binding profiles in M5, such as fibrinogen chain alpha (FGA), apolipoproteins, and several components of the complement MAC (Fig. 4a). To further assess the role of M proteins in mediating these interactions, we performed additional SA–DIA experiments using isogenic M protein (*emm*) deletion strains (Fig. 4b). Removal of M protein leads to an increased binding of complement proteins C3 and C4, the broad-range protease inhibitor alpha-2-macroglobulin (A2M; Fig. 4b), and also a statistically significant increase of CFH on M1 and fibronectin (FN1) on M3 and M5. In our AP–DIA experiments we identified A2M as a highly specific binder of protein G-related A2M-binding protein (GRAB), as previously reported (Fig. 2, Supplementary Figs. 2, 8)^32^. Other reports have shown that CFH and FN1 binds to streptococcal FbaA and PrtF2, respectively^33,34^. To investigate the reason for the increased levels of A2M, CFH, and FN1, we performed selected-reaction monitoring MS (SRM-MS) on the interacting proteins and their target bacterial surface proteins (Fig. 4c–e). These results confirm the increased abundance of A2M, CFH, and FN1 on the *emm*-deletion strains, whereas their bacterial receptor proteins (GRAB, FbaA, and Prtf2) remain unchanged. We believe that the increased levels of A2M, CFH, and FN1 are mainly due to removal of steric hindrance by the M proteins and associated human binding proteins. The observation that removal of M protein results in a significant reduction in binding of human albumin, fibrinogen, and IgG3 is common to all serotypes. These results imply that IgG3 is strongly associated to the M proteins, prompting further investigation.

### IgG3 antibodies have high affinity to M proteins

**Fig. 5:**
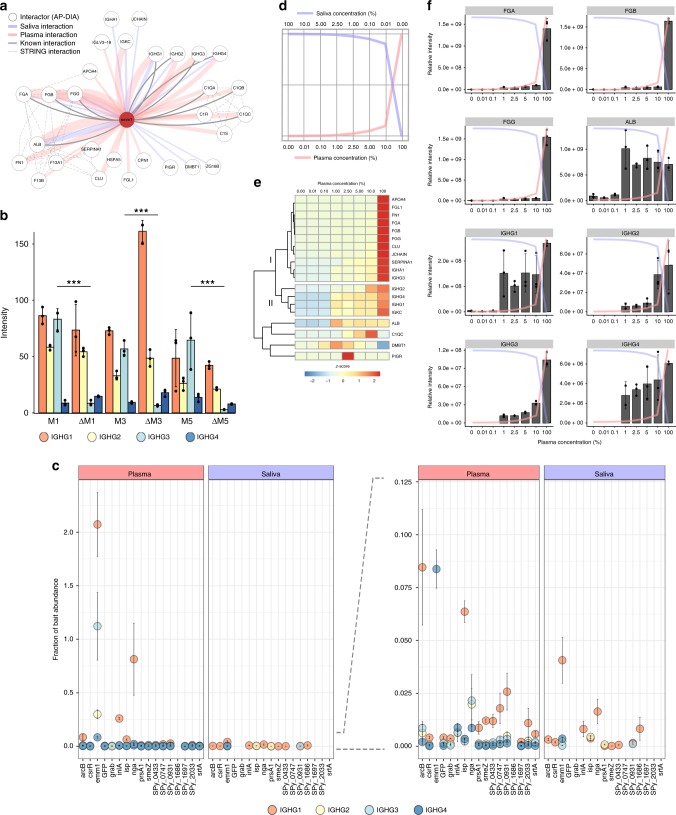
The M protein immunoglobulin interactomes. **a** The M1 protein–human interactome. Known human–human interactions from the STRING-database^70^ are depicted with broken gray lines. Previously identified M1–human interactions are depicted with solid gray lines, and are most recently described in Hauri et al.^17^. The interactome views were generated using Cytoscape^69^ and modified in Adobe Illustrator. **b** IgG subclass distribution between the different M protein serotypes and their respective deletion mutants. IgG3 is the only subclass for which the difference in binding to the wild-type strain as compared to the mutant strain is significant, as indicated with *P* values: *P < 0.05, **P < 0.01, and ***P < 0.001 using the Welch’s *t*-test. **c** IgG subclass distribution over the different streptococcal bait proteins used in this study. As is evident, the immunoglobulins have a pronounced affinity for the M protein (*emm1*). The dominating subclass interacting with the M1 protein is IgG1 (orange sphere) followed by IgG3 (shown in light blue). **d**–**f** AP–DIA in a mixed plasma–saliva environment mimicking vascular leakage. **d** Different mixtures of plasma in saliva (0.01%, 0.1%, 1%, 2.5%, 5%, and 10% plasma) were used to study the effect of the host environment on protein–protein interactions. **e** Cluster analysis of the M1 protein AP–DIA data in a mixed plasma and saliva environment based on *z*-score. **f** AP–DIA interaction patterns of fibrinogen (FGA, FGB, FGG), albumin (ALB) and the different IgG subclasses (IGHG1–4) in mixed plasma and saliva environments. Error bars are expressed as standard deviation (s.d.) from the mean. All experiments were prepared using *n* = 3 biologically independent samples. Source data are provided as a Source Data file

Our AP–DIA experiments (Fig. 2) identify a total of 29 human proteins interacting with M1 protein, several of which are known binders, such as fibrinogen and albumin; however, there are also other human proteins that have not previously been reported to have this association (Fig. 5a). Furthermore, we observe a strong association between the M proteins and all different IgG subclasses, compared to the other bait proteins (Fig. 5b, c). Previous work has indicated that *S. pyogenes* had a strong preference for IgG3s over other subclasses (IgG1–2 and 4)^8^. Here, we provide two additional lines of evidence indicating that IgG3 is the predominant subclass of M protein-mediated Fab-bound antibodies. First, analysis of the IgG subclass distribution in the SA–DIA experiments from the different serotypes (Fig. 4), show a significant >90% reduction of IgG3 in the *emm*-deletion strains, while the other IgG subclasses remain relatively unchanged (Fig. 5b). Second, in additional AP–DIA experiments using the M1 protein and the mixed plasma/saliva samples, IgG3 does not display a saturated-binding profile, in contrast to the other subclasses of immunoglobulins (IgG1, IgG2, and IgG4; Fig. 5d–f). The pronounced high level of non-saturated IgG3 associated with the bacterial surfaces and M1 protein indicates that IgG3 might predominately bind via the Fab domain, whereas the binding of IgG1–2 and IgG4 is in part Fc-mediated to the M1 protein S-region^18^. With this explanation, IgG3 would not display a saturated binding pattern due to the larger number of surface epitopes available on the M1 protein, whereas the other subclasses binding via the Fc domain would be confined to a single binding site.

### Immune and non-immune binding of immunoglobulins

**Fig. 6:**
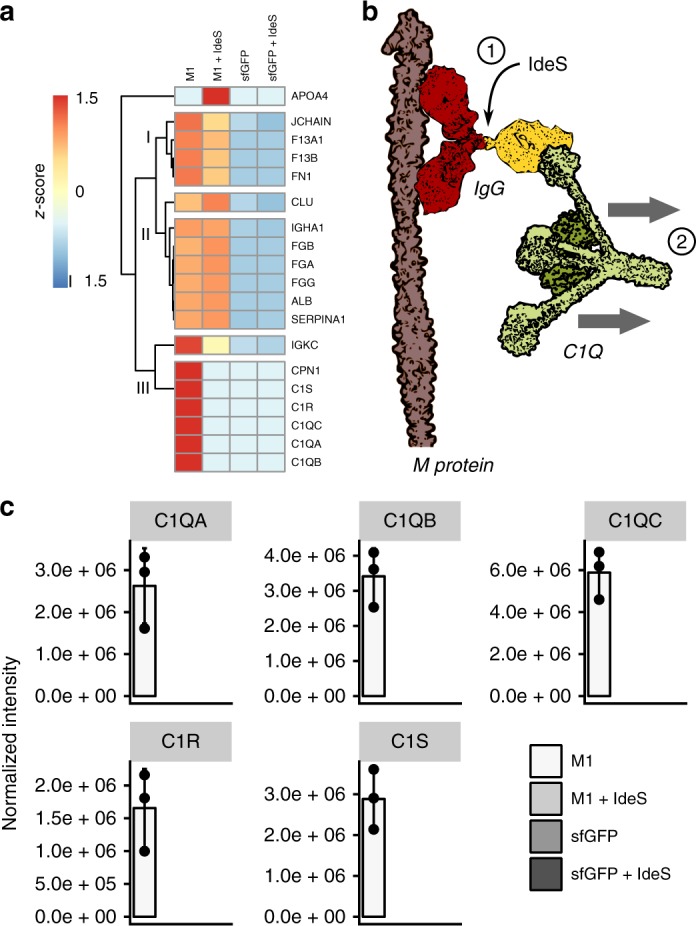
Plasma IdeS pre-treatment in AP–DIA. **a** Cluster analysis based on *z*-score of the M1 protein AP–DIA data in human plasma and IdeS pre-treated human plasma. sfGFP was used as a negative control. **b** Plasma IdeS cleavage counteracts IgG-mediated C1Q attachment to the M1 protein. **c** Effect of plasma IdeS pre-treatment on complement C1Q, C1R, and C1S binding to the M1 protein. Error bars are expressed as standard deviation (s.d.) from the mean. All experiments were prepared using *n* = 3 biologically independent samples. Source data are provided as a Source Data file

**Fig. 7:**
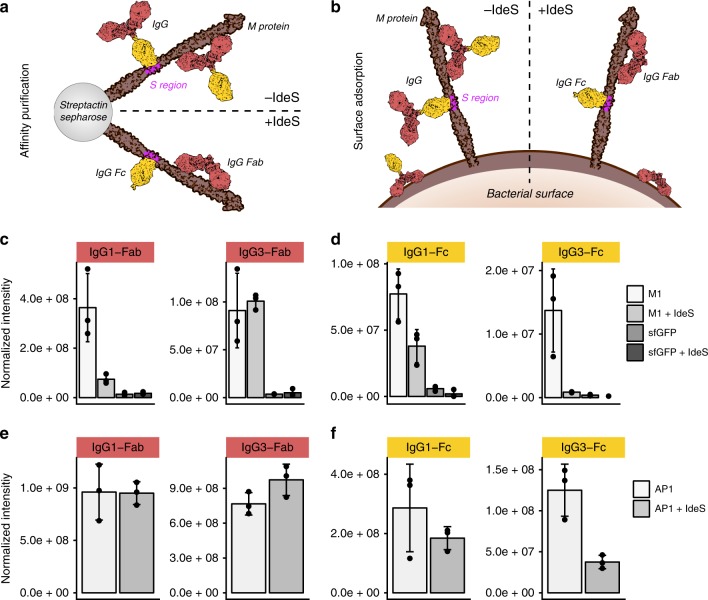
Plasma IdeS pre-treatment in AP–DIA and SA–DIA. **a** IgG binding in AP–DIA experiments in non-treated plasma and plasma pre-digested with IdeS. **b** IgG binding in SA–DIA experiments in non-treated plasma and plasma pre-digested with IdeS. **c**, **d** Quantification of Fab and Fc-specific signature peptides after IdeS cleavage using targeted MS analysis after affinity purification on the M1 protein. sfGFP was used as a negative control. **e**, **f** Quantification of Fab and Fc-specific signature peptides after IdeS cleavage using targeted MS analysis after affinity purification using whole wild-type M1 serotype AP1 bacteria. Error bars are expressed as standard deviation (s.d.) from the mean. All experiments were prepared using *n* = 3 biologically independent samples. Source data are provided as a Source Data file

To further determine whether the different subclasses of immunoglobulins predominantly bound to M1 protein and to the surface of *S. pyogenes* via the Fab or Fc fragment, we pretreated the plasma with IdeS to cleave the IgGs into Fab and Fc parts prior to AP/SA–DIA analysis. IdeS is a secreted and highly specific cysteine protease produced by *S. pyogenes* that cleaves human IgGs in the hinge region, while leaving IgA, IgD, IgE, and IgM molecules intact^19^. Removal of the Fc part from Fab-bound IgGs eliminates all Fc-facilitated binding and any downstream protein interactions mediated via Fc. Focusing first on the AP–DIA experiments, we observe that the M1 protein interactions with C1Q, C1S, and C1R proteins are completely abolished upon IdeS treatment and elution of the resulting Fc-domain (cluster III in Fig. 6a), demonstrating their IgG mediation (Fig. 6b, c). By monitoring specific peptides sequences specifically associated with either Fab or Fc fragments of the IgG1–4 subclasses^14^, we note a dramatic drop in the M1-coupled IgG1 Fab-fragment upon plasma IdeS digestion, whereas there is no difference in the binding profile of IgG3 Fab (Fig. 7a, c). The trend is the opposite for Fc bound IgG1 and IgG3, showing that the high levels of IgG3 observed in Figs. 2–5 is explained by Fab-bound IgG3, whereas IgG1 is partly Fc bound to the M1 protein (Fig. 7d). Notably, the levels of Fab-bound IgG2 and IgG4 to the M1 protein are too low for reliable quantitation and were thus omitted from the graphs. However, this observation further indicated that the predominant IgG classes interacting with M1 protein were IgG3 and IgG1.

By contrast, in the SA–DIA experiments, involving IdeS-digested plasma and the *S. pyogenes* serotype M1 strain AP1 (Fig. 7b), non-immune Fc and immune Fab-binding patterns of IgG1 and IgG3 were highly similar (Fig. 7e, f). IdeS treatment did not affect IgG1 Fab binding, most likely due to other available epitopes present on the bacterial surface in addition to M1 (Fig. 7e). Furthermore, several other known M1-binding proteins, such as fibrinogen and albumin, are unaffected by IdeS treatment during SA–DIA analysis (Supplementary Fig. 9). By contrast, there is also larger complexes adhering to the bacterial surface, where the binding is dependent on the presence of intact IgG molecules. For instance, all members of the complement MAC and the associated vitronectin and clusterin are reduced on the AP1 surface after pre-treatment with IdeS (Supplementary Fig. 9). These proteins likely bind as one complex, i.e. the fluid-phase SC5b-9^35^, as the intensity ratios between the components are similar in all samples (Supplementary Fig. 9). Taken together, the combined AP/SA–DIA results demonstrate that the primary M1 protein-mediated interaction with immunoglobulins is with Fab-bound IgG3, and that streptococci could potentially modify the host interaction network by secreting immunoglobulin degrading enzymes, such as IdeS, leading to diminished C1Q deposition and possible alterations in phagocytosis efficiency.

### The C-repeats of the M protein mediate phagocytosis

**Fig. 8:**
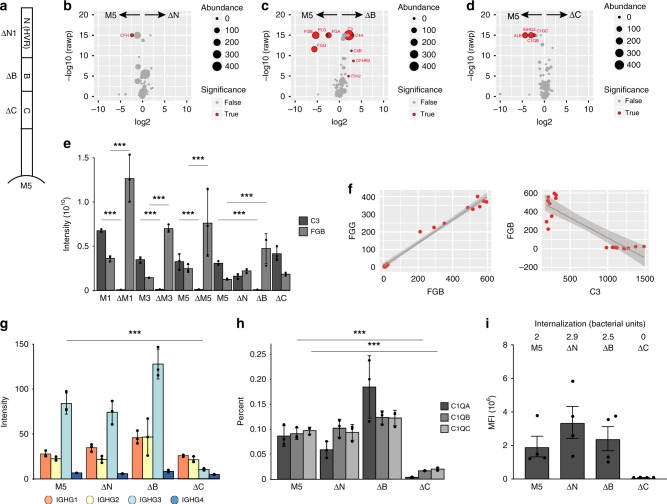
Localization of opsonizing antibodies on the M5 protein. **a** Schematic representation of the M5 protein, and the arrangement of the different domains (N, B, and C). Volcano plots comparing the SA–DIA plasma interactome of the wild-type M5 protein to the: **b** ΔN1 mutant strain, **c** ΔB mutant strain, and **d** ΔC mutant strain. In panels **b–d** a red sphere indicates a human protein with a significant difference in the compared interactomes, while a gray sphere indicates no significant difference, in the interaction between the wild-type and the respective mutant strain. The data was filtered using a log2 fold enrichment of >2 and an adjusted *P-*value < 0.05 using the Welch’s *t*-test. The size of the spheres indicates protein abundance as measured in SA–DIA. **e** A comparative analysis of the relationship between fibrinogen and complement C3 bound to the surface of the bacteria in all SA–DIA experiments used in this study. In all M protein mutant strains studied, the binding of fibrinogen to the surface of the bacteria is significantly reduced compared to the wild-type bacteria (indicated with ***P-value < 0.001 using the Welch’s *t*-test), as the binding of complement C3 increases on the surface of mutant bacteria. **f** Correlation analysis between fibrinogen and C3 reveals that the two proteins display a highly competitive mode of binding. **g** IgG subclass distribution between wild-type M5 strain and its different derivative mutant serotypes. IgG3 has specific affinity for the C-repeat region of the M5 protein. **h** C1QA-C association with wild-type M5 strain and the different derivative mutant serotypes. C1QB and C1QC both have specific affinity for the C-repeat region of the M5 protein, mediated by IgG3 Fc-binding. Q1QA was detected with one peptide only, Q1QB and C1QC each with two peptides. **i** Flow cytometry-based interaction (phagocytes associated with bacteria) and internalization (average number of bacteria inside phagocyte) of the wild-type M5 strain and different mutant strains with human THP-1 monocytes. The data is based on four separate experiments. Error bars in panels **e**, **g**, and **h** are expressed as standard deviation (s.d.) from the mean and in panel **i** as standard error of the mean (s.e.m). All experiments were prepared using *n* = 3 biologically independent samples, except in panel **i**, where *n* = 4 biologically independent samples were used. Source data are provided as a Source Data file

To further locate the M protein regions responsible for the IgG3 binding, we performed SA–DIA experiments using plasma and several M5 mutant strains, ΔN1, ΔB, and ΔC (Fig. 8a)^36^. Mutant strains without their hypervariable region (N) result in a slight but significant reduction of CFH (Fig. 8b). The mutant strains without B-repeats result in a significant reduction of fibrinogen and plasminogen (Fig. 8c), as previously shown^37^ and the mutant strains without C-repeats result in a significant reduction of albumin, C1Q, and IgG3 (Fig. 8d). The absence of the B-repeats significantly elevates the levels of C3 and C4, demonstrating that removal of the extensive fibrinogen network from the bacterial surface promote increased binding of C3 and C4 to the bacterial surface, possibly by providing a greater surface accessibility. To investigate this phenomenon in more detail, we compared the relationship between fibrinogen and C3 across all the SA–DIA experiments in this study. In all cases, a significant reduction of fibrinogen at the bacterial surface results in a significant increased binding of C3 (Fig. 8e). Correlation analysis between fibrinogen and C3 revealed that the two proteins displayed a highly competitive mode of binding, although they adhered to different parts of the streptococcal surface (Fig. 8f). Our observations were consistent with previous reports that fibrinogen inhibited complement C3 deposition, both on M protein and on the surface of bacteria^38^. In addition, these results demonstrate that the different binding domains of M proteins are associated with sets of proteins that can influence *S. pyogenes* propensity to become phagocytic. This raised the question as to what alters the degree of interaction and internalization by human phagocytes; whether it is loss of fibrinogen binding and subsequent increase of C3, or loss of IgG3 and C1Q, while the other subclasses of immunoglobulins remain constant (Fig. 8g, h). To investigate this, we performed comparative interaction and internalization analysis of the bacteria with the monocytic THP-1 cell line, using the serotype M5 and its mutant derivates ΔN1, ΔB, and ΔC, in pooled normal human plasma. Based on median fluorescence intensity (MFI), the interaction of intact M5 protein, and the ΔN1 and ΔB mutants with THP-1 cells are of the same order of magnitude, while that of the ΔC mutant is reduced >99% (Fig. 8i, Supplementary Fig. 10). Furthermore, when comparing the number of bacteria showing internalization, we do not observe it for the ΔC mutant, while the level in intact M5, and the ΔN1 and ΔB mutants are similar (Fig. 8i).

One of the leading candidates from current vaccine focus on M proteins^16,39^ is the J8-peptide, located in the C-region of M1^40^; the same region that is shown above to be crucial for interaction and internalization with monocytic cells (Fig. 8i). Enzyme-linked immunosorbent assays (ELISA) with synthetic peptides constructed from sequence repeats of the M1 protein in pooled intravenous immunoglobulins (IVIG) show that the immunoglobulins present have a preferential affinity for the C-repeat region containing the 12 amino acid J8-sequence, as compared to the B-regions (Supplementary Fig. 11). Hence, these results confirm that peptides in the C-region of M protein are potent epitopes for opsonizing antibodies^41^, and that these repeat regions are central targets for peptide-based vaccine development.

## Discussion

The development of vaccines and other treatment strategies to combat bacterial infections is dependent on knowledge of the natural human immune response against these pathogens. Exposure to streptococcal bacteria induces immune responses against members of the M protein family and other less studied streptococcal protein targets^42^. In this study, we use the quantitative aspects of DIA-MS to identify over 100 human blood plasma and saliva proteins targeted by *S. pyogenes* during the infection process. We identify several key components of the human immune system that are bound to the bacterial surface as larger protein complexes, and which are dependent on the site of infection. In saliva, *S. pyogenes* preferentially uses secreted proteins to target salivary defense proteins. This is consistent with a previous report where several streptococcal extracellular virulence factors were produced during growth in saliva^43^. However, in plasma, surface-attached proteins are used to target many important plasma proteins, both to sequester fatty acids via human serum albumin in the late stages of infection, leading to bacterial fatty acid synthesis being shut down and thus saving energy^2^, but also to bind host proteins to protect the bacteria against human immune defenses^8^. In a mixed plasma–saliva environment mimicking vascular leakage during an infection^2,14,15^, binding of saliva proteins is outcompeted by plasma proteins. This suggests that *S. pyogenes* is adapted to specifically sequester plasma proteins at low concentrations that might typically be found during plasma leakage at local infection sites.

We explore in detail the human interactions which are crucial for immune evasion and phagocytosis. For instance, in a dynamic setting aimed at resembling plasma leakage during tonsillitis and pharyngitis, we observe that binding of human plasma fibrinogen and fibronectin to the bacterial surface is saturated at very low plasma concentrations, indicating that this interaction is primarily a survival mechanism of *S. pyogenes* when causing infections in the upper respiratory tract. By binding a large, elongated molecule such as fibrinogen in a network-like structure^44^, the binding sites for IgGs, albumin, and other crucial interaction partners might be masked and made inaccessible. Indeed, it has been suggested that fibrinogen bound to the M5 protein promotes phagocytosis resistance by inhibiting complement deposition by the classical pathway, possibly by causing steric hindrance on the bacterial surface, thus inhibiting the formation of C3 convertase^38^. Our results confirm that fibrinogen and C3 binding are mutually exclusive. These collective observations of exclusive and non-exclusive protein–protein interactions in different ecological niches strengthens the notion that quantitative approaches in different states are needed to deduce molecular disease mechanisms^6^.

The AP–DIA and SA–DIA experiments identify M1 protein as an important binder of IgG-molecules, and both approaches demonstrate that Fab-mediated IgG3 binding is the predominant interaction. This is consistent with previous studies describing IgG1 and IgG3 as the most abundant subclasses against M protein^8,45,46^. Importantly, in immune responses, IgG1 and IgG3 are the most important IgGs in activating the complement cascade via the classical pathway^47^, and in triggering Fc-mediated phagocytosis^48^. However, the exact role of IgG3 in *S. pyogenes* infections still remains to be determined, even though there is broad evidence that IgG3 has a strong affinity for *S. pyogenes*. Notably, complement C1Q binding has previously been associated with the streptococcal surface^49,50^. Based on AP–DIA and SA–DIA, we show that enzymatic removal of IgG-Fc completely abolished the C1Q–M1 protein association, leading to decreased C3 deposition on the surface of the bacteria. This direct IgG-mediated association of C1Q to the M proteins has a subsequent impact on the interaction with monocytic cells. In addition, the J8-peptide located in the M protein C-region has a strong affinity for pooled human immunoglobulins as compared to the B-regions, with possible implications for immunoglobulin-based therapies for invasive streptococcal infections. Finally, in school-age children, pharyngitis caused by *S. pyogenes* is more common than in adults^42^, which could in part be explained by the lower levels of IgG3 and IFN-γ in children compared to adults^42^; again, possibly highlighting the importance of IgG3 in adaptive immunity against *S. pyogenes* infections.

In conclusion, this study provides a quantitative resource of protein interactions between *S. pyogenes* and human proteins, confirming several previously described interactions; however, it also provides new instances. The usefulness of the resource is demonstrated by investigating binding orientation and localization of IgG in an unbiased manner. Such information will be important in developing new targeted therapies or vaccines. The low level of protein conservation between bacterial virulence factors from different species prevents comparative protein analysis across species; however, many of the proteins bound to the streptococcal surface have also been reported to bind to virulence factors in other bacterial species. Clearly, similar mapping of protein interaction network in other bacterial species will allow for comparative analysis, potentially enhancing the future usefulness of the species-specific resources.

## Methods

### Cloning, protein expression, and purification

*S. pyogenes* open-reading frames (ORFs) encoding for the proteins M1 (Uniprot ID: Q99XV0, gene name: *emm1*), GRAB and its mutant derivates (Q7DAL7, *SPy_1357*), CovR (O87527, *csrR*), sortaseA (Q99ZN4, *SPy_1154*), ISP (Q99XU7, *SPy_2025*), and its truncated version ISP-N, as well as sfGFP used as a negative control in our AP experiments, were cloned, expressed, and purified at the Lund Protein Production Platform (LP3; Lund, Sweden). All ORFS were ordered as synthetic constructs from Genscript, USA, cloned into the EcoRV site of pUC57, and were subsequently subcloned into a pNIC28-Bsa4-based vector carrying the AP 6xHis-HA-StrepII-TEV (histidine-hemagglutinin-StrepII-tobacco etch virus protease recognition site) tag used in this study.

The proteins were expressed in Terrific Broth (TB; Difco) at 30 °C (GRAB, sortaseA, ISP, ISP-N) or 18 °C (CovR) in *Escherichia coli* TUNER (DE3) cells, with expression being induced with 1 mM Isopropyl β-d-1-thiogalactopyranoside (IPTG) at OD_600_ 0.5–0.7. Expressed cells were harvested and resuspended in phosphate buffer (25 mM sodium phosphate pH 8.0, 300 mM NaCl, 20 mM imidazole) supplemented with EDTA-free Complete Protease Inhibitor tablets (Roche). The cells were lysed using a French pressure cell at 18,000 psi. The lysate was cleared via ultracentrifugation (Ti 50.2 rotor, 244,000 × *g*, 60 min, 4 °C) and subsequently passed through a 0.45 μm filter prior to loading on a HisTrap HP column (GE Healthcare). The column was washed with 20 column volumes (CVs) of phosphate buffer, and bound protein was eluted using a gradient of 0–500 mM imidazole in phosphate buffer. Fractions containing the desired protein were pooled, and dialyzed against 1 × phosphate buffer saline (PBS; 10 mM phosphate buffer, 2.7 mM KCl, 137 mM NaCl) pH 7.3, after which the concentration was adjusted to 1 mg ml^−1^ using 1 × PBS pH 7.3, and stored at −80 °C.

ORFs encoding for the uncharacterized *S. pyogenes* proteins Q9A170 (*SPy_0433*), Q99YH8 (*SPy_1697*), Q99XU1 (*SPy_2033*), Q99YI6 (*SPy_1686*), P67274 (*SPy_0931*), as well as arcB (P0C0D0, *SPy_1544*), nga (Q7DAN2, *SPy_0165*), smeZ (Q99XW1, *SPy_1998*), prsA1 (P60811, *SPy_1390*), inlA (Q99Z76, *SPy_1361*), and spnA (Q9A0J7, *SPy_0747*) were expressed and purified at the Novo Nordisk Foundation Center for Protein Research, Copenhagen, Denmark. The DNA sequences were introduced into the expression vector pNIC28-Bsa4 by ligation-independent cloning (LIC)^51^. All proteins were expressed in TB at 18 °C overnight in *E. coli*, and expression was induced with 0.5 mM IPTG at OD_600_ 1.5. The cells were then harvested and resuspended in phosphate buffer (50 mM sodium phosphate pH 8.0, 300 mM NaCl, 10 mM imidazole, 10% glycerol, 0.5 mM *tris*(2-carboxyethyl)phosphine, TCEP) supplemented with EDTA-free Complete Protease Inhibitor tablets (Roche). The cells were lysed using a French pressure cell or sonication. The lysate was cleared via centrifugation and subsequently passed through a 0.22 μm filter prior to loading on a HisTrap HP column (GE Healthcare). Column elution and sample storage was as described above.

Protein M1 was expressed in Luria-Bertani Broth (LB; Difco) at 37 °C in *E. coli* BL21 (DE3) cells. Protein expression was induced with 1 mM IPTG at OD_600_ 0.5─0.6. Protein M1 was purified from harvested cells using a fibrinogen column^1,52^. Briefly, the cells were harvested and lysed using osmotic shock in 500 mM sucrose, 100 mM Tris–HCl, 1 mM EDTA pH 8.0. The cells were incubated on ice for 10 min prior to the addition of lysozyme and MgSO_4_ to final concentrations of 0.25 mg ml^−1^ and 10 mM, respectively. The cell debris was removed by centrifugation at 4 °C, 8500 × *g*, 30 min. The supernatant was collected and incubated with CNBr sepharose beads (GE Healthcare) coupled with human fibrinogen (Sigma). The column was washed with 8 CVs of 1 × PBS, and bound protein was eluted using 0.1 M glycine pH 2.0. Pooled fractions from the fibrinogen-column were dialyzed against 1 × PBS pH 7.4 and loaded on a Ni-coupled IMAC Sepharose 6 Fast Flow column (GE Healthcare). The column was washed with 20 mM imidazole in 1 × PBS pH 7.4, and bound protein was eluted with 500 mM imidazole in 1 × PBS pH 7.4 using gravity flow. Pooled fractions from the Ni-column were buffer exchanged into 1 × PBS pH 7.4 and concentrated using Millipore Amicon 10 or 30 kDa molecular weight cutoff concentrators. Purified protein was stored at −80 °C.

Protein names, Uniprot IDs, gene names, SPy-numbers, construct lengths (amino acids), location of affinity tag (N-terminal or C-terminal), reference to Protein Data Bank (PDB) structures (when relevant) and sequence coverage of the expressed constructs as determined by data-dependent acquisition (DDA) liquid chromatography tandem mass spectrometry (LC–MS/MS) are presented in Supplementary Data 1, and the overall protein purity as determined by DDA LC–MS/MS in Supplementary Data 2. The DNA sequences for the constructs are provided in Supplementary Data 7.

### Human plasma, saliva, and pooled immunoglobulins

Pooled human plasma (catalog number IPLA-N) and pooled human saliva (catalog number IR100044P) from healthy donors were purchased from Innovative Research, USA. Saliva was centrifuged at 1500 × *g* 15 min 4 °C, sterile filtered using 0.22 μm Steriflip filtration units (Millipore), concentrated to 5 mg ml^−1^ using Millipore Amicon 3.5 kDa molecular weight cut-off concentrators, and supplemented with Protease Inhibitor Cocktail (Sigma; 10 μl ml^−1^ saliva) prior to use. Pooled human IVIG (Octagam 100 mg ml^−1^, catalog number 158007) were obtained from Octapharma.

### AP in human plasma and saliva

AP reactions using pooled normal human plasma and saliva were essential as described^51^. Strep-Tactin Sepharose beads (IBA) were equilibrated in 1 × PBS pH 7.4, and charged with 10 μg of recombinant, affinity-tagged bait proteins. Affinity-tagged sfGFP was used as a negative control in all experiments. Pooled normal human plasma (100 μl) or saliva (200 μl) was incubated with the protein-charged beads at 37 °C, 800 rpm, 1 h. The saliva was complemented with 10 μl protease inhibitor (Sigma) per 1 ml of saliva. The beads were washed with 4 ml ice-cold PBS at 4 °C, and the proteins were eluted using 120 μl 5 mm biotin in 1 × PBS pH 7.4 at room temperature (RT). The samples were reduced, alkylated, and trypsin digested for MS as described below.

### AP in IdeS pre-treated human plasma

Pooled human plasma from healthy donors was treated with 20 μG IdeS (Hansa Medical AB) per 1 ml of plasma (37 °C, 800 rpm, 3 h). Subsequently, 1 μl 10 mM argatroban (Sigma-Aldrich), a thrombin inhibitor, was added per ml of plasma, and incubated for 10 min at RT to prevent plasma clotting. Untreated plasma supplemented with argatroban was used as a control. AP experiments were performed in triplicate with 10 μG affinity-tagged M1 protein as described above, using sfGFP as a negative control. To remove the biotin used in sample elution, the eluted samples were trichloroacetic acid (TCA) precipitated, washed with acetone, and dried in a speedvac. The TCA precipitated samples were reduced, alkylated, and trypsin digested for MS as described below.

### Removal of the affinity-tag via TEV-protease digestion

For reverse affinity-capture experiments using His-tagged human protein (see below), the affinity-tag attached to the streptococcal bait proteins M1, Q99XU1 (*SPy_2033*), and Q99YI6 (*SPy_1686*) was removed by TEV-protease digestion. Briefly, the bait proteins were digested with a 1:20 (μg μg^−1^) ratio of TEV–protease in the presence of 1 mM DTT in 1 × PBS pH 7.4 at 16 °C for 22 h. To retrieve the tag-free bait protein, the protease-treated samples were run over a 0.5 ml column prepared from IMAC Sepharose 6 Fast-flow beads (GE Healthcare) charged with Ni^2+^-ions according to the manufacturer’s protocol. The beads were equilibrated with 20 mM sodium phosphate, 0.5 M NaCl, 20 mM imidazole prior to sample loading, and retrieval using gravity-flow. In addition to the flowthrough fraction, three subsequent 0.5 ml wash fractions (20 mM sodium phosphate, 0.5 M NaCl, 20 mM imidazole) were collected, pooled, and stored at −80 °C.

### Reverse affinity-capture using His-tagged human serum albumin

The interaction of the streptococcal bait proteins M1, Q99XU1 (*SPy_2033*), and Q99YI6 (*SPy_1686*) with human serum albumin was verified by reverse affinity-capture, followed by sodium dodecyl sulfate-polyacrylamide gel electrophoresis (SDS–PAGE). IMAC Sepharose 6 Fast-flow beads (GE Healthcare) were charged with Ni^2+^-ions according to the manufacturer’s protocol. The beads were equilibrated in 1 × PBS pH 7.4, and charged with 10 μg affinity-tagged human serum albumin (Acro Biosystems). TEV-cleaved streptococcal bait proteins (10 μg each) were incubated with the protein-charged beads at 37 °C, 500 rpm, 30 min. Naked Ni^2+^-charged beads incubated together with the TEV-cleaved streptococcal protein were used as a negative control. The beads were washed with 8 ml ice-cold PBS buffer at 4 °C, and proteins were eluted using 120 μl 0.5 M imidazole in 1 × PBS pH 7.4. The samples were analyzed by SDS–PAGE using 4–20% Criterion TGX gels (Bio-Rad).

### Western blot analysis

The interactions between the streptococcal bait proteins CovR, ISP, and its truncated version ISP-N with human fibrinogen, and between the uncharacterized proteins Q99XU1 (*SPy_2033*) and Q99YI6 (*SPy_1686*) and human serum albumin were validated from AP reactions in human plasma by western blot analysis. The AP reactions were separated on a 4─20% Criterion TGX gel (Bio-Rad) and transferred to polyvinylidene difluoride (PVDF) membranes (Bio-Rad).

To detect fibrinogen binding by western blot analysis, the membrane was blocked with 2% bovine serum albumin (BSA) in 1 × PBS and 0.05% Tween-20 (1 × PBST) for 1 h at 37 °C, and then washed three times for 5 min with 1 × PBST. Purified mouse anti-human fibrinogen monoclonal antibody (BD Pharmingen, catalog number 555866) diluted 1:1000 with 2% BSA in 1 × PBST was added and incubated for 1 h at 37 °C. The membrane was washed three times for 5 min with 1 × PBST, and goat anti-mouse IgG horseradish peroxidase (HRP) conjugate (Bio-Rad, catalog number 172-1011) diluted 1:3000 with 2% BSA in 1 × PBST was added and incubated for 1 h at 37 °C. The membrane was then washed three times for 5 min with 1 × PBST. After washing, the membrane was developed using Clarity Western ECL substrate (Bio-Rad), and analyzed using a Chemidoc XRS (Bio-Rad).

To detect human serum albumin binding by western blot analysis, the same procedure was used, except 5% nonfat dry milk blotting-grade blocker (Bio-Rad), sheep anti-human serum albumin antibody (Bio-Rad, catalog number AHP102) diluted 1:1000, and rabbit anti-sheep IgG HRP conjugate (Bio-Rad, catalog number 5184-2504) diluted 1:3000 were used as blocking agent, primary antibody and secondary antibody, respectively. Uncropped and unprocessed scans of all blots are included in the Source Data file.

### Bacterial strains and culture conditions

*S. pyogenes* M1 serotype strain AP1 (40/58; *covS* truncated) was obtained from the World Health Organization (WHO) Collaborating Centre for Reference and Research on Streptococci, Prague, Czech Republic, and the M1 serotype strain SF370 from the American Type Culture Collection (ATCC; strain reference 700294), originally isolated from an infected wound. The *emm1* deletion mutant strain was originally developed from the *emm1* SF370 wild type strain^53^, the *emm3* deletion strain from the *emm3* wild type strain 950771^54^, and the *emm5* deletion strain from the *emm5* Manfredo wild type strain^55^. The *emm5* Manfredo-derived mutant strains ΔN1, ΔB, and ΔC have previously been described^36^. The bacteria were grown on blood agar plates or from single colonies in Todd–Hewitt (TH) broth supplemented with yeast extract at 0.3% (w/v; AP1) or 0.5% (w/v; M1 SF370, M3 950771 and M5 Manfredo and their respective mutants; THY-media). The bacteria were grown at 37 °C, in 5% CO_2_ to mid-logarithmic phase (OD_600 nm_ 0.4–0.5), harvested by centrifugation (2300 × *g*, 10 min, 4 °C), and the cell pellets were washed with a total of 3 ml 50 mM Tris–HCl, 150 mM NaCl, pH 7.6 (WB). The cells were redissolved in WB to a 1% concentration (700 μl per 10 ml of original culture), corresponding to 2 × 10^9^ colony forming units (CFU) per ml, and used for plasma adsorption experiments as described below.

### Plasma adsorption experiments

The plasma adsorption protocol has been described^8,9^. Briefly, 50 μl AP1 bacteria solution was mixed with 200 μl plasma, saliva, or dilutions of plasma in saliva (0.01%, 0.1%, 1%, 2.5%, 5%, and 10%), and incubated at 37 °C, 500 pm, 30 min. The plasma could be treated with IdeS and argatroban, and the saliva was complemented with protease inhibitors as described above. For studies using M1 SF370, M3 950771, and M5 Manfredo and their respective mutants, 150 μl of bacteria were used in 450 μl plasma. Bacteria that had adhered plasma and/or saliva proteins were harvested by centrifugation and washed with a total of 1.5 ml WB. The pellets were resuspended in 100 μl LC grade H_2_O and transferred to sample tubes containing 0.1 mm silica beads. The bacteria were lysed with a cell disruptor (MP Biomedicals FastPrep-96; 2 × 3 min, 1600 rpm), and dried in a speedvac. The lysed cells were reduced, alkylated and trypsin digested for MS as described below.

### Crosslinking of human plasma proteins on bacterial surfaces

For the crosslinking experiment, pooled normal human plasma was adsorbed onto the surface of *S. pyogenes* bacteria^17^. *S. pyogenes* strain SF370 was grown at 37 °C, 5% CO_2_ to mid-exponential phase (OD_620nm_ ∼ 0.4) in TH broth supplemented with 0.3% (w/v) yeast extract. The cells were harvested by centrifugation (3500×*g*, 5 min), washed with HEPES-buffer, recentrifuged and resuspended to an approximate concentration of 1 × 10^9^ colony forming units ml^−1^. Four hundred microliters of pooled normal human plasma was mixed with 100 μl of bacteria and incubated at 37 °C 30 min 500 rpm. The bacteria with adsorbed plasma proteins were harvested by centrifugation (5000 × *g*, 5 min) and washed three times with HEPES-buffer, and resuspended in 100 μl HEPES-buffer. Heavy/light disuccinimidylsuberate crosslinker (DSS-H12/D12, Creative Molecules Inc., www. creativemolecules.com) resuspended in dimethylformamide (DMF) was added to final concentrations of 0, 500, 2000, and 4000 μM and incubated for 30 min 37 °C 900 rpm. The crosslinking reaction was quenched with a final concentration of 50 mM ammonium bicarbonate at 37 °C 30 min 500 rpm. The bacterial surface proteins with attached plasma proteins were digested off with 2 μG trypsin (Promega), prior to cell debris removal by centrifugation (1000×*g*, 15 min) and subsequent supernatant recovery. Any remaining bacteria were killed by heat inactivation (85 °C, 5 min) prior to sample preparation for MS. The data analyzed here originates from the same set of samples as described in Hauri et al.^17^.

### Sample preparation for MS

The samples from AP and plasma adsorption experiments were mixed with 8 M urea and 100 mM ammonium bicarbonate, and the cysteine bonds were reduced with 5 mM TCEP (37 °C for 30 min) and alkylated with 10 mM iodoacetamide (22 °C for 60 min). Samples were diluted with 100 mM ammonium bicarbonate to a final urea concentration of 1.5 M, and sequencing grade trypsin (Promega) was added for protein digestion (37 °C for 18 h). Samples were acidified (to a final pH 3.0) with 10% formic acid, and the peptides subsequently purified with C18 reverse phase spin columns according to the manufacturer’s instructions (Microspin and Macrospin columns, Harvard Apparatus). Peptides were dried in a speedvac and reconstituted in 2% acetonitrile, 0.2% formic acid prior to mass spectrometric analyses.

### Liquid chromatography tandem mass spectrometry

All peptide analyses were performed on a Q Exactive Plus mass spectrometer (Thermo Scientific) connected to an EASY-nLC 1000 ultra-high-performance liquid chromatography system (Thermo Scientific). For DDA, peptides were separated on an EASY-Spray column (Thermo Scientific; ID 75 μm × 25 cm, column temperature 45 °C) operated at a constant pressure of 600 bar. A linear gradient from 5% to 35% acetonitrile in aqueous 0.1% formic acid was run for 60 min (cross-linked samples) or 120 min (affinity-purification and surface digestion samples) at a flow rate of 300 nl min^−1^. One full MS scan (resolution 70,000@200 m/z; mass range 400–1600 m/z) was followed by MS/MS scans (resolution 17,500@200 m/z) of the 15 most abundant ion signals. The precursor ions were isolated with 2 m/z isolation width and fragmented using higher-energy collisional-induced dissociation at a normalized collision energy of 30. Charge state screening was enabled, and precursors with an unknown charge state and singly charged ions were rejected. The dynamic exclusion window was set to 15 s and limited to 300 entries. The automatic gain control was set to 1e6 for both MS and MS/MS with ion accumulation times of 100 and 60 ms, respectively. The intensity threshold for precursor ion selection was set to 1.7e4.

For data-independent acquisition (DIA, DIA-MS), peptides were separated using an EASY-Spray column (Thermo Scientific; ID 75 μm × 25 cm, column temperature 45 °C) as described for the DDA analysis. A full MS scan (resolution 70,000 @200 m/z; mass range from 400 to 1200m/z) was followed by 32 MS/MS full fragmentation scans (resolution 35,000@200 m/z) using an isolation window of 26 m/z (including 0.5 m/z overlap between the previous and next window). The precursor ions within each isolation window were fragmented using higher-energy collisional-induced dissociation at a normalized collision energy of 30. The automatic gain control was set to 1e6 for both MS and MS/MS with ion accumulation times of 100 ms (MS) and 120 ms (MS/MS).

For crosslinked samples, peptides were additionally analyzed in high-resolution MS1 (hrMS1). The peptides were separated as in DDA and DIA analysis via C18 reverse phase chromatography using a 25 cm EASY-Spray column (column temperature 45 °C) with a linear gradient from 5% to 35% acetonitrile in aqueous 0.1% formic acid for 90 min at a flow rate of 300 nl min^−1^. High-resolution MS scans (*R* = 280,000) were acquired using automatic gain control (AGC) set to 1e6 and a fill time of 100 ms.

### AP–DIA and SA–DIA data analysis

MS raw data were converted to gzipped and Numpressed^56^ mzML using the tool msconvert from the ProteoWizard, v3.0.5930 suite^57^. Acquired spectra for the AP–DIA and the initial SA–DIA interactomes with the M1 serotype strain AP1 were analyzed using the search engine X! Tandem (2013.06.15.1-LabKey, Insilicos, ISB)^58^ against an in-house compiled database containing the *Homo sapiens* and *S. pyogenes* serotype M1 reference proteomes (UniProt proteome IDs UP000005640 and UP000000750, respectively), with the *S. pyogenes* Protein H added (UniProt ID P50470), yielding a total of 72,241 protein entries and an equal amount of reverse decoy sequences. For data-analysis using the other serotypes, the same database was used, expect that it was with the M3 (UniProt proteome ID UP000000564) and M5 (UniProt proteome ID UP000002591) proteomes. Fully tryptic digestion was used allowing one missed cleavage. Carbamidomethylation (C) was set to static and oxidation (M) to variable modifications, respectively. Mass tolerance for precursor ions was set to 20 ppm, and for fragment ions to 50 ppm. Identified peptides were processed and analyzed through the Trans-Proteomic Pipeline (TPP v4.7 POLAR VORTEX rev 0, Build 201403121010) using PeptideProphet^59^.

The spectral libraries were generated from the PeptideProphet data using the Fraggle–Franklin–Tramler pipeline^60^. The multi-level false discovery rate (FDR) was set at 1%, and the libraries were trimmed to include the 3–6 most intense transitions per assay. Spectral libraries were used by DIANA^61^ to analyze the DIA-MS data. Each analyte (unique peptide sequence, charge state and post-translational modification profile) in the spectral library was used to extract ion chromatograms. The quantitative value was calculated by integrating the ion current for each of the fragments under the peak. The DIA data was filtered as described using a bait to sfGFP log2 fold enrichment of >2 and an adjusted *P*-value < 0.01 using the Student’s *t*-test^6^. Additionally, all proteins passing the above selection criteria but identified by less than three peptides were omitted from the interaction maps.

### TX-MS data analysis

The crosslinked samples were analyzed used the TX-MS workflow^17^. Briefly, proteins with experimentally determined structures (Supplementary Data 4) were downloaded from the PDB. For GRAB and CovR, the RosettaCM protocol was used to generate tertiary structure models^62^, and the rest of the targets were analyzed without tertiary structures. For interacting protein pairs of interest (Supplementary Fig. 2), a machine-learning algorithm used the acquired hrMS1 data and a compendium of quaternary structure models to predict potential binding interfaces^63^. Each of the generated models was ranked by analyzing the presence of theoretical crosslinked peptides and fragment ion masses in DDA and DIA data. In DDA data analysis, all theoretical m/z values for fragments of crosslinked peptides across fragment-ion spectra (MS2) were ranked by crosslinked peptide sequence coverage. In DIA data analysis, the theoretical fragments were searched using a modified openSWATH workflow^64^, which identified high-confidence peak groups from co-eluting fragments of the crosslinked peptide pairs and the isotope-labeled cross-linked fragment ions to provide the strongest evidence of occurrence in the data^17^. The top scoring models were subsequently subjected to high-resolution flexible backbone protein docking^63^, and the resulting models used to define the final set of distance constraints. Finally, for proteins without structural information, all computational cross-links were analyzed used the DDA data as input, and putative interactions were manually inspected.

### Selected reaction monitoring MS

SRM assays were acquired from previously published SRM assay repositories^23,65^. Additional SRM assays were developed based on DDA data presented here with spectral library generation in Skyline^66^. SRM analyses were performed on a TSQ Quantiva triple quadrupole mass spectrometer (Thermo Scientific) connected to an EASY-nLC II liquid chromatography system (Thermo Scientific). Briefly, peptides were separated on an EASY-Spray column (Thermo Scientific; ID 75 μm × 15 cm, column temperature 45 °C). A two-step gradient of buffer B (100% acetonitrile, 0.1% formic acid) in buffer A (aqueous 0.1% formic acid) was applied at a flow rate of 300 nl min^−1^. In the first step a gradient of 5–15% of buffer B was run for 3 min followed by a 15–35% gradient of buffer B for 34 min. MS was operated in SRM mode with a spray voltage of 1.9 kV and an ion capillary temperature of 325 °C. Unit resolution was 0.7 Da full width at half maximum for both Q1 and Q3. Collision energies were obtained from Skyline and all measurements were performed without scheduling. For the data presented herein, around 800 transitions were measured per run using a cycle time of 1.6 s. Data was acquired using Xcalibur software (version 3.0.63). The data was analyzed in Skyline, and statistical significance at protein level was calculated from the average peptide contribution for each sample using a paired, two-tailed Student’s *t*-test.

The AP–DDA and AP–DIA, the SA–DDA and SA–DIA, and the SRM MS data were deposited in PeptideAtlas^67^ with the identifier PASS01167, and the TX-MS data was available for download from ProteomeXchange with the identifier PXD011969.

### Phagocytosis assays

Single colonies of the M5 strain and its mutant derivates, ΔN1, ΔB, and ΔC, were isolated from blood agar plates and cultured at 37 °C, 5% CO_2_ in 3% (w/v) TH broth supplemented with 0.5% (w/v) yeast extract to an exponential phase (optical density ~0.5 at 620 nm). The bacteria were washed three times with 3 ml total volume Na-medium (5.6 mM glucose, 127 mM NaCl, 10.8 mM KCl, 2.4 mM KH_2_PO_4_, 1.6 mM MgSO_4_, 10 mM HEPES, 1.8 mM CaCl_2_; pH adjusted to 7.3 with NaOH). The bacteria were heat inactivated (80 °C, 5 min) and subsequently stained at 37 °C for 30 min with 2 µg ml^−1^ DyLight^**TM**^ 650 (ThermoFisher). The bacteria were washed once in Na-medium and sonicated to disperse possible aggregates (VialTweeter; Hielscher). The bacteria were opsonized with 1% pooled citrated human plasma (VisuCon^**TM**^-F Frozen Normal Control Plasma) for 30 min at 37 °C. Opsonized samples were washed five times with a total volume of 5 ml Na-medium and the concentration of the bacteria was measured by flow cytometry.

Human monocytic cell line Tamm–Horsfall protein 1 (THP-1), used as a model phagocyte^68^, was cultured in l-glutamine and sodium bicarbonate containing RPMI 1640 medium (Sigma), supplemented with 10% fetal bovine serum (Gibco), 1% Penicillin–Streptomycin (ThermoFisher), and 2 mM GlutaMAX (Life Technologies) at 37 °C with 5% CO_2_. The cells were kept at 0.2–1 × 10^6^ cells ml^−1^ with over 95% viability, and were harvested at 0.7 × 10^6^ cells ml^−1^ for the assay. For the phagocytosis assay, the cell media was changed to Na-medium, and the cells labeled with a LIVE/DEAD™ Fixable Violet Dead Cell Stain Kit (ThermoFisher). The cell concentration was calculated by flow cytometry to obtain 10^5^ cells per sample. The cells were added to the bacteria on ice and directly transferred to a heating-block and incubated for 30 min at 37 °C. Final reaction volumes were 150 µl in Na-medium, with bacteria to cell ratio of 1, 2, 5, 10, 20, 50, 100, 150, and 200. Samples were fixed in 1% paraformaldehyde (PFA; ThermoFisher) overnight on ice. Post-fixation samples were incubated with 50 mM glycine and 5% BSA for 10 min at RT. Samples were stained for 30 min at RT with Fab-specific DyLight 488-conjugated AffiniPure F(ab’) Fragment Goat Anti-human IgG (1:1000, Jackson ImmunoResearch).

Data was acquired by flow cytometry (CytoFlex Beckman-Coulter; 15,000 events, threshold 50,000 FSC-H). FlowJo version 10.2 (Tree Star) and Prism version 7.0c (GraphPad) were used for data analysis. Live cells were gated on forward and side scatter, followed by excluding doublets by gating on FSC-H versus FSC-A; dead cells were excluded. Interaction was defined by cells positive for DyLight 650, and internalization by cells positive for DyLight 650 and negative for DyLight 488. The results were based on four different experiments. Interaction data was presented as DyLight 650 median fluorescence intensity (MFI). Internalization was presented as number of bacterial units ingested for cells positive for at least one bacterium. The signal of a bacterial unit was determined by measuring the MFI of single bacterial units. Internalization was determined by subtracting the attached bacteria from the total number of interacting bacteria, and the differences in interaction were normalized by using the MOP evoking half-maximal interaction for each experiment and strain.

### Synthetic peptides

Synthetic peptides with a C-terminal StrepII AP tag (WSHPQFEK) covering the B1-region, B2B3-region, and the C-region of the M1 protein were purchased from ProteoGenix, France. The 12 amino acid-long J8-vaccine trial peptide (SREAKKQVEKAL)^40^ is included in the C-region peptide. A GFP-based peptide was used as a negative control. The sequences for the peptides are provided in Supplementary Table 1.

### ELISA

In order to validate human IgG binding to different M1 protein regions ELISA was used. The recombinant, full-length M1 protein and the synthetic peptides from ProteoGenix (see above; all 10 μg ml^−1^) were immobilized on MaxiSorp 96-well ELISA plates (Thermo Fisher Scientific) overnight at 4 °C. The plates were washed three times with 1 × PBST, and blocked with 2% BSA in 1 × PBST for 1 h at 37 °C. The plates were washed again three times with 1 × PBST, and IVIG (4 mg ml^−1^) was added as a two-fold dilution series. The plates were incubated 1 h at 37 °C, washed three times with 1 × PBST and affinity-purified protein G HRP conjugate (Bio-Rad, catalog number 170-6425) diluted 1:3000 in 1 × PBST was added to the wells. The plates were incubated 1 h at 37 °C, washed three times with 1 × PBST and color developed with 2,2′-azino-di-(3-ethylbenzthiazoline sulfonic acid) (ABTS; Sigma) for 5 min at RT in the dark, prior to determining the absorbance at 415 nm. The GFP-based peptide was used as a negative control in the assays, and its absorbance values were subtracted from the experimental data prior to analysis in Prism version 8.0.2 (GraphPad). Data analysis used two-way analysis of variance (ANOVA) followed by Tukey’s multiple comparison tests. Statistical significance levels were set at *P* < 0.0332, *P* < 0.0021, *P* < 0.0002 and *P* < 0.0001.

### Reporting summary

Further information on research design is available in the Nature Research Reporting Summary linked to this article.

## Supplementary information

Supplementary_Information_file

Description of Additional Supplementary Files

Supplementary Data 1

Supplementary Data 2

Supplementary Data 3

Supplementary Data 4

Supplementary Data 5

Supplementary Data 6

Supplementary Data 7

Reporting Summary

Source Data File

## Data Availability

The mass spectrometry data have been deposited in the ProteomeXchange member repository PeptideAtlas with the identifier PASS01167. The TX-MS data presented here is from Hauri et al.^17^, and is available for download from ProteomeXchange with the identifier PXD011969. The source data underlying Figs. 1–8 and Supplementary Figs. 1–7 and 9–11 are provided as a Source Data file. A reporting summary for this Article is available as a Supplementary Information file. All other data supporting the findings of this study are available from the corresponding author on reasonable request.

## References

[1] Åkesson P, Schmidt KH, Cooney J, Björck L (1994). M1 protein and protein H: IgGFc- and albumin-binding streptococcal surface proteins encoded by adjacent genes. Biochem. J..

[2] Malmström J (2012). *Streptococcus pyogenes* in human plasma: adaptive mechanisms analyzed by mass spectrometry-based proteomics. J. Biol. Chem..

[3] Schweppe DK (2015). Host–microbe protein interactions during bacterial infection. Chem. Biol..

[4] Aebersold R, Mann M (2016). Mass-spectrometric exploration of proteome structure and function. Nature.

[5] Nicod C, Banaei-Esfahani A, Collins BC (2017). Elucidation of host-pathogen protein–protein interactions to uncover mechanisms of host cell rewiring. Curr. Opin. Microbiol..

[6] Collins BC (2013). Quantifying protein interaction dynamics by SWATH mass spectrometry: application to the 14-3-3 system. Nat. Methods.

[7] Gillet LC (2012). Targeted data extraction of the MS/MS spectra generated by data-independent acquisition: a new concept for consistent and accurate proteome analysis. Mol. Cell. Proteom..

[8] Sjöholm Kristoffer, Kilsgård Ola, Teleman Johan, Happonen Lotta, Malmström Lars, Malmström Johan (2017). Targeted Proteomics and Absolute Protein Quantification for the Construction of a Stoichiometric Host-Pathogen Surface Density Model. Molecular & Cellular Proteomics.

[9] Sjöholm K, Karlsson C, Linder A, Malmström J (2014). A comprehensive analysis of the *Streptococcus pyogenes* and human plasma protein interaction network. Mol. Biosyst..

[10] Mitchell TJ (2003). The pathogenesis of streptococcal infections: from tooth decay to meningitis. Nat. Rev. Microbiol..

[11] Carapetis JR, Steer AC, Mulholland EK, Weber M (2005). The global burden of group A streptococcal diseases. Lancet Infect. Dis..

[12] Ralph AP, Carapetis JR (2013). Group a streptococcal diseases and their global burden. Curr. Top. Microbiol. Immunol..

[13] Malmström E (2016). Large-scale inference of protein tissue origin in gram-positive sepsis plasma using quantitative targeted proteomics. Nat. Commun..

[14] Karlsson Christofer A. Q., Järnum Sofia, Winstedt Lena, Kjellman Christian, Björck Lars, Linder Adam, Malmström Johan A. (2018). Streptococcus pyogenesInfection and the Human Proteome with a Special Focus on the Immunoglobulin G-cleaving Enzyme IdeS. Molecular & Cellular Proteomics.

[15] Wollein Waldetoft K (2016). Saliva-induced clotting captures streptococci: novel roles for coagulation and fibrinolysis in host defense and immune evasion. Infect. Immun..

[16] Sanderson-Smith M (2014). A systematic and functional classification of *Streptococcus pyogenes* that serves as a new tool for molecular typing and vaccine development. J. Infect. Dis..

[17] Hauri S (2019). Rapid determination of quaternary protein structures in complex biological samples. Nat. Commun..

[18] Nordenfelt P (2012). Antibody orientation at bacterial surfaces is related to invasive infection. J. Exp. Med..

[19] Pawel-Rammingen von U, Johansson BP, Björck LIdeS (2002). a novel streptococcal cysteine proteinase with unique specificity for immunoglobulin G. EMBO J..

[20] Collin M, Olsén A (2001). EndoS, a novel secreted protein from *Streptococcus pyogenes* with endoglycosidase activity on human IgG. EMBO J..

[21] Sjögren J (2013). EndoS2 is a unique and conserved enzyme of serotype M49 group A Streptococcus that hydrolyses N-linked glycans on IgG and α1-acid glycoprotein. Biochem. J..

[22] Nelson DC, Garbe J, Collin M (2011). Cysteine proteinase SpeB from *Streptococcus pyogenes*—a potent modifier of immunologically important host and bacterial proteins. Biol. Chem..

[23] Karlsson C, Malmström L, Aebersold R, Malmström J (2012). Proteome-wide selected reaction monitoring assays for the human pathogen *Streptococcus pyogenes*. Nat. Commun..

[24] Kilsgård O, Karlsson C, Malmström E, Malmström J (2016). Differential compartmentalization of *Streptococcus pyogenes* virulence factors and host protein binding properties as a mechanism for host adaptation. Int. J. Med. Microbiol..

[25] Malmström L (2015). Quantitative proteogenomics of human pathogens using DIA-MS. J. Proteom..

[26] Podack ER, Esser AF, Biesecker G, Müller-Eberhard HJ (1980). Membrane attack complex of complement: a structural analysis of its assembly. J. Exp. Med..

[27] Åkesson P, Sjöholm AG, Björck L (1996). Protein SIC, a novel extracellular protein of *Streptococcus pyogenes* interfering with complement function. J. Biol. Chem..

[28] Serna M, Giles JL, Morgan BP, Bubeck D (2016). Structural basis of complement membrane attack complex formation. Nat. Commun..

[29] Berends ETM (2013). Distinct localization of the complement C5b-9 complex on Gram-positive bacteria. Cell Microbiol..

[30] Steer AC, Law I, Matatolu L, Beall BW, Carapetis JR (2009). Global emm type distribution of group A streptococci: systematic review and implications for vaccine development. Lancet Infect. Dis..

[31] Bessen D, Jones KF, Fischetti VA (1989). Evidence for two distinct classes of streptococcal M protein and their relationship to rheumatic fever. J. Exp. Med..

[32] Rasmussen M, Müller HP, Björck L (1999). Protein GRAB of *Streptococcus pyogenes* regulates proteolysis at the bacterial surface by binding alpha2-macroglobulin. J. Biol. Chem..

[33] Jaffe J, Natanson-Yaron S, Caparon MG, Hanski E (1996). Protein F2, a novel fibronectin-binding protein from *Streptococcus pyogenes*, possesses two binding domains. Mol. Microbiol..

[34] Terao Y, Okamoto S, Kataoka K, Hamada S, Kawabata S (2005). Protective immunity against *Streptococcus pyogenes* challenge in mice after immunization with fibronectin-binding protein. J. Infect. Dis..

[35] Choi NH, Nakano Y, Tobe T, Mazda T, Tomita M (1990). Incorporation of SP-40,40 into the soluble membrane attack complex (SMAC, SC5b-9) of complement. Int. Immunol..

[36] Sandin C, Carlsson F, Lindahl G (2006). Binding of human plasma proteins to *Streptococcus pyogenes* M protein determines the location of opsonic and non-opsonic epitopes. Mol. Microbiol..

[37] Waldemarsson J, Stålhammar-Carlemalm M, Sandin C, Castellino FJ, Lindahl G (2009). Functional dissection of *Streptococcus pyogenes* M5 protein: the hypervariable region is essential for virulence. PLoS. One.

[38] Carlsson F, Sandin C, Lindahl G (2005). Human fibrinogen bound to *Streptococcus pyogenes* M protein inhibits complement deposition via the classical pathway. Mol. Microbiol..

[39] Schodel F (2017). Clinical development strategy for a candidate group A streptococcal vaccine. Vaccine.

[40] Batzloff MR (2003). Protection against group A streptococcus by immunization with J8-diphtheria toxoid: contribution of J8- and diphtheria toxoid-specific antibodies to protection. J. Infect. Dis..

[41] Brandt ER (1996). Opsonic human antibodies from an endemic population specific for a conserved epitope on the M protein of group A streptococci. Immunology.

[42] Mortensen R (2015). Adaptive immunity against *Streptococcus pyogenes* in adults involves Increased IFN-γ and IgG3 responses compared with children. J. Immunol..

[43] Shelburne SA (2005). Growth characteristics of and virulence factor production by group A Streptococcus during cultivation in human saliva. Infect. Immun..

[44] Macheboeuf P (2011). Streptococcal M1 protein constructs a pathological host fibrinogen network. Nature.

[45] Falconer AE (1993). Distinct IgG1 and IgG3 subclass responses to two streptococcal protein antigens in man: analysis of antibodies to streptolysin O and M protein using standardized subclass-specific enzyme-linked immunosorbent assays. Immunology.

[46] Retnoningrum DS, Podbielski A, Cleary PP (1993). Type M12 protein from *Streptococcus pyogenes* is a receptor for IgG3. J. Immunol..

[47] Brüggemann M (1987). Comparison of the effector functions of human immunoglobulins using a matched set of chimeric antibodies. J. Exp. Med..

[48] Nimmerjahn F, Ravetch JV (2008). Fcγ receptors as regulators of immune responses. Nat. Rev. Immunol..

[49] Koroleva IV, Sjöholm AG, Schalén C (1998). Binding of complement subcomponent C1q to *Streptococcus pyogenes*: evidence for interactions with the M5 and FcRA76 proteins. Fems. Immunol. Med. Microbiol..

[50] Koroleva IV, Schalén C, Sjoholm A (1997). Binding of C1q to group A streptococcal M-family proteins. Adv. Exp. Med. Biol..

[51] Wisniewska M (2014). Functional and structural properties of a novel protein and virulence factor (protein sHIP) in *Streptococcus pyogenes*. J. Biol. Chem..

[52] Collin M, Olsén A (2000). Generation of a mature streptococcal cysteine proteinase is dependent on cell wall-anchored M1 protein. Mol. Microbiol..

[53] Abbot EL (2007). Pili mediate specific adhesion of *Streptococcus pyogenes* to human tonsil and skin. Cell Microbiol..

[54] Pérez-Caballero D (2004). Interaction between complement regulators and *Streptococcus pyogenes*: binding of C4b-binding protein and factor H/factor H-like protein 1 to M18 strains involves two different cell surface molecules. J. Immunol..

[55] Johnsson E (1998). Role of the hypervariable region in streptococcal M proteins: binding of a human complement inhibitor. J. Immunol..

[56] Teleman J (2014). Numerical compression schemes for proteomics mass spectrometry data. Mol. Cell. Proteom..

[57] Chambers MC (2012). A cross-platform toolkit for mass spectrometry and proteomics. Nat. Biotechnol..

[58] Craig R, Beavis RC (2003). A method for reducing the time required to match protein sequences with tandem mass spectra. Rapid Commun. Mass Spectrom..

[59] Keller A, Nesvizhskii AI, Kolker E, Aebersold R (2002). Empirical statistical model to estimate the accuracy of peptide identifications made by MS/MS and database search. Anal. Chem..

[60] Teleman Johan, Hauri Simon, Malmström Johan (2017). Improvements in Mass Spectrometry Assay Library Generation for Targeted Proteomics. Journal of Proteome Research.

[61] Teleman J (2015). DIANA–algorithmic improvements for analysis of data-independent acquisition MS data. Bioinformatics.

[62] Alford RF (2017). The rosetta all-atom energy function for macromolecular modeling and design. J. Chem. Theory Comput..

[63] Gray JJ (2006). High-resolution protein–protein docking. Curr. Opin. Struct. Biol..

[64] Röst HL (2014). OpenSWATH enables automated, targeted analysis of data-independent acquisition MS data. Nat. Biotechnol..

[65] Malmström L, Marko-Varga G, Westergren-Thorsson G, Laurell T, Malmström J (2006). 2DDB—a bioinformatics solution for analysis of quantitative proteomics data. BMC Bioinforma..

[66] Maclean B (2010). Skyline: an open source document editor for creating and analyzing targeted proteomics experiments. Bioinformatics.

[67] Desiere F (2006). The PeptideAtlas project. Nucleic Acids Res..

[68] Tsuchiya S (1980). Establishment and characterization of a human acute monocytic leukemia cell line (THP-1). Int. J. Cancer.

[69] Su G, Morris JH, Demchak B, Bader GD (2014). Biological network exploration with Cytoscape 3. Curr. Protoc. Bioinforma..

[70] Szklarczyk D (2015). STRING v10: protein–protein interaction networks, integrated over the tree of life. Nucleic Acids Res..

