# Supplementary material for: A quantitative Streptococcus pyogenes–human protein–protein interaction map reveals localization of opsonizing antibodies
Source: Nat Commun. 2019 Jun 21;10:2727. doi: 10.1038/s41467-019-10583-5 (PMC6588558; doi:10.1038/s41467-019-10583-5)
Supplement: Supplementary file 1 — Supplementary_Information_file [file 41467_2019_10583_MOESM1_ESM.pdf]

Supplementary Information

Happonen *et al*.

A quantitative *Streptococcus pyogenes*-human protein-protein interaction map reveals localization of opsonizing antibodies

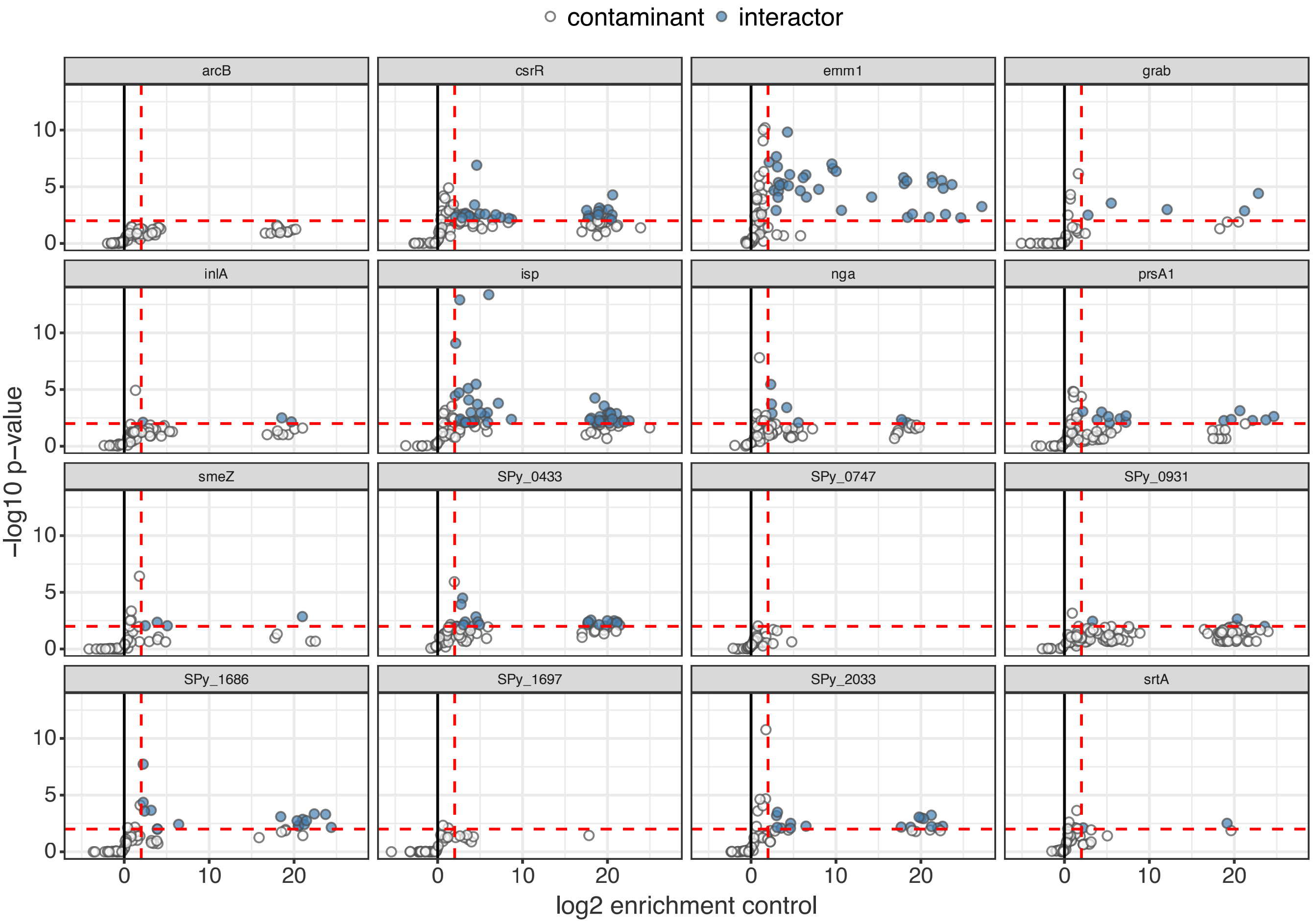

Supplementary Figure 1. Contaminant filtering of the AP-DIA data. The filtering is shown for each bait separately, with the P value on the y-axis and the enrichment in relation to sfGFP on the x-axis. The P value was calculated using the Student's t-test using n=3 biologically independent samples. Blue dots indicate high-confidence interactions while white dots indicate proteins falling below the set thresholds[1]. Error bars are expressed as standard deviation (s.d.) from the mean. Source data are provided as a Source Data file.

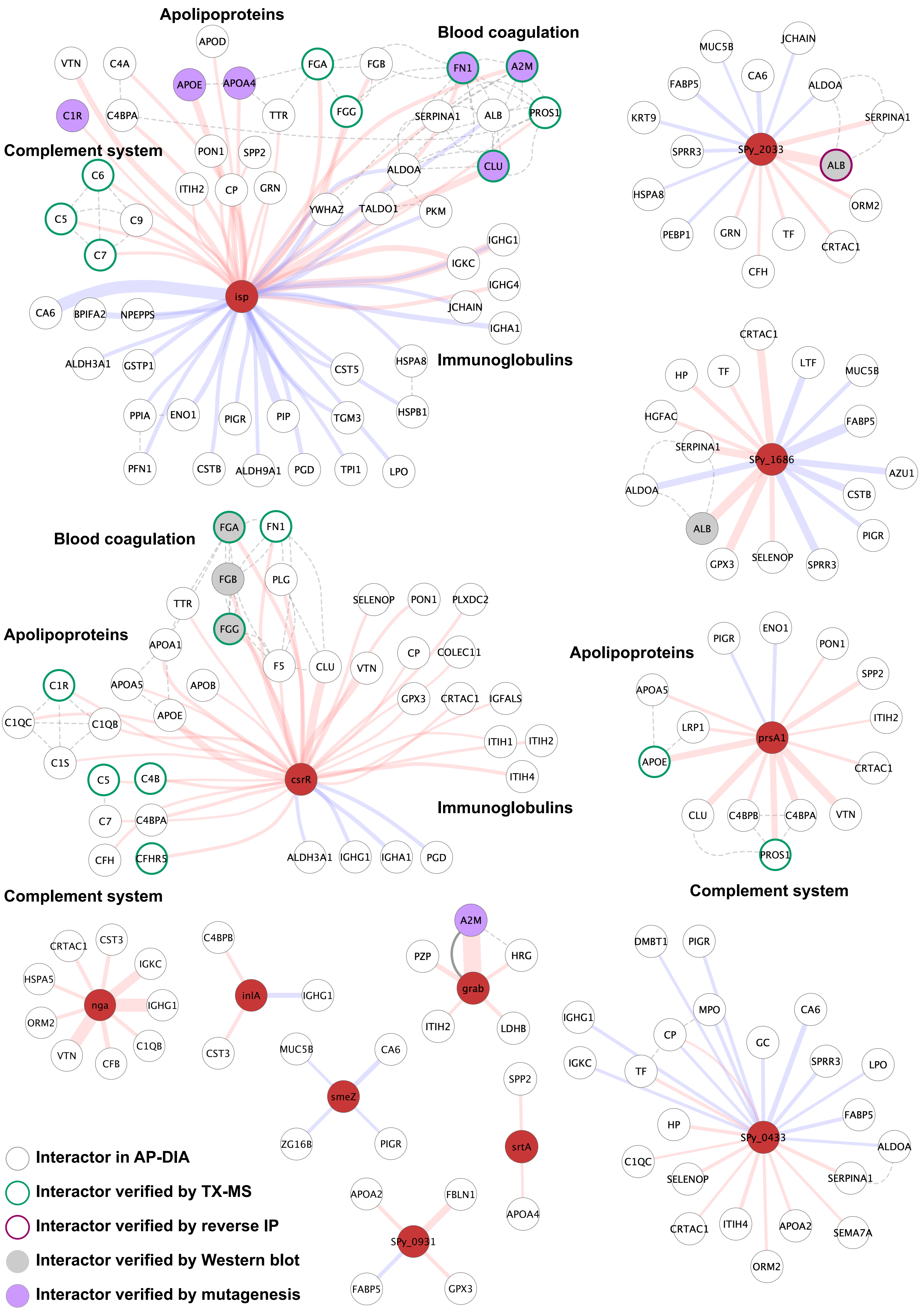

Supplementary Figure 2. Individual AP-DIA interactomes for the streptococcal bait proteins presented in Figure 2. The AP-DIA interactomes are represented for human plasma proteins (red) and saliva proteins (blue). The thickness of the edges connecting the nodes indicates the amount of interacting human protein in the samples. The dotted lines represent interactions between human proteins, as reported in the STRING database2. Closed grey lines represent previously reported streptococcal - human interactions; the only one described to date is that between GRAB and alpha-2-macroglobulin (A2M)3. Nodes with grey borders are interactions identified in plasma or saliva, nodes with green borders are interactions verified by TX-MS experiments in SA-DIA, nodes with purple borders are interactors verified by reverse affinity-capture, nodes with grey filling are interactions verified by western blot analysis, and nodes with purple filling are interactions verified by deletion mutagenesis.

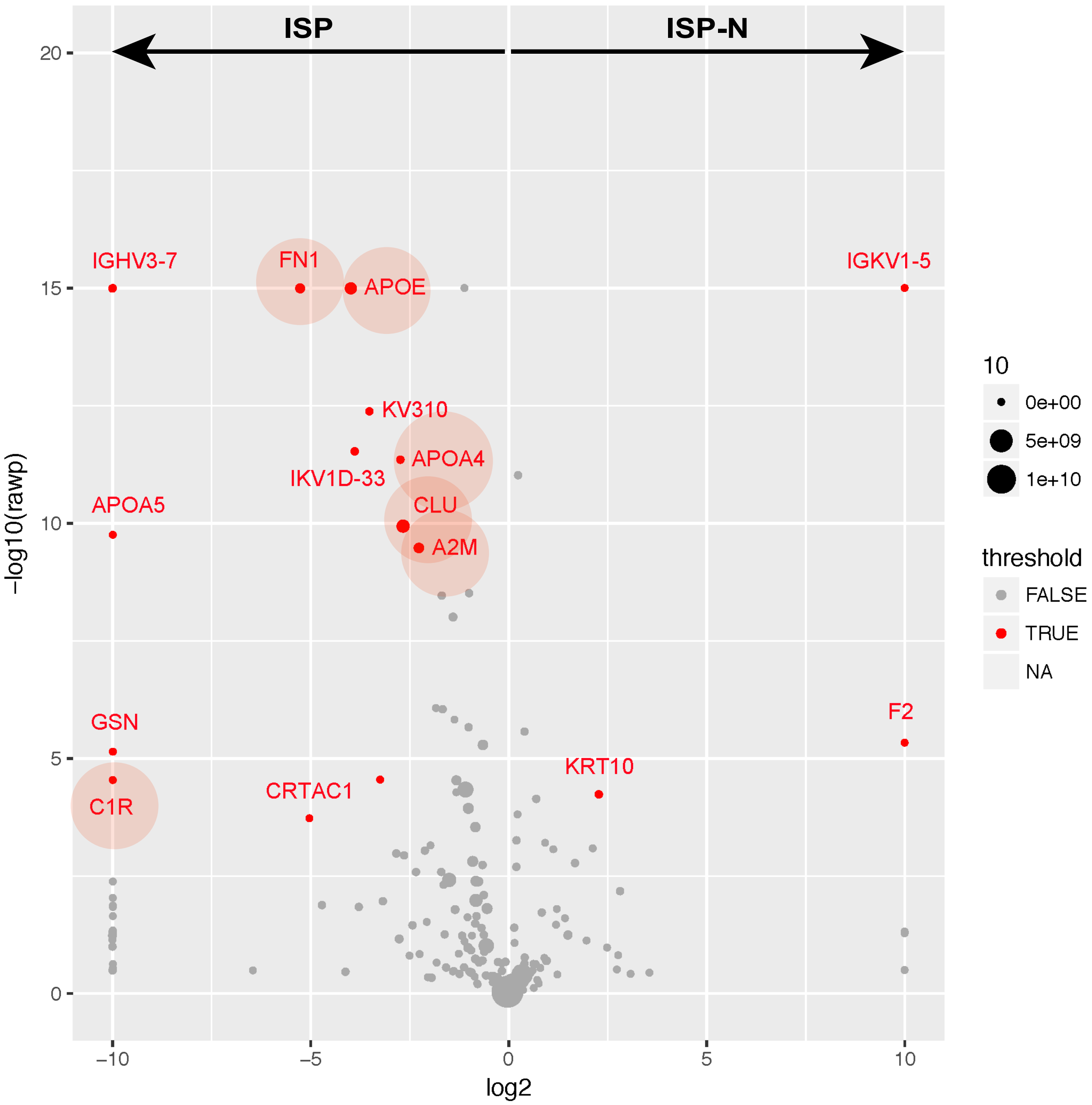

Supplementary Figure 3. Volcano plot comparing the AP-DIA plasma interactome of the wild-type ISP protein to the ISP-N deletion mutant. A red sphere indicates a human protein with a significant difference in the compared interactomes, whereas a grey sphere indicates no significant difference. The data was filtered using a log2 fold enrichment of >2 and a Hochberg adjusted P value < 0.05 calculated using the Welch's t-test; but plotted on a y-axis showing raw P-values. The experiment was prepared using n=3 biologically independent samples. The size of the spheres indicates protein abundance as measured in AP-DIA. Data points with a red halo indicate proteins present in the original ISP interactome in Figs. 2 and Supplementary Fig. 2. Source data are provided as a Source Data file.

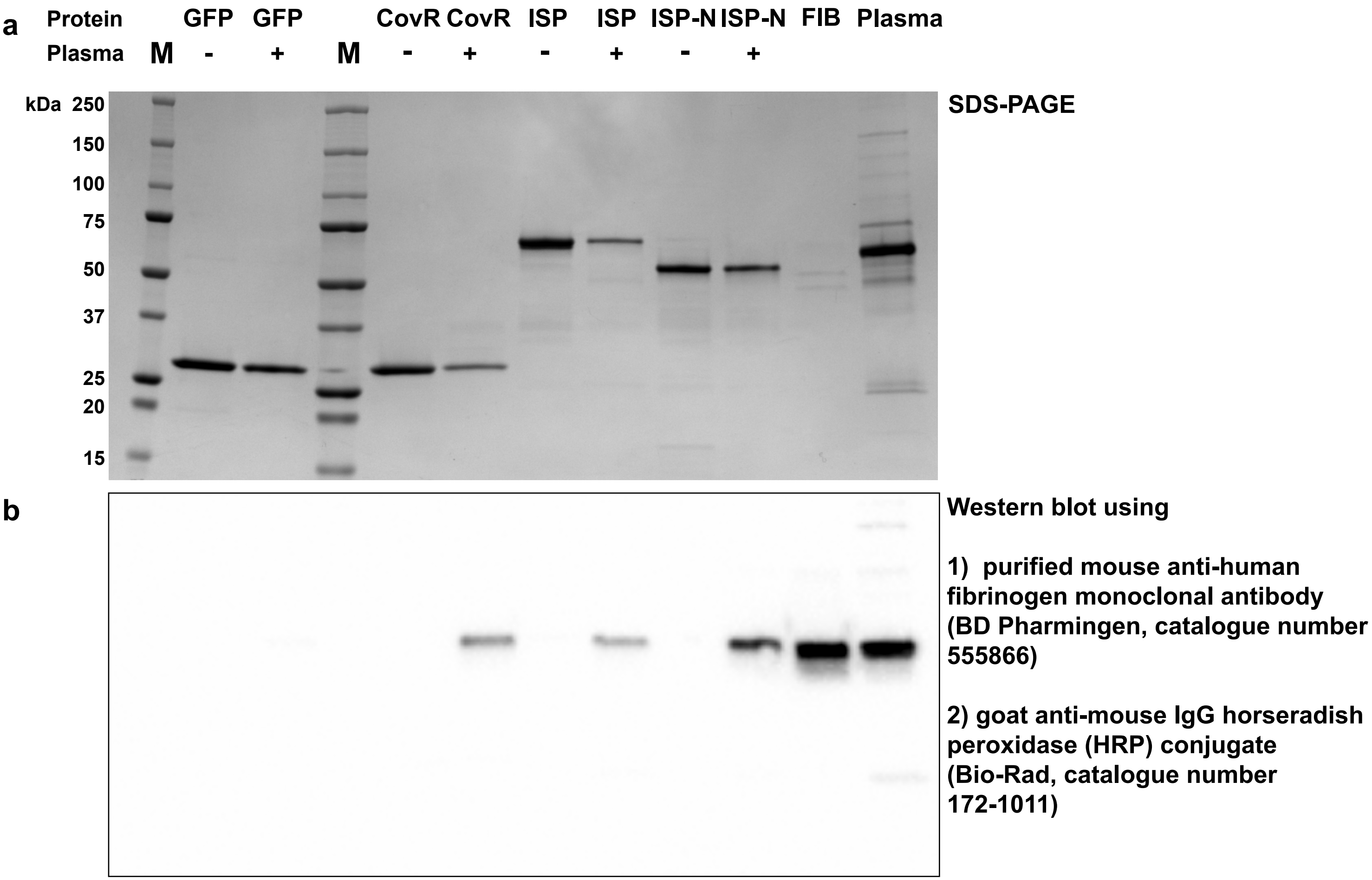

Supplementary Figure 4. Western blot analysis of fibrinogen binding to proteins CovR, ISP and ISP-N in AP reactions in human plasma. a) Coomassie blue stained SDS-PAGE gel showing the AP reactions. b) Western blot analysis of the same reactions probing for fibrinogen binding. sfGFP was used as a negative control. In a) and b) M indicates markers (Precision Plus Protein Dual Xtra Prestained Protein Standard, 2–250 kDa size range); FIB, fibrinogen from human plasma (Sigma), 1 ug; and Plasma, pooled human plasma used in the AP reactions, 3 ug. The uncropped SDS-PAGE gel and western blot are provided in the Source Data file.

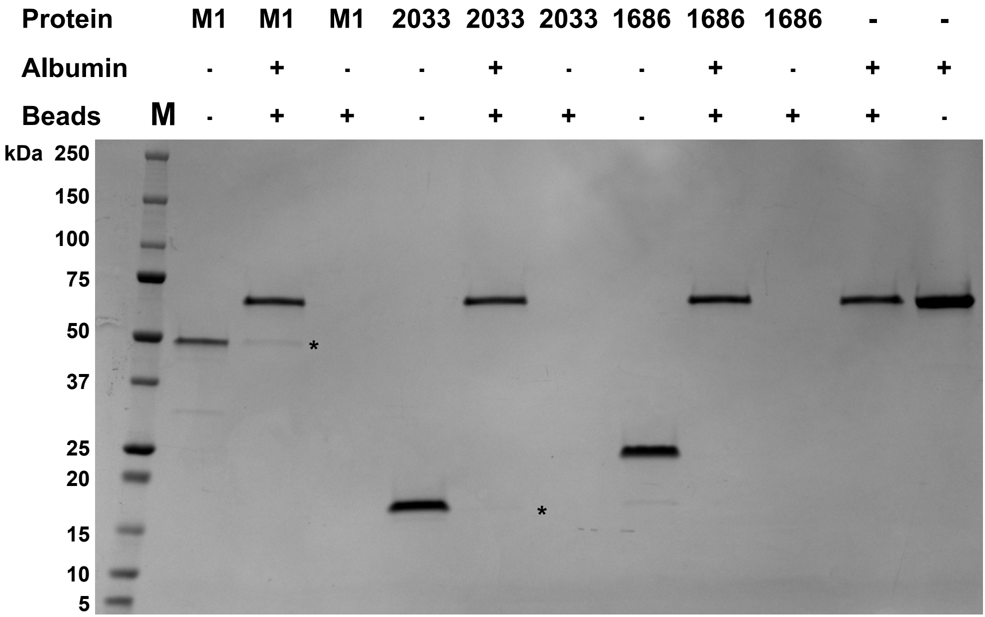

Supplementary Figure 5. Reverse affinity-capture using His-tagged human serum albumin and the streptococcal prey protein M1, as well as the uncharacterized proteins Q99XU1 (*SPy_2033;* labeled here 2033 for short) and Q99YI6 (*SPy_1686;* labeled here 1686 for short). Naked Ni2+-beads incubated with the respective streptococcal proteins were used as a negative control, and to further demonstrate that no affinity-tag was left intact on these proteins after TEV-protease cleavage, and so affecting the conclusions made here. M indicates markers (Precision Plus Protein Dual Xtra Prestained Protein Standard, 2—250 kDa size range). The asterisks highlight faint bands of M1 and 2033 bound to albumin. The uncropped SDS-PAGE gel is provided in the Source Data file.

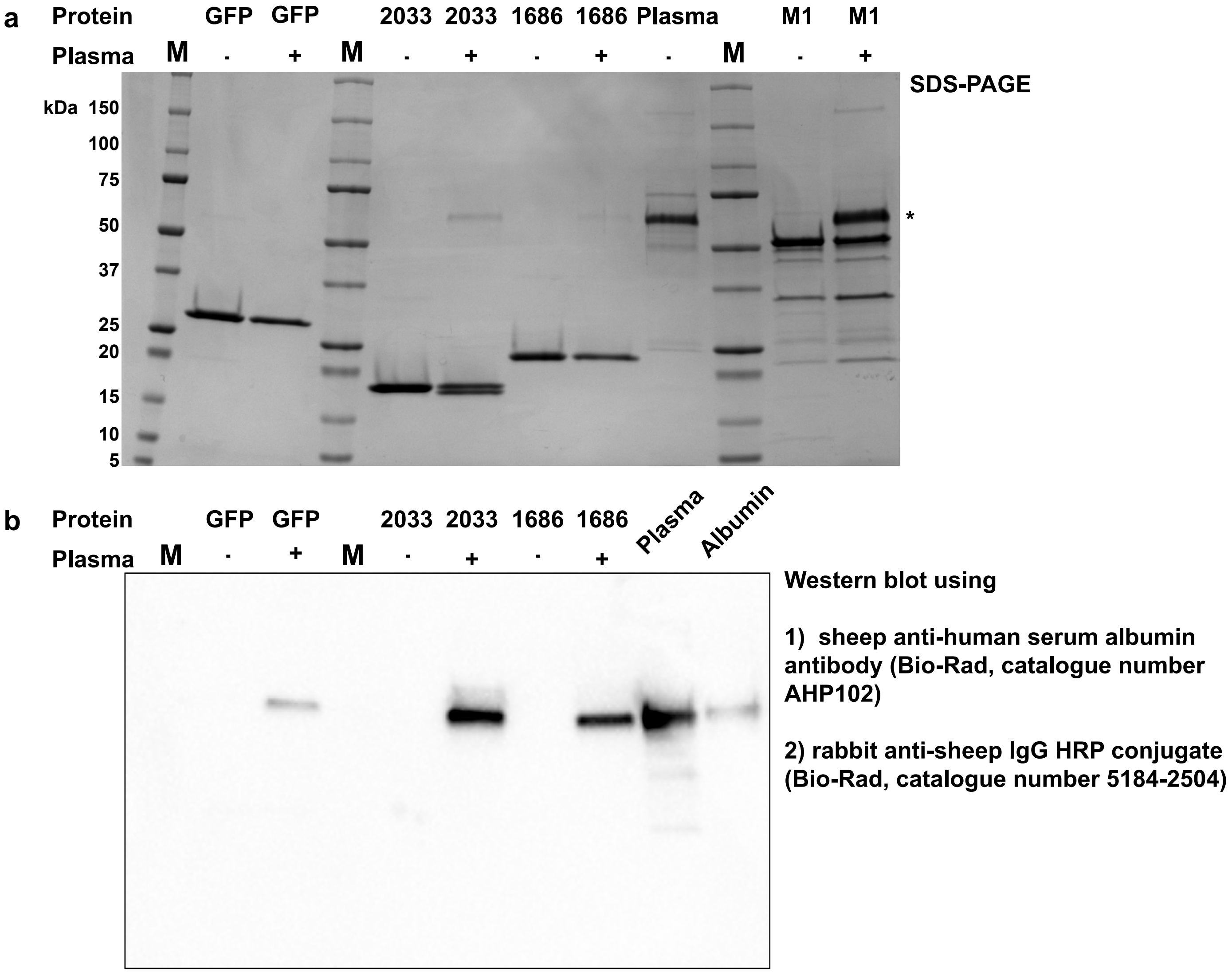

Supplementary Figure 6. Western blot analysis of albumin binding to uncharacterized proteins Q99XU1 (*SPy_2033;* labeled here 2033 for short) and Q99YI6 (*SPy_1686;* labeled here 1686 for short) in AP reactions in human plasma. a) Coomassie blue stained SDS-PAGE gel showing the AP reactions. The interaction between the M1 protein and albumin is clearly visible on the SDS-PAGE gel (indicated with an asterisk) and was thus omitted from the Western blot analysis. sfGFP was used as a negative control. b) Western blot analysis of the AP reactions probing for albumin binding. sfGFP used as a negative control shows a weaker signal for albumin binding as does that of Q99XU1 (*SPy_2033*) and Q99YI6 (*SPy_1686*). In a) and b), M indicates markers (Precision Plus Protein Dual Xtra Prestained Protein Standard, 2–250 kDa size range); Albumin, His-tagged albumin (Acro Biosystems), 0.1 ug; and Plasma, pooled human plasma used in the AP reactions, 1 ug. The uncropped SDS-PAGE gel and western blot are provided in the Source Data file.

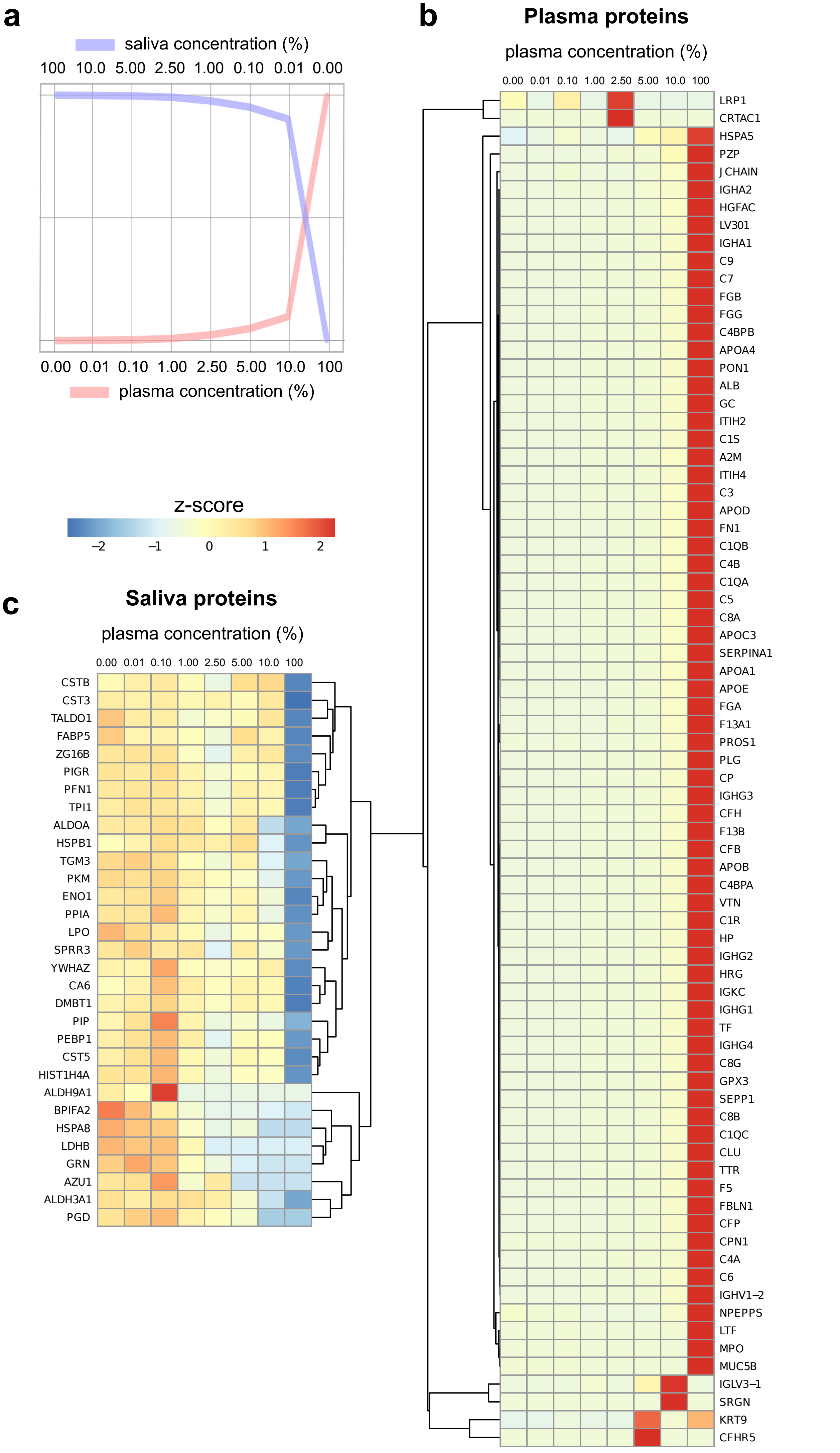

Supplementary Figure 7. Plasma dilutions in saliva. a) Different mixtures of plasma in saliva (0.01%, 0.1%, 1%, 2.5%, 5% and 10% plasma) were used to study the effect of the host environment on protein-protein interactions. b-c) Cluster analysis of the different mixtures of plasma in saliva, with division into typical plasma and saliva proteins based on z-score. Source data are provided as a Source Data file.

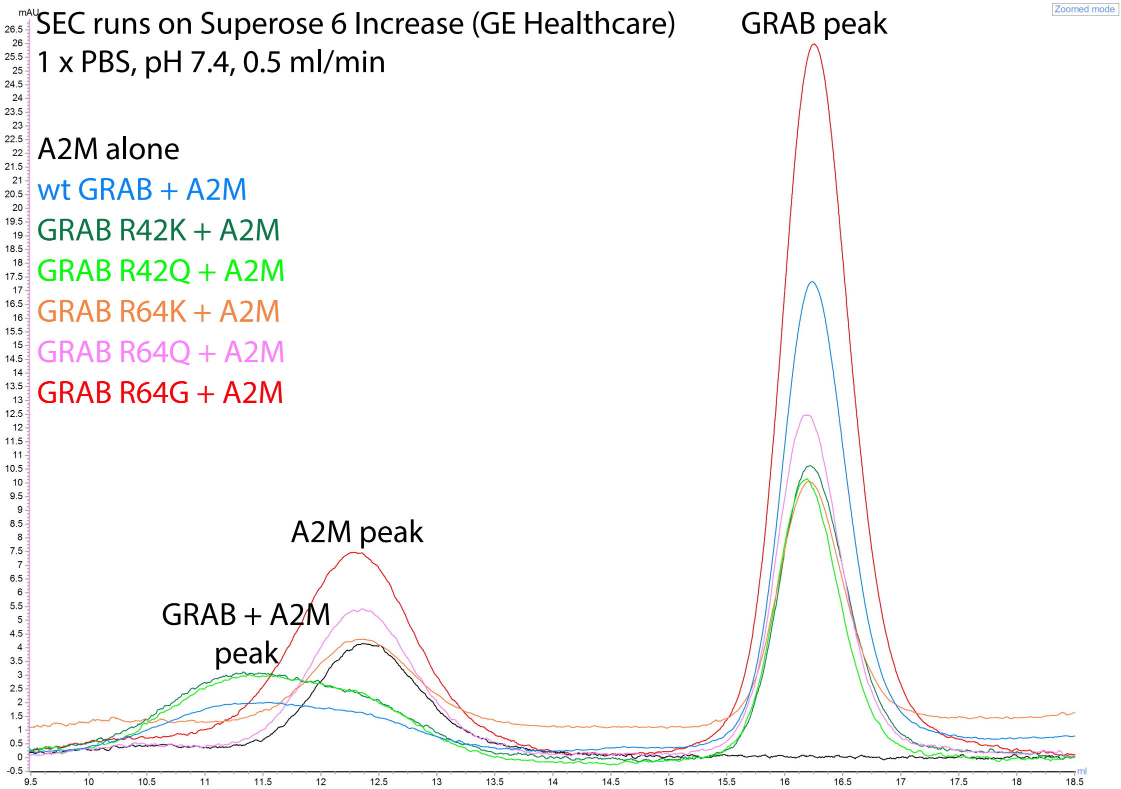

Supplementary Figure 8. Size exclusion chromatography of wildtype GRAB and point mutants together with human A2M. As is evident based on co-elution of the two proteins, the wildtype (wt) GRAB and the R42 mutant bind A2M, whereas the R64 mutant does not. These findings are in line with previous reports showing that R64 is crucial for the interaction with A2M whereas a mutation on R42 has a lesser effect [4].

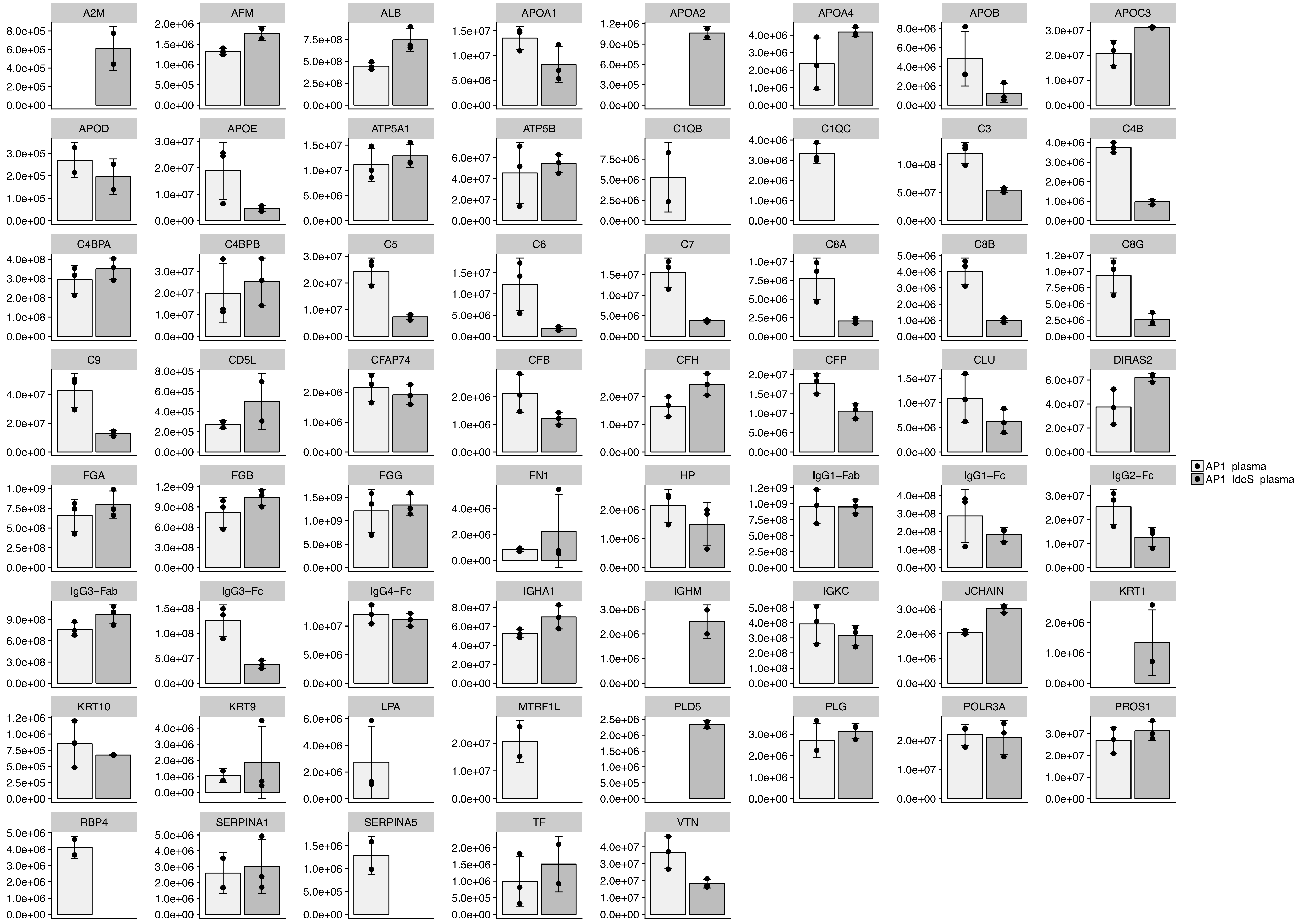

Supplementary Figure 9. SA-DIA using the M1 serotype AP1 strain in pooled human plasma and plasma pre-digested with IdeS. Proteins identified via DIA-MS are organized alphabetically with relative intensities indicated on the y-axis. The data was filtered using a log2 fold enrichment of >2 and P value < 0.001 calculated using the Student’s t-test. The experiment was prepared using n=3 biologically independent samples. Source data are provided as a Source Data file.

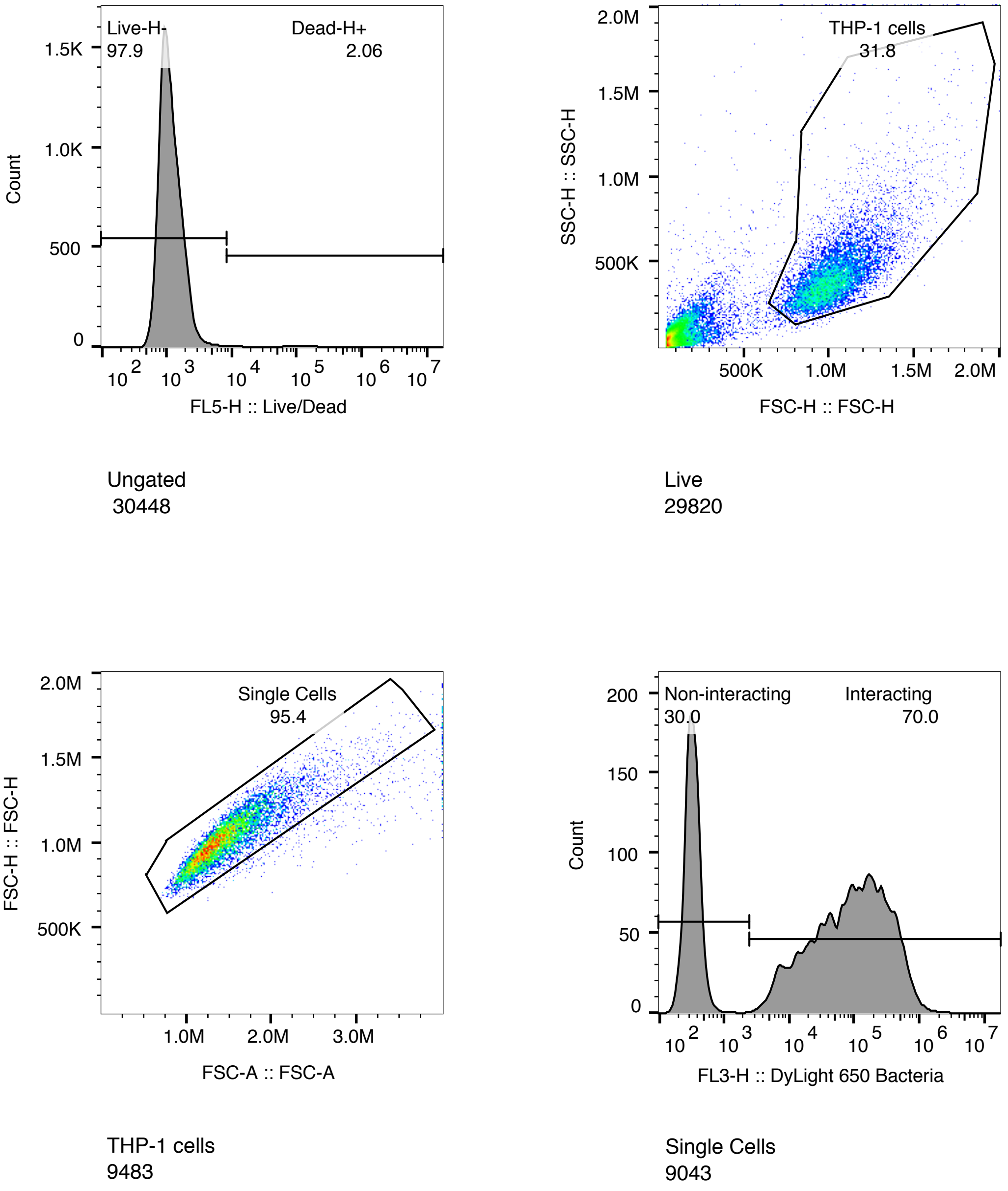

Supplementary Figure 10. Gating strategy for flow cytometry data relating to Figure 7i. Dead cells were excluded after testing positive with the LIVE/DEAD™ Fixable Violet Dead Cell Stain Kit at 405 nm excitation (ThermoFisher). Live cells were gated on forward and side scatter, followed by excluding doublets by gating on FSC-H versus FSC-A. Interaction was defined by cells positive for Dylight 650, and internalization by cells positive for Dylight 650 and negative for Dylight 488. Boundaries between positive and negative were defined using unstained and stained controls.

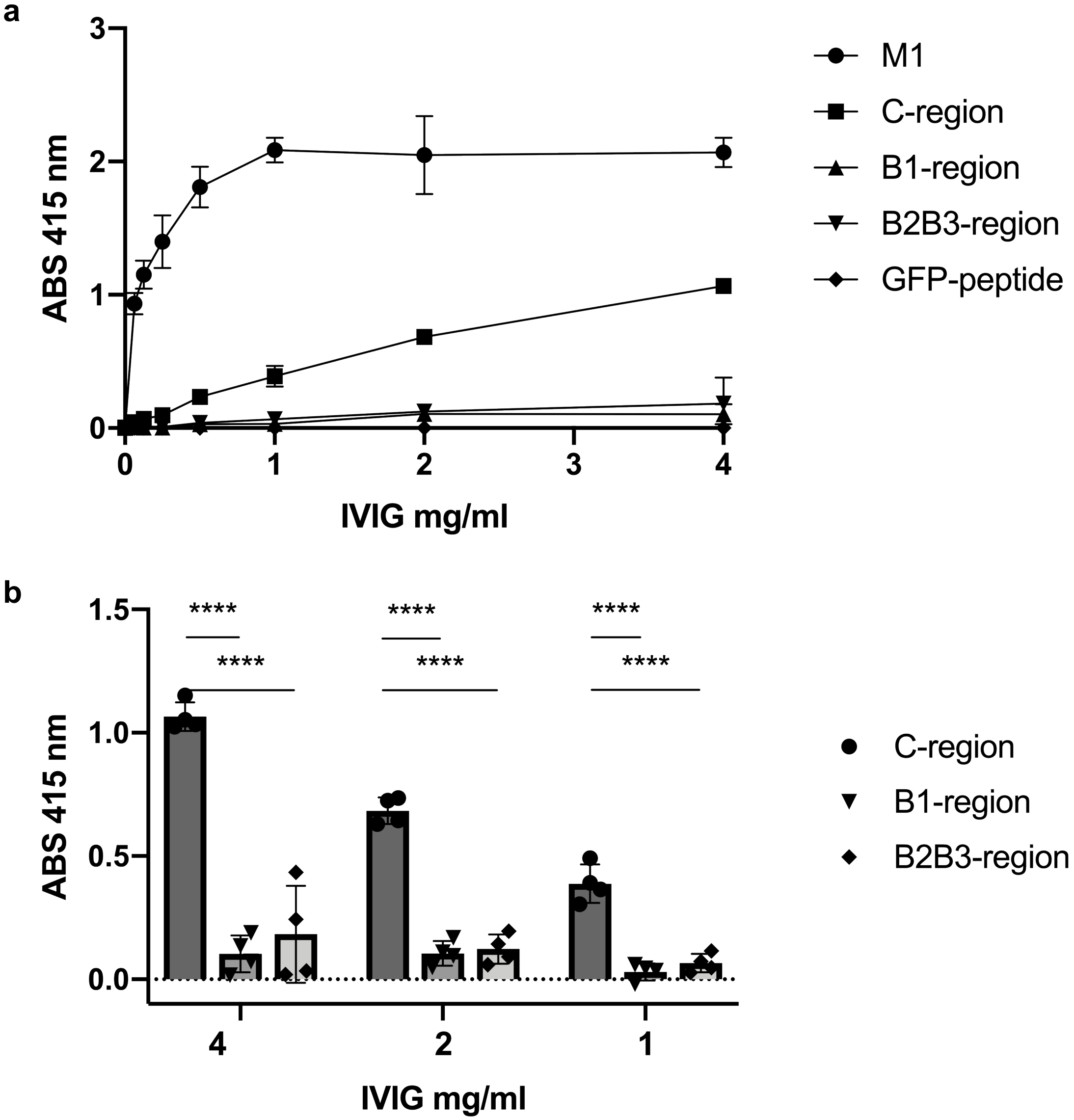

Supplementary Figure 11. ELISA comparing the binding of pooled human intravenous immunoglobulin (IVIG) to different M1 protein derived synthetic peptides. a) The number of pooled immunoglobulins bound to the full-length M1 protein and the derived peptides based on their ELISA absorbance at 415 nm. The M1 protein was used as a positive control, and a GFP derived peptide as a negative control. The absorbance values of the GFP signal were subtracted from the data prior to analysis. b) Two-way ANOVA of the peptide data indicates that there is a significant difference (four asterisks $P < 0.0001$ using the Tukey's t-test and n=4 biologically independent samples) in the binding of immunoglobulins present in IVIG to the C-region of M1 protein containing the J8-peptide, as opposed to B1- and B2B3-region derived peptides of M1 protein. Error bars are expressed as standard deviation (s.d.) from the mean. Source data are provided as a Source Data file.

Supplementary Table 1: A list of synthetic peptides used in this study

A list of the synthetic peptides used for the enzyme-linked immunosorbent assay (ELISA) presented in Supplementary Figure 11.

Peptide name	Peptide sequence
B1-region	LEKELEEKKEALELAIDQASRDYHRATAWSHPQFEK
B2B3-region	LEKELEEKKKALELAIDQASQDYNRANVLEKELEWSHPQFEK
C-region	ELDKVKEEKQISDASRQGLRRDLDA**SREAKKQVEKAL**EEAWSHPQFEK
GFP (negative control)	TYKTRAEVKFEWSHPQFEK

SUPPLEMENTARY REFERENCES

1. Collins, B. C. *et al.* Quantifying protein interaction dynamics by SWATH mass spectrometry: application to the 14-3-3 system. *Nat. Methods* **10,** 1246–1253 (2013).
2. Szklarczyk, D. *et al.* STRING v10: protein-protein interaction networks, integrated over the tree of life. *Nucleic Acids Res.* **43,** D447–52 (2015).
3. Rasmussen, M., Müller, H. P. & Björck, L. Protein GRAB of *Streptococcus pyogenes* regulates proteolysis at the bacterial surface by binding alpha2-macroglobulin. *J. Biol. Chem.* **274,** 15336–15344 (1999).
4. Godehardt, A. W., Hammerschmidt, S., Frank, R. & Chhatwal, G. S. Binding of α2-macroglobulin to GRAB (Protein G-related α2-macroglobulin-binding protein), an important virulence factor of group A streptococci, is mediated by two charged motifs in the ΔA region. *Biochem. J.* **381,** 877–885 (2004).
