# Supplementary material for: A quantitative Streptococcus pyogenes–human protein–protein interaction map reveals localization of opsonizing antibodies
Source: Nat Commun. 2019 Jun 21;10:2727. doi: 10.1038/s41467-019-10583-5 (PMC6588558; doi:10.1038/s41467-019-10583-5)
Supplement: Supplementary file 2 — Description of Additional Supplementary Files [file 41467_2019_10583_MOESM2_ESM.docx]

**Description of Additional Supplementary Files**

**File Name: Supplementary Data 1**

**Description:** Bait proteins used in this study Protein names, Uniprot IDs, gene names, SPy-numbers, construct lengths (amino acids), location of affinity tag (N- or C-terminal), reference to PDB structures (when relevant) and sequence coverage of the expressed constructs as determined by data-dependent acquisition (DDA) liquid chromatography tandem mass spectrometry (LC-MS/MS) are given.

**File Name: Supplementary Data 2**

**Description:** Purity of the bait proteins used in this study as determined by DDA LC-MS/MS analysis Each bait protein was analyzed via DDA LC-MS/MS and searched against an in-house compiled database containing the Homo sapiens and Streptococcus pyogenes serotype M1 reference proteomes (UniProt proteome IDs UP000005640 and UP000000750, respectively), with the S. pyogenes Protein H added (UniProt ID P50470), as described in the Methods section.

**File Name: Supplementary Data 3**

**Description:** AP-DIA interactome For each bait protein, its Uniprot ID (Bait_Uniprot), bait gene name (Bait_GeneName), captured prey Uniprot ID (Prey_Uniprot), prey gene name (Prey_GeneName), condition (COND; plasma or saliva), mean normalized abundance (normalized_abundance_mean), standard deviation of normalized abundance (normalized_abundance_sd), log intensity (log_int), bait normalized mean (bait_norm_mean), bait normalized log mean (bait_norm_mean_log), standard deviation of normalized bait (bait_norm_sd), fold change to sfGFP (fold_change), log2 fold change to sfGFP (fold_change_log2), control pvalue (pvalue_control), log pvalue (log_pval), maximum peptide count (max_pep_count), observed in number of replicates (n_replicates_obs), total number of replicates (n_replicates_total), interaction type (Type; contaminant or interactor) and bait-bait interaction is given.

**File Name: Supplementary Data 4**

**Description:** A table summarizing the TX-MS analysis For each streptococcal – human protein-protein pair, the bait Uniprot ID, bait gene name, bait PDB ID (when relevant), host Uniprot ID, host gene name, host PDB ID, total number of crosslinks per given protein-protein interaction, number of high-quality crosslinks per given protein-protein interaction and a reference to an example spectrum in Supplementary Data 1 is given.

**File Name: Supplementary Data 5**

**Description:** A list of crosslinked peptide pairs For each streptococcal – human protein-protein pair, the bait Uniprot ID, the bait gene name, the host Uniprot ID, the host gene name and the crosslinked peptides is given. The number in parenthesis at the end of each peptide sequence indicates the crosslinked lysine residue number.

**File Name: Supplementary Data 6**

**Description:** Example DDA peptide spectra of interactions identified via TX-MS in SA-DIA samples. One spectrum is shown for each streptococcal-host protein-protein pair. The red and the blue peptides represent streptococcal and host derived peptides, respectively, whereas the green signal arises from the aforementioned peptides containing an additional DSS crosslinker arm. See also Supplementary Tables 4 and 5 for details.

**File Name: Supplementary Data 7**

**Description:** DNA sequences for the synthetic ORFs expressed in this study.
