# Supplementary material for: A quantitative Streptococcus pyogenes–human protein–protein interaction map reveals localization of opsonizing antibodies
Source: Nat Commun. 2019 Jun 21;10:2727. doi: 10.1038/s41467-019-10583-5 (PMC6588558; doi:10.1038/s41467-019-10583-5)
Supplement: Supplementary file 8 — Supplementary Data 6 [file 41467_2019_10583_MOESM8_ESM.pdf]

1a: csrR - C1R

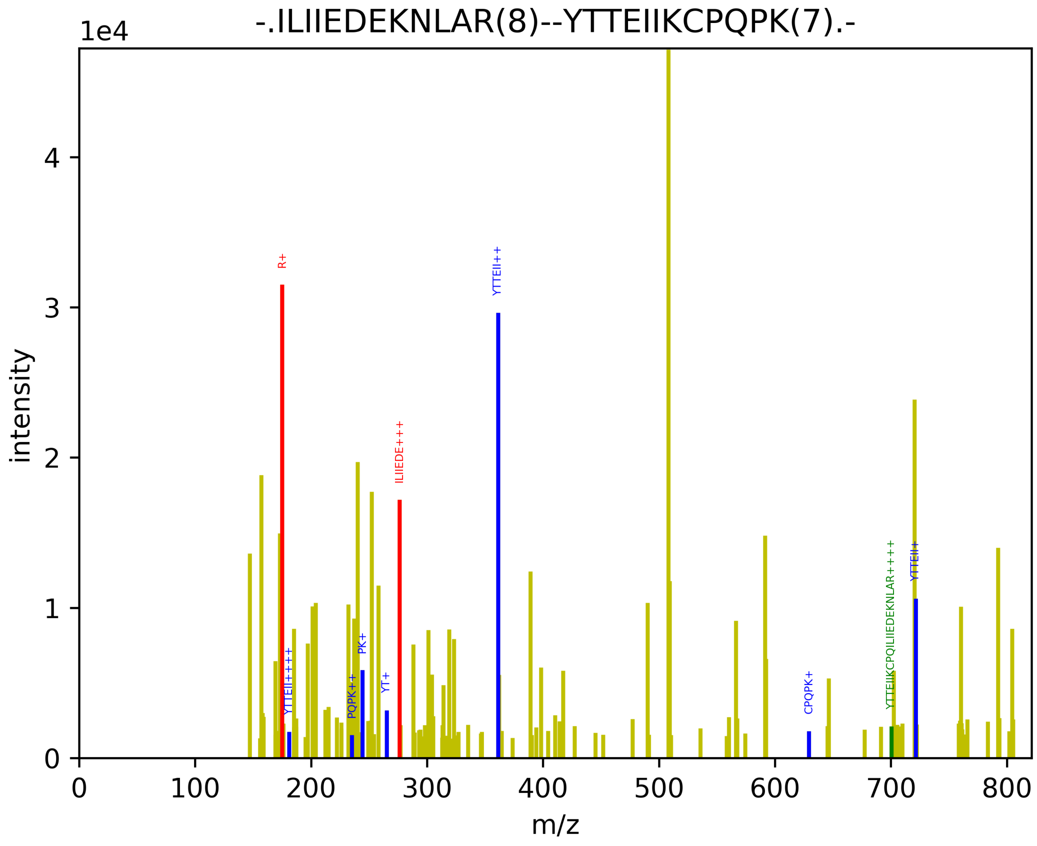

1b: csrR - C5

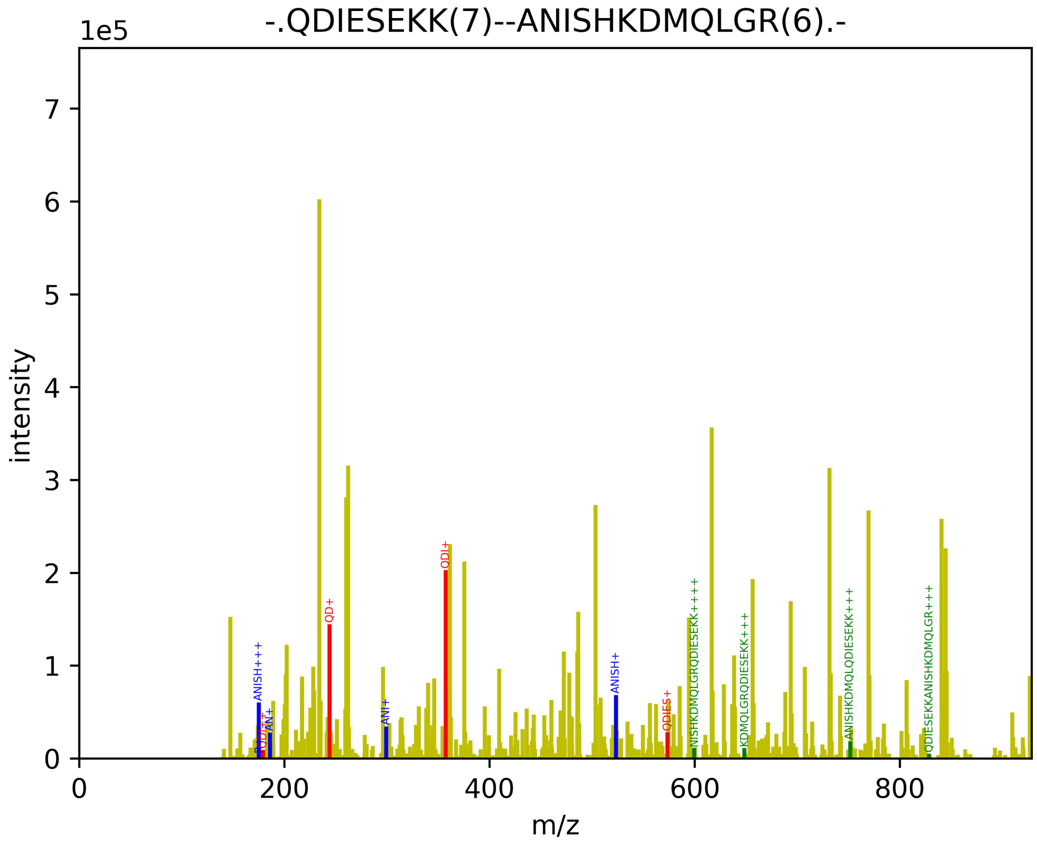

Supplementary Data 6: Example DDA peptide spectra of interactions identified via TX-MS in SA-DIA samples. One spectrum is shown for each streptococcal-host protein-protein pair. The red and the blue peptides represent streptococcal and host derived peptides, respectively, whereas the green signal arises from the aforementioned peptides containing an additional DSS crosslinker arm. See also Supplementary Tables 4 and 5 for details.

1c: csrR - FGA

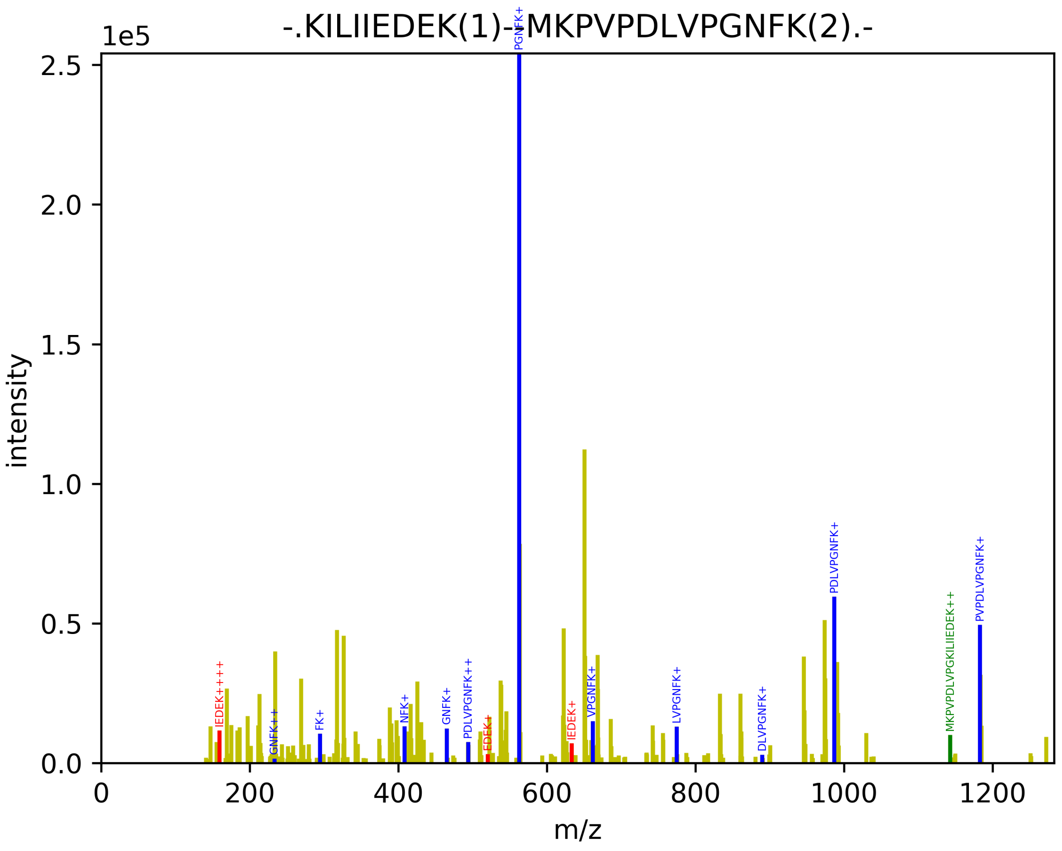

1d: csrR - FGG

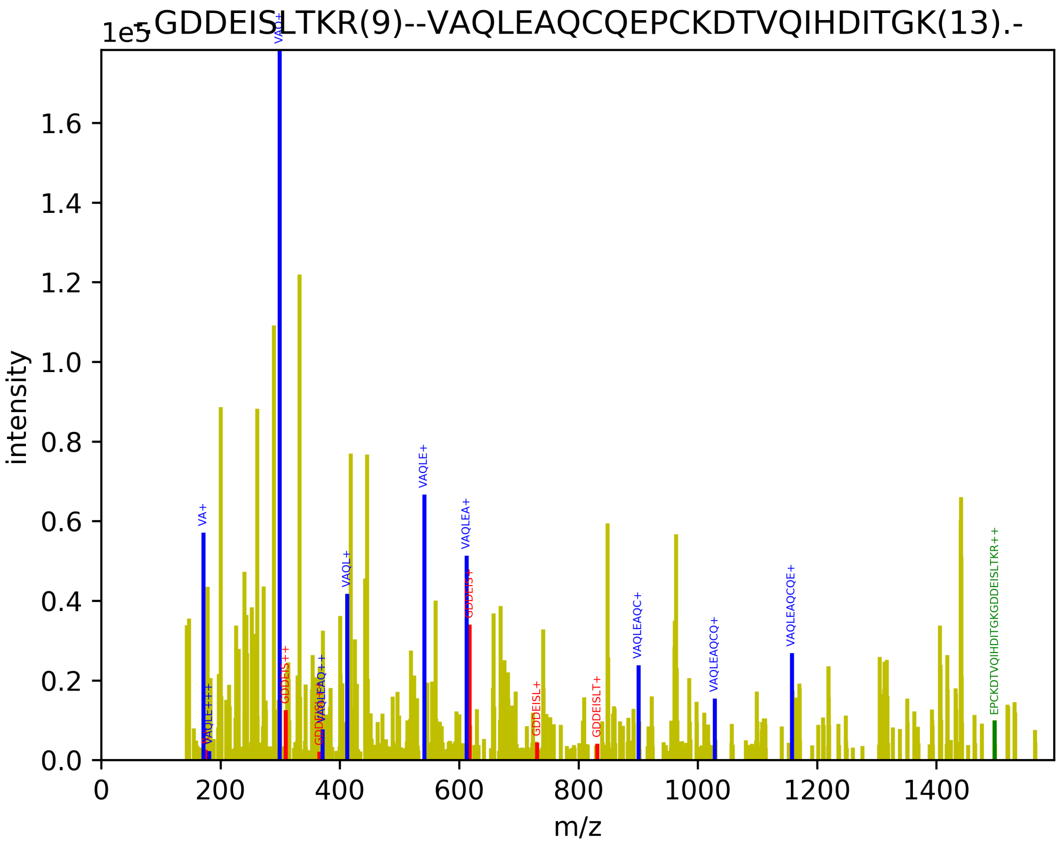

1e: csrR - FN1

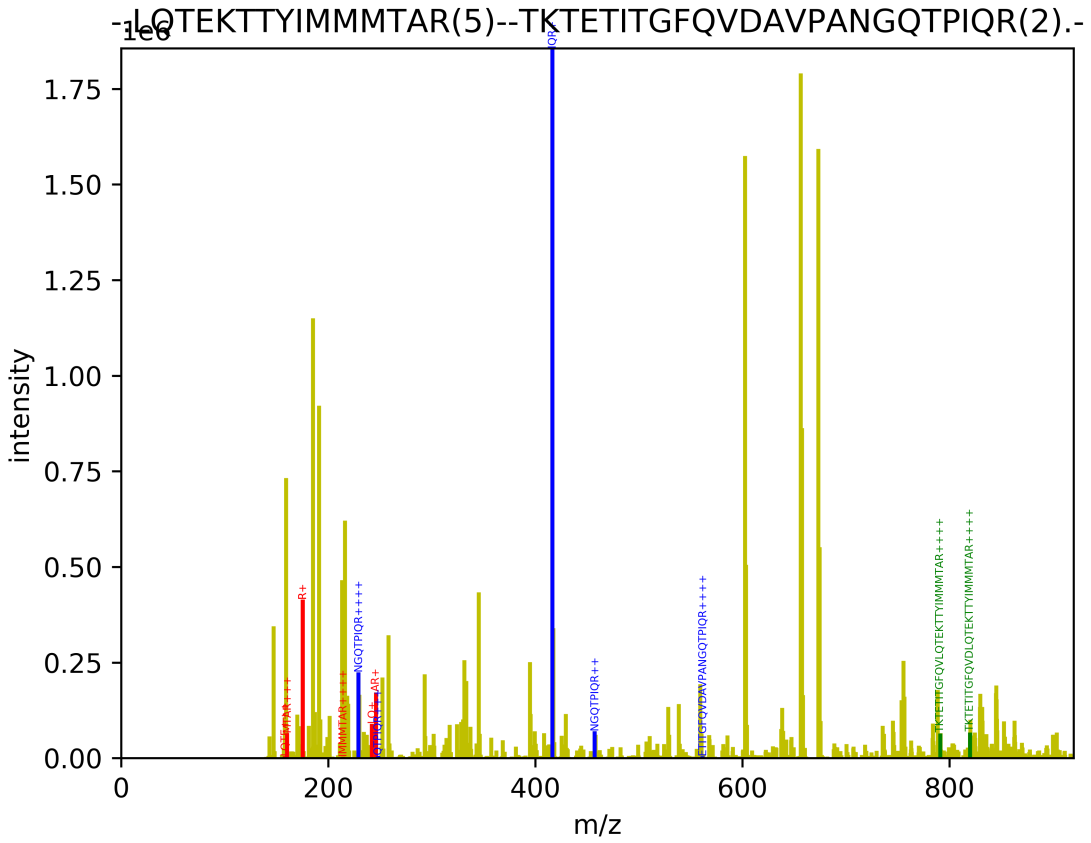

1f: csrR - C4B

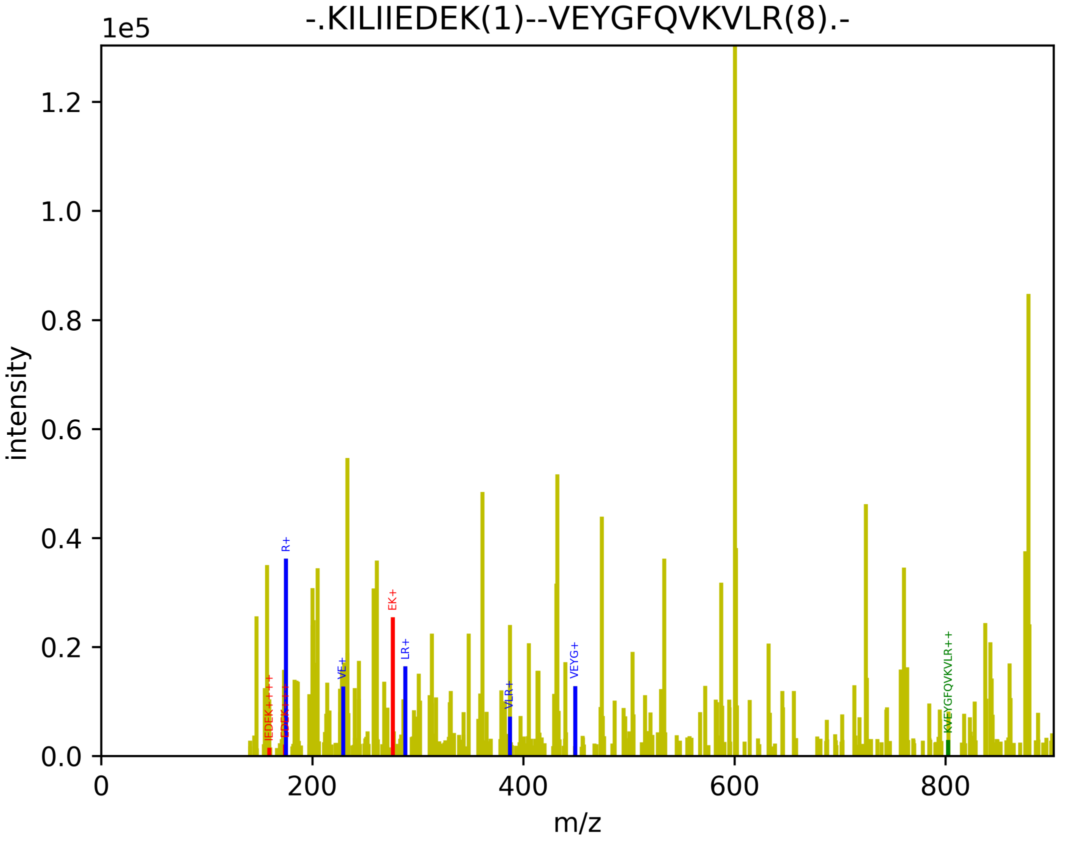

1g: csrR - CFHR5

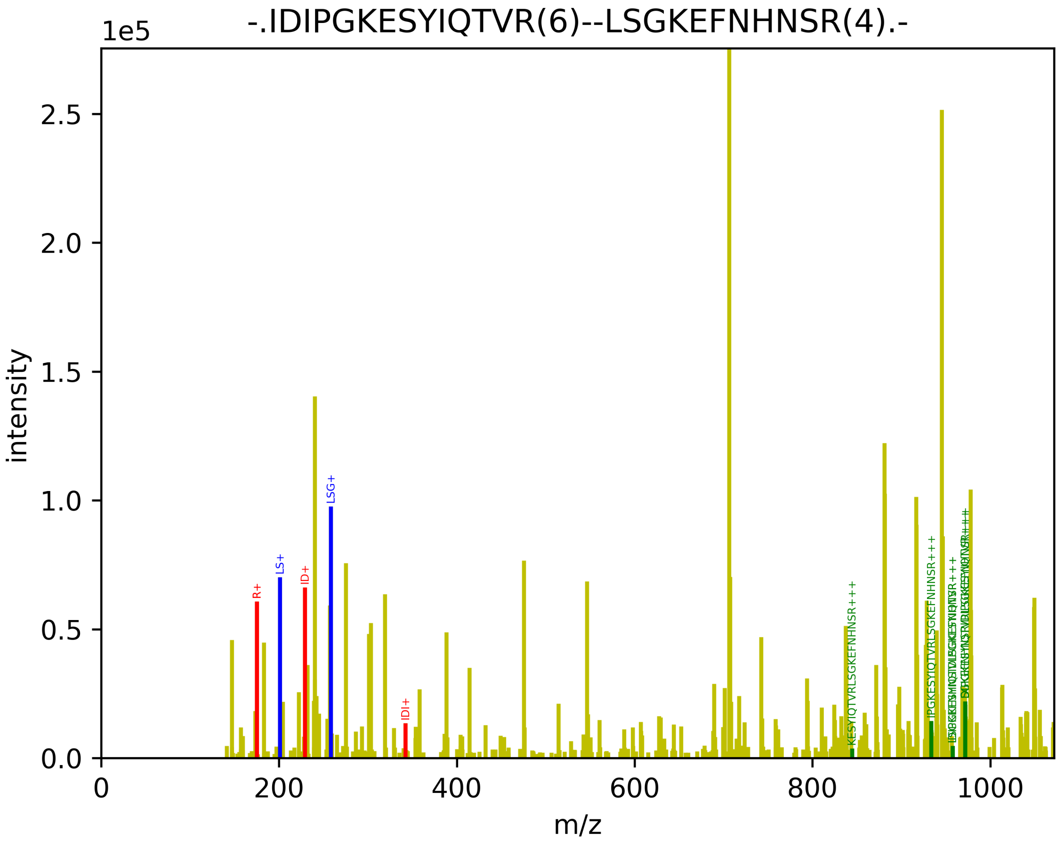

2a: prsA1 - APOE

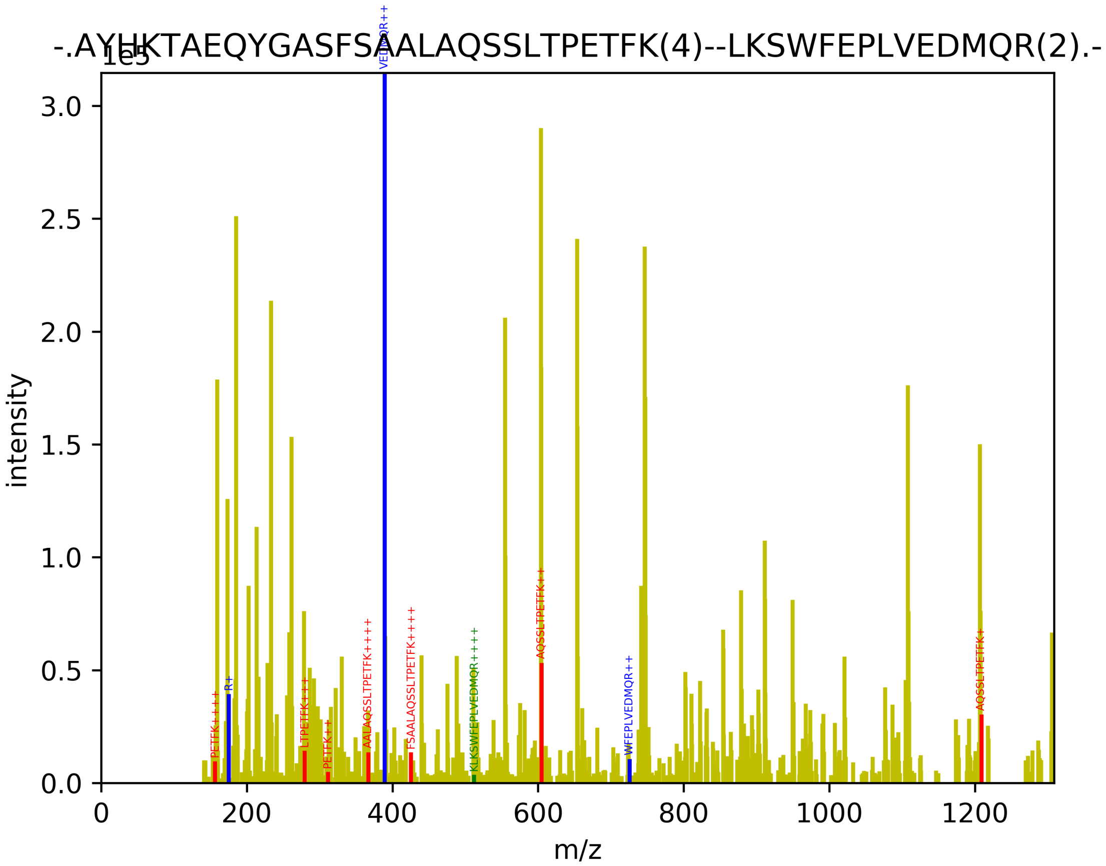

2b: prsA1 - PROS1

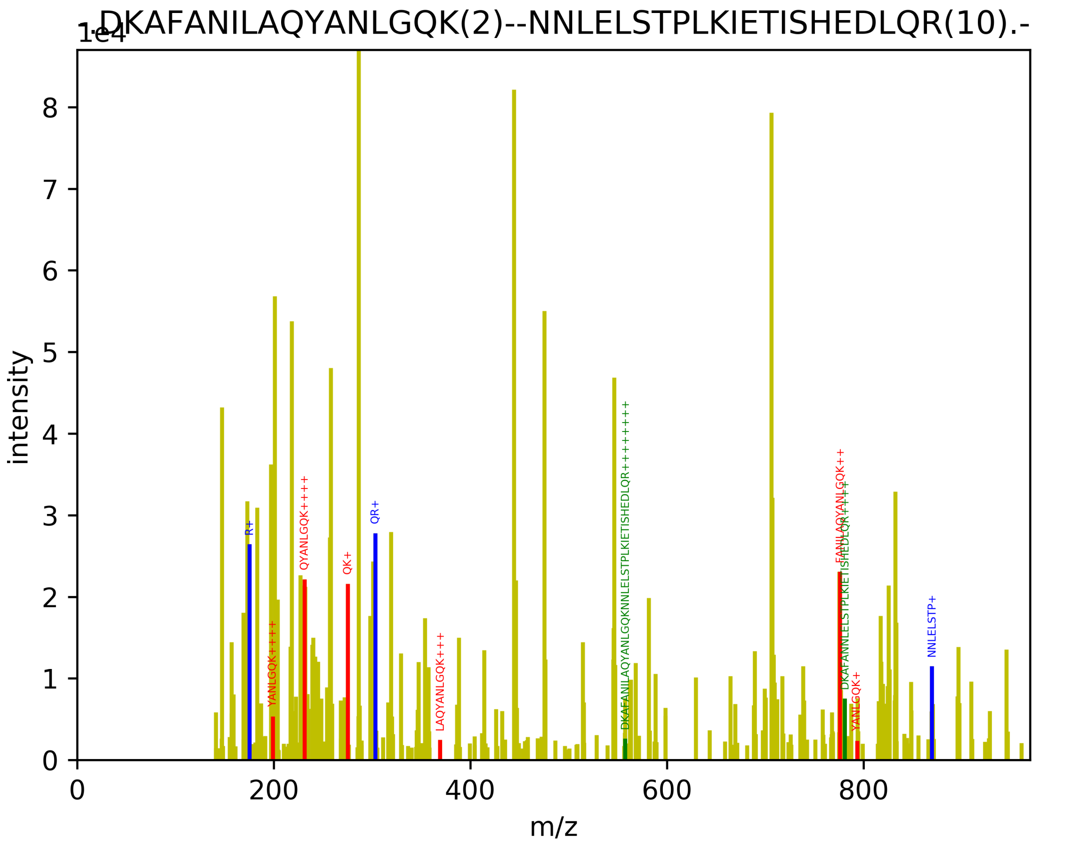

3a: ISP - A2M

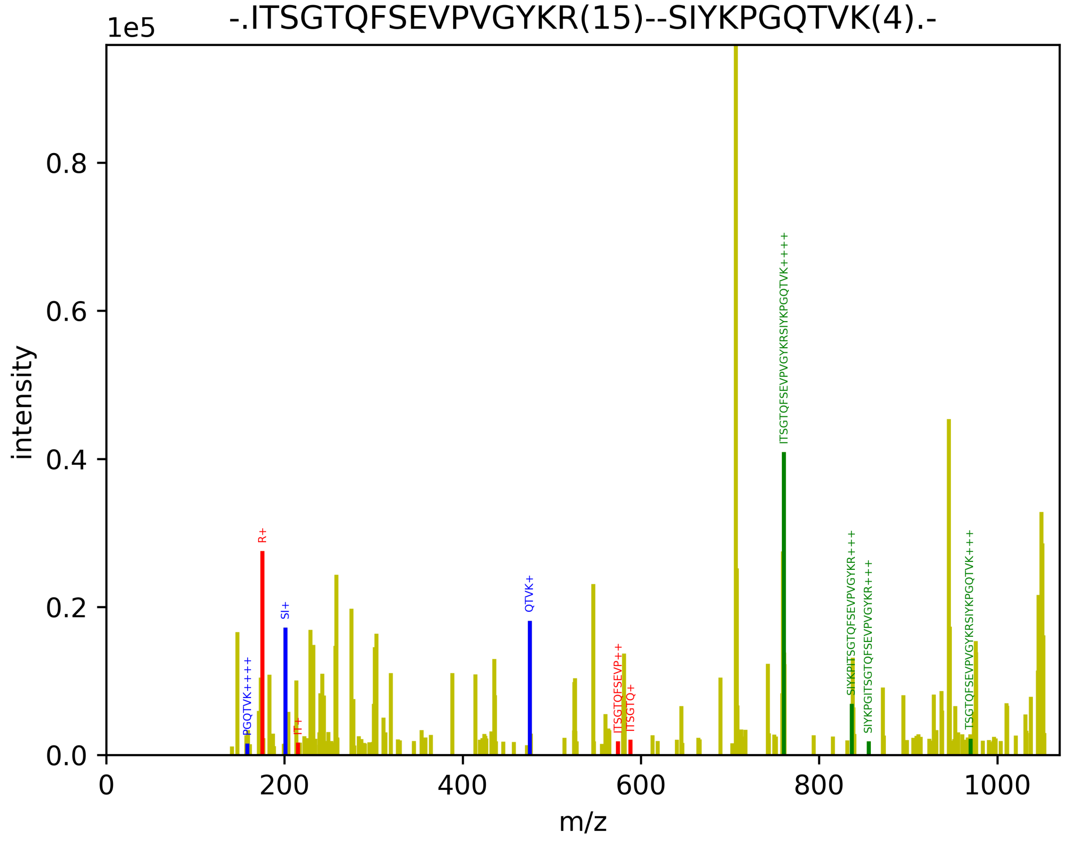

3b: ISP - C5

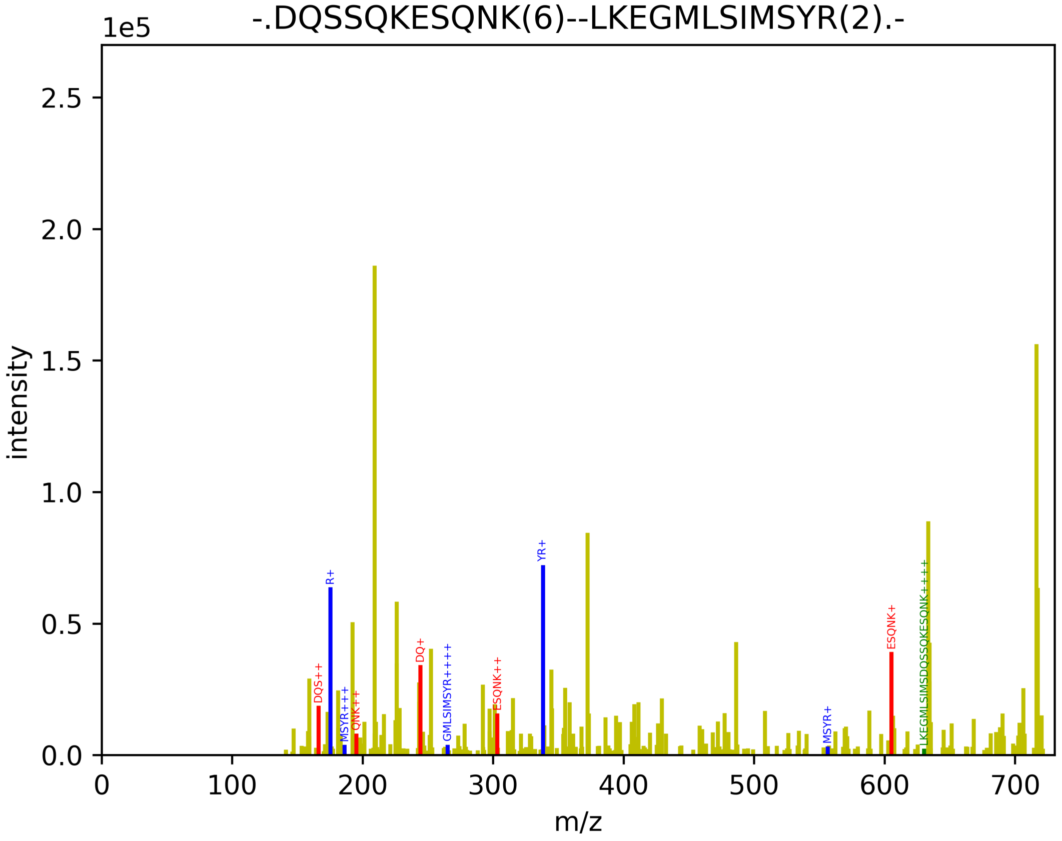

3c: ISP - FGA

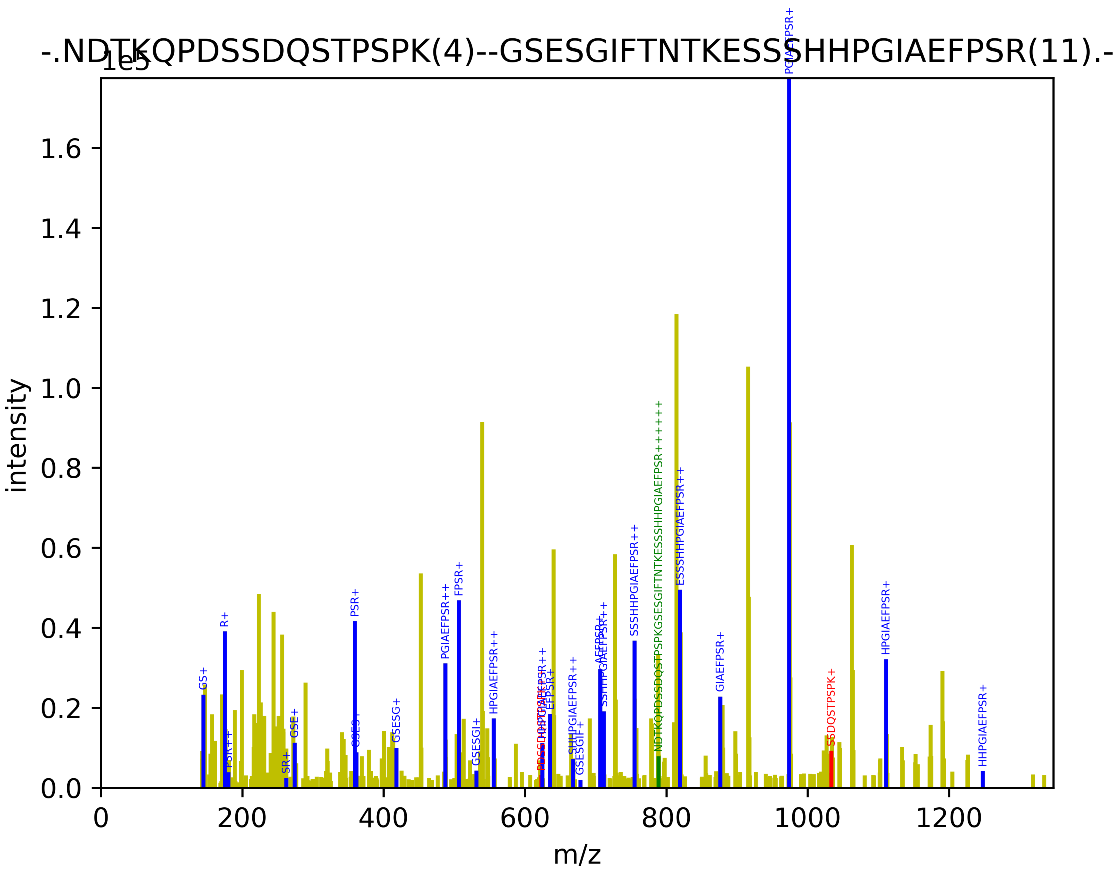

3d: ISP - FGG

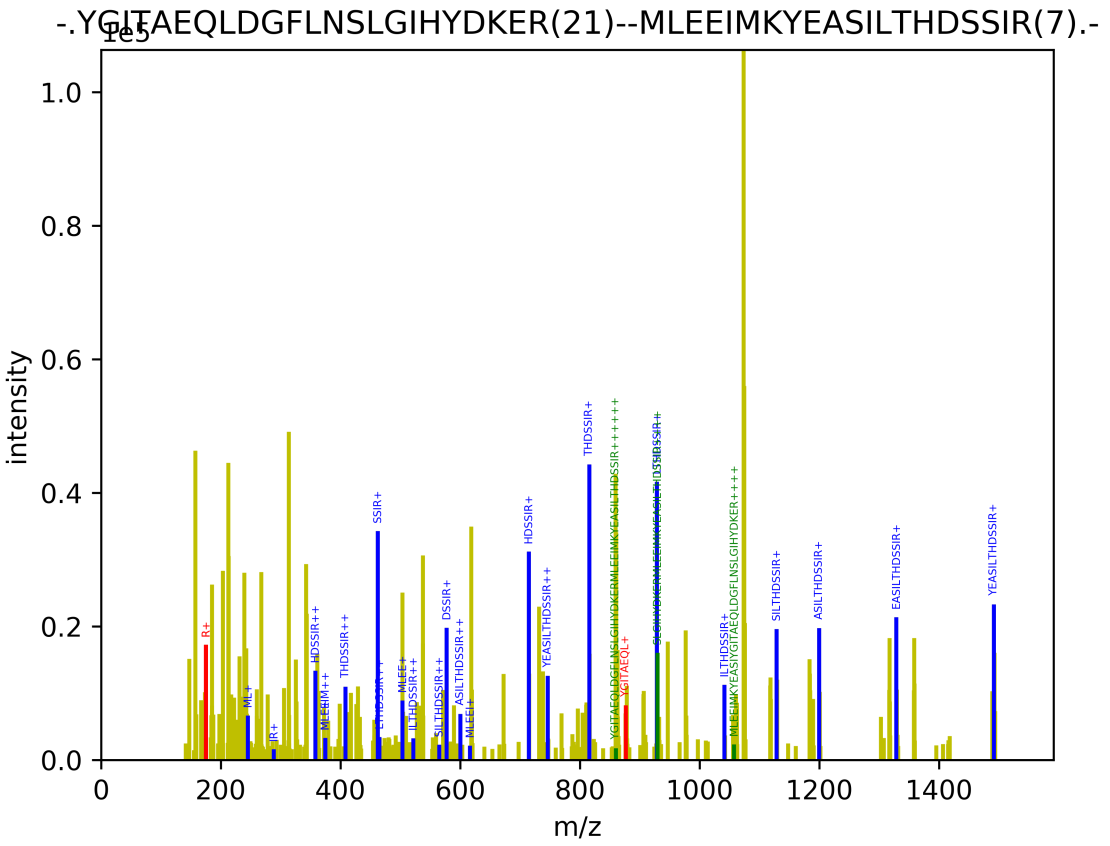

3e: ISP - FN1

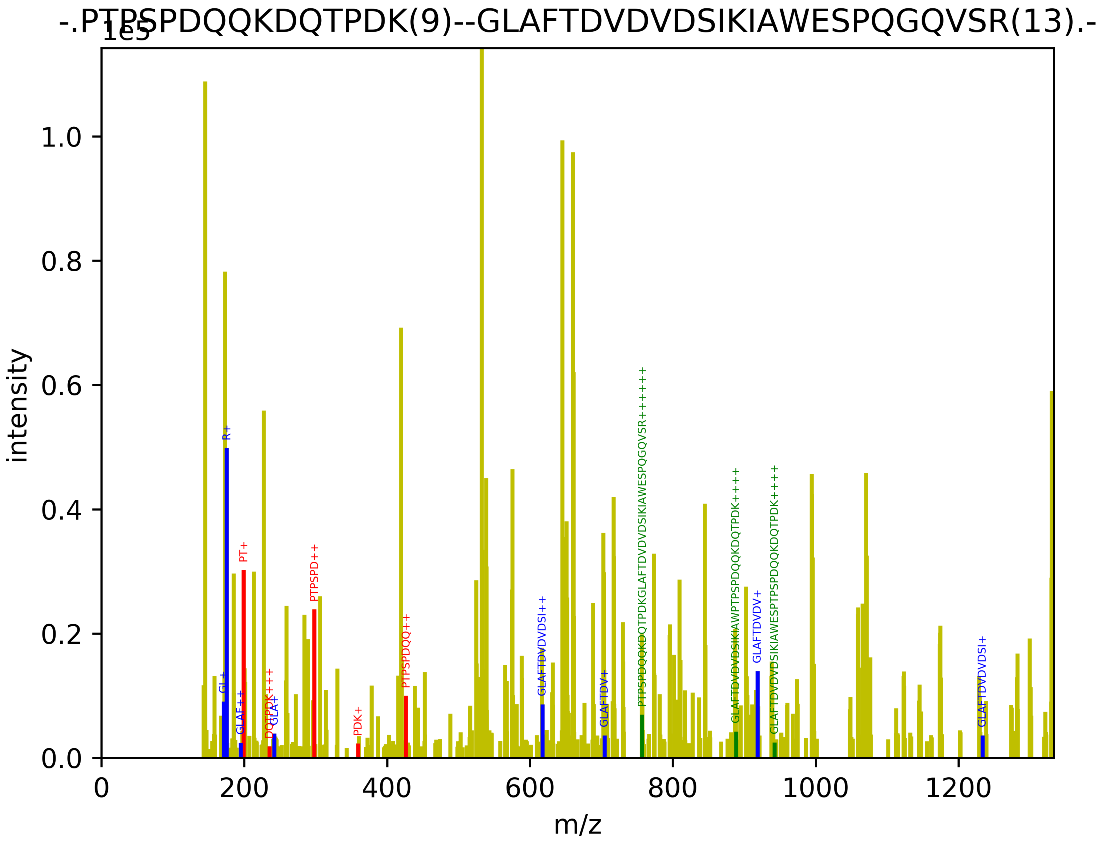

3f: ISP - PROS1

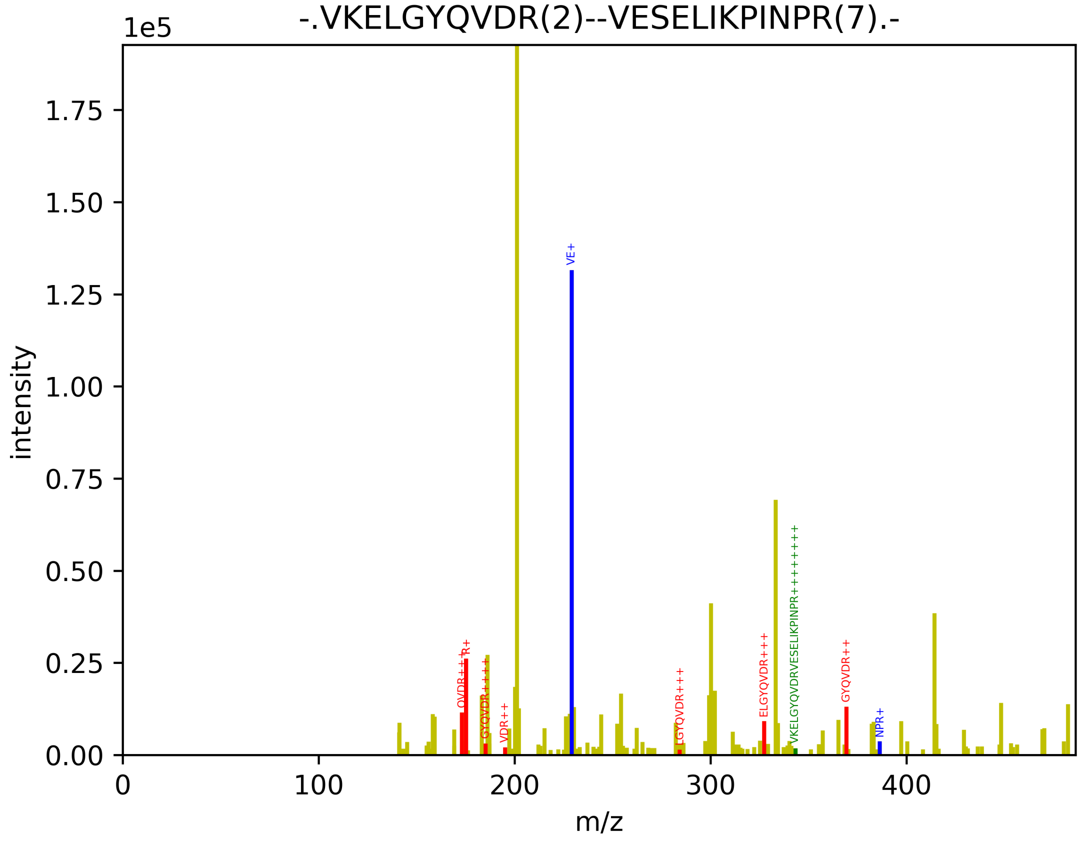

3g: ISP - C7

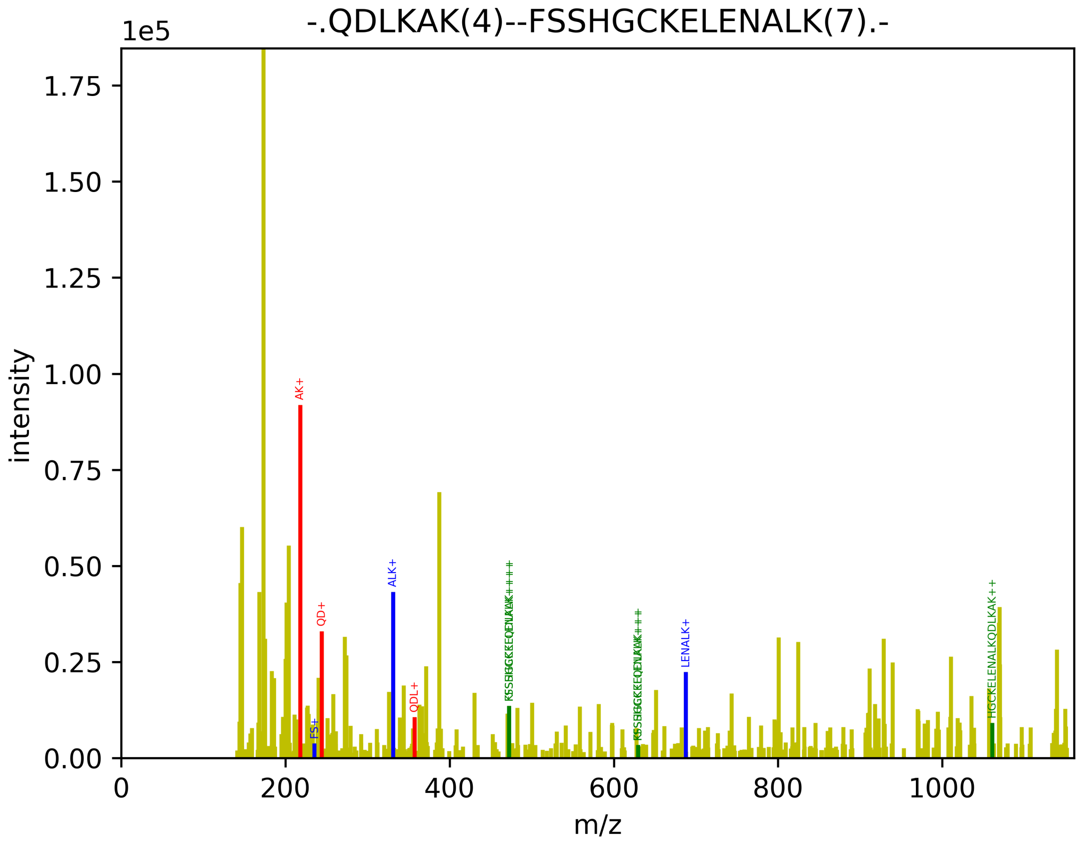

3h: ISP - CLU

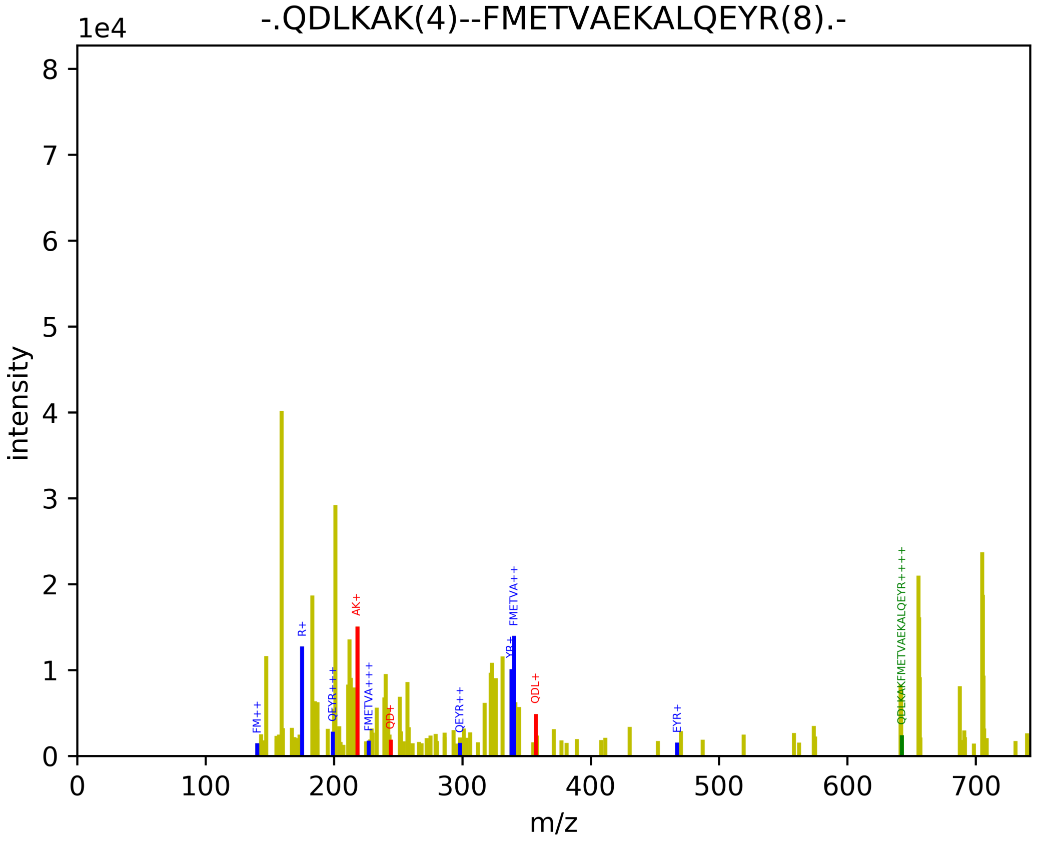

3i: ISP - C6

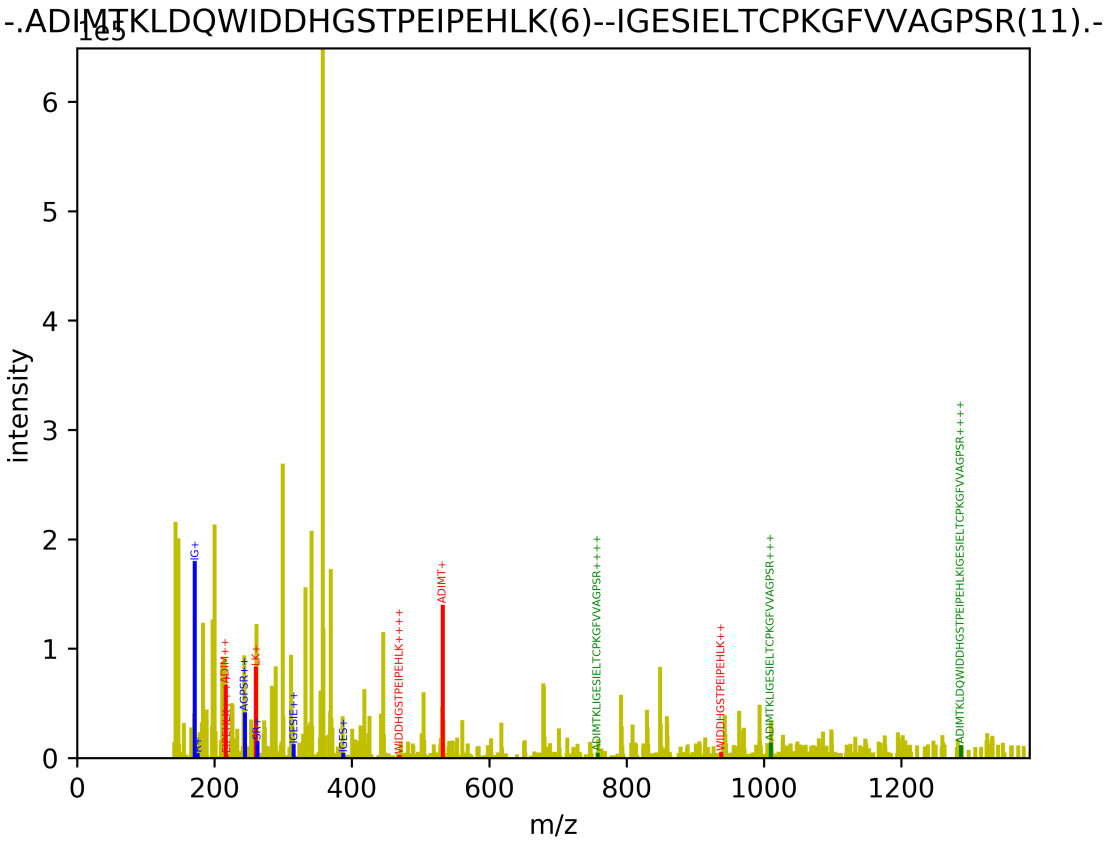

-.ADIMTKLDQWIDDHGSTPEIPEHLK(6)--IGESIELTCPKGFVVAGPSR(11).-
1e5
intensity
m/z
IG+
ADIM++
AGPSR++
LK+
IGESIE++
IGES+
WIDDHGSTPEIPEHLK++++
ADIMT+
ADIMTKLIGESIELTCPKGFVVAGPSR++++
WIDDHGSTPEIPEHLK++
ADIMTKLIGESIELTCPKGFVVAGPSR+++
ADIMTKLDQWIDDHGSTPEIPEHLKIGESIELTCPKGFVVAGPSR++++
