# Supplementary material for: A quantitative Streptococcus pyogenes–human protein–protein interaction map reveals localization of opsonizing antibodies
Source: Nat Commun. 2019 Jun 21;10:2727. doi: 10.1038/s41467-019-10583-5 (PMC6588558; doi:10.1038/s41467-019-10583-5)
Supplement: Supplementary file 9 — Supplementary Data 7 [file 41467_2019_10583_MOESM9_ESM.pdf]

SUPPLEMENTARY DATA 7: DNA sequences of synthetic constructs

```
>Uncharacterized_protein_Q9A170_SPy_0433
ATGCACCATCATCATCATCATTACCCATACGACGTCCCAGACTACGCTTGGAGCCATCCG
CAGTTCGAGAAGGAGAACCTGTACTTCCAATCCATGGCGGAGGATGAACAAAGTAGTACA
CAAAAGCCAGTAAAATTTGATTTGGATGGACCTCAACAAAAAATTAAAGATTATAGTGGC
AACACAATCACTCTAGAAGACTTATATGTTGGTAGTAAAGTAGTAAAAATATATATCCCT
CAAGGATGGTGGGTATATCTTTACAGACAATGTGATCATAACAGTAAAGAACGAGGAATT
TTAGCTAGTCCTATTCTCGAAAAAAATATAACAAAAACAGATCCTTATCGTCAATATTAT
ACAGGAGTACCTTATATTCTTAACTTAGGAGAAGATCCTTTGAAGAAAGGAGAAAAATTA
ACTTTCTCATTTAAAGGAGAAGACGGATTTTATGTCGGTAGCTATATCTATAGAGACTCT
GATACTATAAAAAAAGAAAAAGAAGCTGAAGAAGCACTTCAAAAAAAGGAAGAGGAAAAG
CAACAAAAACAGCTAGAAGAAAGCATGCTAAAGCAGATAAGAGAAGAAGACCATAAACCT
TGGCATCAGCGGTTAAGTGAGAGCATCCAAGATCAGTGGTGGAACTTTAAGGGACTGTTT
CAG

>Uncharacterized_protein_Q99YH8_SPy_1697
ATGCACCATCATCATCATCATTACCCATACGACGTCCCAGACTACGCTTGGAGCCATCCG
CAGTTCGAGAAGGAGAACCTGTACTTCCAATCCATGGGATCCCACCCTCAAACGCAAGAT
AAGGTAGCTAAACATTCTAAAAGTGCTGCTAGTTTACTAAAAAAAGCAGTTAAAGCAGTT
AATGACGCTGATCGTTTAGCCACTGCAGCTGCTATCCAAGAAGCCCAGAAAGCTGTTGAT
AAATTAGCAGAATCTTCTAAGAAAAAAACGCTACAAGAACAGCTAAACGTTGCCAAAGCA
AAGCAAGAGCAAGAAGATGCAGCTACTCAAGCAGTTAAAGCTGCTGAGGAGACTCTCAAT
CAAAATCTTAAAGATATCGCCCAAAAAGCAGTTAACGACTTAAGTAACAAAGGTAAAAAA
GCCGCTTTACAATCTCGGTTAGACGCTATTTTACCAGCGAAACCCATTATTGATGAATTT
CCAAGACAAAGTGGAGAAATAACAGATAACTCTTACTGGACACCTTTCCCAGGAGACGTC
TCAGATACTTATGATAATTCCCAGTCACCTACTTTAGACCCAAGCTCAGAATCTTCAGCT
TCAGATGTTACACCACAACCTAGTCATCCTGATCCAATTCCACCTCAGACCTCTTCAGAA
CCTTCCGACTCAGGAGACAAGCAATCATCAAAAGAG

>Uncharacterized_protein_Q99XU1_SPy_2033
ATGCACCATCATCATCATCATTACCCATACGACGTCCCAGACTACGCTTGGAGCCATCCG
CAGTTCGAGAAGGAGAACCTGTACTTCCAATCCATGGCTTGTGGCACAAAAAAAGATTCT
AAAAAAGAAGAAGTTAAAGAAATCAAAATGTCTGACATCAAAGACGACGCAGTTAGCAAA
AAAACAAAAGTGGTTGATGGCGAAGAAGTTACAGAATACACCACAAAAGATGGAAACGTG
ATTCAGATTCCTGCAGGAAACGAAGAAGGCATGGAATCAAAAGATGCTGGTGGATCAGGC
GCTCCTGCTAAA

>Uncharacterized_protein_Q99YI6_SPy_1686
ATGCACCATCATCATCATCATTACCCATACGACGTCCCAGACTACGCTTGGAGCCATCCG
CAGTTCGAGAAGGAGAACCTGTACTTCCAATCCATGGATATGGATGATAAAGACGATCAT
ATGGATAATCAACCAAAGACAAGTCAAACTAGCAAGAAAGTTAAACTCTCAGAAGACAAA
GCCAAATCAATTGCGTTAAAAGATGCTTCTGTTACGGAAGCTGATGCTCAAATGCTATCA
GTTACACAAGATAATGAAGATGGCAAAGCCGTGTATGAAATCGAGTTTCAAAACAAAGAT
CAAGAATACAGCTATACCATTGACGCCAACTCAGGGGATATTGTGGAAAAAATCTTCTGAA
CCAATCAATGAC

>Putative_GTP_cyclohydrolase_1_type_2_homologue_P67274_ SPy_0931
ATGCACCATCATCATCATCATTACCCATACGACGTCCCAGACTACGCTTGGAGCCATCCG
CAGTTCGAGAAGGAGAACCTGTACTTCCAATCCATGGCTAAAACCCTAATTGATGCCTAT
GAAGCCTTTTGCCCACTTGATTTATCTATGGAAGGAGACGTTAAAGGTCTTCAAATGGGT
TCATTGGATAAAGACATTCGTAAGGTTATGATTACTTTAGATATCCGAGAGTCAACAGTA
GCAGAAGCCATCAAAAATGAAGTTGATCTCATTATAACTAAGCATGCTCCCATCTTTAAA
CCGCTTAAAGATCTGGTTTCATCCCCTCAGCGTGATATCCTTCTAGATTTGGTCAAGCAT
GACATTTCTGTCTATGTGAGCCATACCAATATTGACATTGTACCAGGTGGGCTAAATGAT
TGGTTTTGTGACCTGCTAGAAATCAAGGAAGCAACTTACTTGTCTGAAACCAAAGAAGGC
TTTGGCATTGGTCGTATTGGAACAGTTAAAGAACAAGCGTTAGAAGAGTTAGCAAGTAAG
GTCAAAAGGGTGTTTGATTTAGATACCGTGCGACTTATCCGTTACGATAAGGAAAACCCT
TTGATTTCAAAGATTGCTATCTGTGGCGGAAGTGGTGGTGAATTTTATCAGGATGCTGTG
CAAAAAGGCGCAGACGTTTATATTACAGGGGATATCTATTACCATACAGCACAGGAGATG
```

CTGACAGAAGGACTATTTGCGGTTGACCCGGGGCATCATATTGAGGTGCTTTTTACGGAG
AAATTAAAGGAGAAACTCCAAGGGTGGAAAGAAGAGAACGGTTGGGATGTCAGCATTATT
TCAAGCAAAGCCTCG

>Ornithine_carbamoyltranferase_catabolic_P0C0D0_SPy_1544
ATGCACCATCATCATCATCATTACCCATACGACGTCCCAGACTACGCTTGGAGCCATCCG
CAGTTCGAGAAGGAGAACCTGTACTTCCAATCCATGACACAAGTATTTCAAGGACGTAGC
TTCCTAGCAGAAAAAGATTTTACACGCGCTGAATTAGAATACCTTATTGATTTTTCAGCA
CATTTGAAAGATTTGAAAAAACGTGGTGTGCCTCATCACTACTTAGAAGGTAAAAACATT
GCCCTCTTGTTTGAAAAAACATCAACTCGTACGCGTGCAGCTTTTACAACAGCAGCCATT
GACCTAGGTGCTCACCCAGAATACCTCGGTGCCAATGACATCCAACTTGGTAAAAAAGAA
TCAACAGAAGACACTGCTAAAGTATTGGGTCGTATGTTTGATGGGATTGAATTCCGTGGC
TTTAGCCAACGCATGGTTGAAGAATTGGCAGAATTCTCAGGTGTGCCAGTTTGGAATGGT
TTGACTGATGAATGGCACCCAACCCAAATGCTTGCTGACTATTTAACAGTGAAAGAAAAC
TTTGGCAAACTTGAAGGCCTTACCCTTGTTTACTGTGGTGACGGGCGTAATAACGTTGCC
AATTCCCTTCTTGTGACTGGTGCTATCCTTGGTGTTAATGTTCATATCTTCTCACCAAAA
GAACTTTTCCCAGAAGAAGAGATTGTGACATTGGCTGAAGGCTATGCTAAAGAAAGTGGC
GCTCGTATCCTTATCACAGAAGATGCTGACGAAGCTGTTAAAGGCGCAGACGTGCTTTAC
ACAGATGTTTGGGTATCAATGGGTGAAGAAGACAAATTCAAAGAACGTGTTGAATTGCTT
CAACCATATCAAGTCAACATGGACCTTGTTCAAAAAGCAGGAAATGACAAGCTTATCTTC
TTGCACTGCTTGCCAGCTTTCCATGACACAAATACCGTTTATGGTAAAGACGTTGCTGAA
AAATTCGGCGTGAAAGAAATGGAAGTCACTGATGAAGTCTTCCGCAGCAAATACGCTCGT
CATTTTGACCAAGCTGAAAACCGCATGCACACTATCAAAGCGGTTATGGCAGCAACCCTT
GGTAACCTCTTTATTCCAAAAGTG

>Mitogenic_exotoxin_Z_Q99XW1_SPy_1998
ATGCACCATCATCATCATCATTACCCATACGACGTCCCAGACTACGCTTGGAGCCATCCG
CAGTTCGAGAAGGAGAACCTGTACTTCCAATCCATGTTAGAAGTAGATAATAATTCCCTT
CTAAGGAATATCTATAGTACGATTGTATATGAATATTCAGATACAGTAATTGATTTTAAA
ACCAGTCATAACTTAGTGACTAAGAAACTTGATGTTAGAGATGCTAGAGATTTTTTTATT
AACTCCGAAATGGATGAATATGCAGCCAATGATTTTAAAGATGGAGATAAAATAGCTATG
TTCTCCGTCCCATTTGATTGGAACTACTTGTCAGAAGGAAAAGTCATAGCATATACCTAT
GGTGGAATGACGCCTTATCAAGAAGAACCAATGTCTAAAAATATCCCTGTTAATTTATGG
ATTAATAGAAAGCAGATTCCTGTCCCCTATAATCAAATATCAACTAACAAAACAACAGTT
ACAGCTCAAGAAATTGATCTAAAGGTTAGAAAATTTTTAATATCACAACATCAATTATAT
TCTTCTGGTTCTAGCTACAAAAGTGGTAAATTAGTTTTTCATACAAATGATAATTCAGAT
AAATATTCTCTCGATCTTTTCTATGTAGGATATAGAGATAAAGAAAGTATTTTTAAAGTA
TACAAAGACAATAAATCTTTCAATATAGATAAAATTGGGCATTTAGATATAGAAATTGAC

>Foldase_protein_PrsA1_P60811_SPy_1390
ATGCACCATCATCATCATCATTACCCATACGACGTCCCAGACTACGCTTGGAGCCATCCG
CAGTTCGAGAAGGAGAACCTGTACTTCCAATCCATGCAGAGCACCAACGATAACACCAAA
GTGATTAGCATGAAAGGCGATACCATTAGCGTGAGCGATTTTTATAACGAAACCAAAAAC
ACCGAAGTGAGCCAGAAAGCGATGCTGAACCTGGTGATTAGCCGCGTGTTTGAAGCGCAG
TATGGCGATAAAGTGAGCAAAAAAGAAGTGGAAAAAGCGTATCATAAAACCGCGGAACAG
TATGGCGCGAGCTTTAGCGCGGCGCTGGCGCAGAGCAGCCTGACCCCGGAAACCTTTAAA
CGCCAGATTCGCAGCAGCAAACTGGTGGAATATGCGGTGAAAGAAGCGGCGAAAAAAGAA
CTGACCACCCAGGAATATAAAAAAGCGTATGAAAGCTATACCCCGACCATGGCGGTGGAA
ATGATTACCCTGGATAACGAAGAAACCGCGAAAAGCGTGCTGGAAGAACTGAAAGCGGAA
GGCGCGGATTTTACCGCGATTGCGAAAGAAAAAACCACCACCCCGGAAAAAAAAGTGACC
TATAAATTTGATAGCGGCGCGACCAACGTGCCGACCGATGTGGTGAAAGCGGCGAGCAGC
CTGAACGAAGGCGGCATTAGCGATGTGATTAGCGTGCTGGATCCGACCAGCTATCAGAAA
AAATTTTATATTGTGAAAGTGACCAAAAAAGCGGAAAAAAAAAAGCGATTGGCAGGAATAT
AAAAAACGCCTGAAAGCGATTATTATTGCGGAAAAAAGCAAAGATATGAACTTTCAGAAC
AAAGTGATTGCGAACGCGCTGGATAAAGCGAACGTGAAAATTAAAGATAAAGCGTTTGCG
AACATTCTGGCGCAGTATGCGAACCTGGGCCAGAAAACCAAAGCGGCGAGCGAAAGCAGC
ACCACCAGCGAAAGCAGCAAAGCGGCGGAAGAAAACCCGAGCGAAAGCGAACAGACCCAG
ACCAGCAGCGCGGAAGAACCGACCGAAACCGAAGCGCAGACCCAGGAACCGGCGGCGCAG

```
>Internalin_protein_Q99Z76_SPy_1361
ATGCACCATCATCATCATCATTACCCATACGACGTCCCAGACTACGCTTGGAGCCATCCG
CAGTTCGAGAAGGAGAACCTGTACTTCCAATCCATGGCTTGTCAATCACGAGGTAATGGT
ACATATCCCATTAAAACGAAACAATCACGTAAGGGAATGACGTCAAACAAAATTAAACCG
ATTAAAAAAAGCAAAAAGACAAACAAGACTCACAAAGGTGTGGCGGGTGTCGATTTTCCT
ACAGATGATGGGTTTATTTTAACCAAAGACTCAAAAATCTTATCAAAAACAGATCAGGGA
ATCGTTGTTGACCATGATGGTCATTCGCATTTTATTTTTTATGCCGATTTAAAGGGAAGT
CCATTTGAATACCTTATTCCAAAAGGAGCAAGTTTAGCTAAGCCAGCTGTTGCTCAGCGA
GCAGCTAGTCAAGGGACTTCTAAAGTAGCAGATCCTCATCACCATTATGAATTTAACCCA
GCGGATATTGTGGCTGAAGATGCTTTAGGCTACACGGTTCGCCACGATGATCACTTCCAT
TATATTTTGAAGTCAAGCTTATCAGGTCAGACACAGGCACAAGCTAAACAGGTTGCTACT
CGCTTGCCACAAACCAGTAGCCTTGTTTCAACAGCTACAGCTAATGGTATTCCAGGCTTG
CATTTCCCAACCTCAGATGGTTTTCAATTTAACGGTCAAGGTATTGTTGGGGTAACAAAA
GACAGTATTTTAGTGGACCACGATGGTCACTTACATCCTATTTCTTTTGCGGACCTTCGT
CAGGGTGGCTGGGCACATGTGGCAGATCAATACGATCCCGCTAAAAAAGCAGAAAAGCCA
GCAGAAACCCATCAGACACCAGAGCTATCTGAACGTGAAAAGGAATACCAAGAAAAATTA
GCTTATTTGGCAGAAAAATTGGGGATTGATCCATCAACTATTAAACGTGTGGAAACACAA
GACGGTAAACTTGGTTTGGAATACCCTCACCATGACCACGCACACGTATTGATGTTATCT
GATATTGAAATCGGAAAAGACATTCCAGATCCACATGCTATTGAGCATGCCCGTGAATTG
GAAAAACATAAGGTTGGAATGGATACCTTGCGTGCCTTAGGGTTTGATGAAGAAGTGATT
TTGGATATCGTTCGCACTCACGATGCTCCAACCCCATTCCCATCAAATGAAAAAGATCCG
AATATGATGAAAGAATGGTTAGCAACGGTTATCAAACTTGACTTGGGCAGCCGTAAAGAT
CCTTTGCAACGTAAAGGACTTTCACTGTTACCCAACTTAGAAACTTTAGGAATTGGCTTT
ACACCAATCAAAGATATCTCACCTGTTTTGCAATTTAAAAAATTGAAACAGTTGTTAATG
ACAAAAACAGGGGTGACTGATTATAGATTTTTGGATAATATGCCACAGTTAGAAGGCATT
GATATTTCACAAAACAATCTCAAAGATATTAGTTTCTTGAGCAAATATAAAAACTTAACT
CTAGTAGCGGCTGCTGATAATGGTATTGAAGATATTAGGCCGCTTGGTCAATTACCAAAT
CTCAAATTCCTCGTATTGAGTAACAATAAGATTTCTGATTTAAGCCCACTGGCATCGTTA
CATCAATTGCAAGAATTGCACATTGATAATAATCAGATTACAGATTTAAGCCCTGTTTCT
CATAAAGAATCATTGACGGTTGTTGATTTATCAAGAAATGCTGATGTTGACTTAGCAACA
CTTCAAGCACCCAAATTAGAAACGTTAATGGTCAATGATACCAAGGTTTCTCATTTGGAT
TTCTTGAAAAATAATCCTAATCTATCTAGCCTATCTATTAACCGTGCGCAATTGCAATCT
CTTGAAGGTATTGAAGCAAGTAGCGTCATTGTCAGAGTAGAAGCAGAAGGTAACCAAATT
AAATCGCTTGTGCTTAAAGACAAGCAAGGGTCACTTACTTTCTTGGATGTGACAGGCAAC
CAGTTGACTTCTCTAGAAGGTGTTAATAATTTTACAGCACTTGACATTTTAAGCGTGTCT
AAAAACCAATTAACAAATGTCAACCTATCTAAACCCAATAAGACAGTTACTAACATTGAT
ATTAGTCATAACAATATCTCATTAGCAGACCTTAAATTGAACGAGCAACATATTCCAGAA
GCCATTGCGAAAAACTTCCCAGCGGTTTACGAAGGTTCTATGGTAGGTAATGGAACAGCT
GAAGAAAAAGCAGCTATGGCTACTAAGGCGAAAGAAAGTGCTCAAGAAGCATCGGAATCA
CATGACTACAACCATAATCATACCTATGAAGATGAAGAAGGTCATGCTCACGAGCACAGA
GACAAAGATGATCACGACCATGAACATGAGGATGAAAATGAAGCTAAAGATGAGCAAAAC
CATGCTGAC

>Endonuclease/exonuclease/phosphatase_family_protein_Q9A0J7_SPy_0747
ATGCACCATCATCATCATCATTACCCATACGACGTCCCAGACTACGCTTGGAGCCATCCG
CAGTTCGAGAAGGAGAACCTGTACTTCCAATCCATGTTAAGCATTGCTTCTTACAATATT
GAAAACTTCTCAGCCAATCCTTCTTCAACTAAAGATGAGAAGGTCAAACGGATTGCCGAA
TCCTTTATTCATGATCTGAATGCTCCAGACATTATTGGATTAATTGAAGTCCAAGATAAT
AATGGGCCGACTGATGATGGGACAACGGATGCGACACAAAGCGCGCAACGCCTCATTGAT
GCTATTAAAAAACTAGGTGGCCCAACTTATCGTTATGTTGATATTGCTCCAGAAAATAAT
GTTGACGGAGGTCAACCAGGTGGTAATATTCGAACAGGATTCCTTTATCAACCAGAGCGC
GTCAGCCTTTCTGATAAGCCAAAAGGCGGTGCTCGTGATGCTCTAACTTGGGTTAATGGA
GAATTAAACCTTAGTGTTGGTCGAATTGATCCAACTAACGCCGCTTGGAAAGATGTTCGT
AAATCACTAGCAGCAGAATTTATCTTCCAAGGTCGTAAAGTCGTTGTTGTTGCAAATCAT
TTGAACTCTAAGCGTGGGGATAATGCTCTTTATGGTTGTGTGCAACCAGTCACTTTTAAA
TCTGAGCAAAGACGTCACGTCTTGGCTAATATGCTAGCACAATTTGCGAAAGAAGGGGCA
AAACACCAAGCTAATATTGTGATGCTAGGTGACTTTAATGATTTTGAATTCACAAAGACG
ATTCAATTAATCGAAGAAGGTGACATGGTTAACTTGGTGAGCCGACATGATATTTCAGAT
CGGTATTCTTATTTTCACCAAGGCAATAATCAGACCCTTGATAATATATTAGTTTCACGC
CATTTACTTGATCACTACGAATTTGACATGGTTCATGTGAATTCCCCATTTATGGAAGCT
CACGGACGCGCATCAGATCATGATCCATTGTTACTTCAATTATCA
```

>M1_protein_Q99XV0_SPy_2018
ATGAATGGTGATGGCAATCCGCGTGAAGTTATCGAAGACCTGGCAGCGAACAACCCGGCA
ATCCAAAACATCCGCCTGCGTCATGAAAACAAAGACCTGAAAGCTCGTCTGGAAAACGCT
ATGGAAGTGGCGGGCCGTGATTTTAAACGCGCCGAAGAACTGGAAAAAGCTAAACAGGCG
CTGGAAGATCAACGCAAAGACCTGGAAACCAAACTGAAAGAACTGCAGCAAGATTATGAC
CTGGCGAAAGAAAGTACGTCCTGGGATCGTCAGCGCCTGGAAAAAGAACTGGAAGAAAAG
AAAGAAGCGCTGGAACTGGCAATTGATCAGGCTTCGCGTGACTATCATCGCGCAACCGCT
TTAGAGAAAGAACTGGAAGAGAAAAAGAAAGCGCTGGAACTGGCCATCGATCAGGCAAGC
CAAGACTACAACCGTGCCAATGTTTTAGAAAAGGAACTGGAAACCATTACGCGTGAACAG
GAAATCAACCGCAATCTGCTGGGTAATGCAAAACTGGAACTGGATCAGCTGAGCTCTGAA
AAAGAACAACTGACCATTGAAAAAGCCAAACTGGAAGAAGAAAAACAGATCAGTGATGCA
TCCCGTCAATCACTGCGTCGCGATCTGGACGCTTCTCGCGAAGCGAAAAAACAGGTGGAA
AAAGATCTGGCCAACCTGACGGCAGAACTGGATAAAGTTAAAGAAGACAAACAGATCAGC
GATGCAAGCCGTCAAGGTCTGCGTCGCGATCTGGATGCTAGCCGTGAAGCGAAAAAACAG
GTCGAAAAAGACCTGGCCAATCTGACCGCAGAACTGGATAAAGTGAAAGAAGAAAAACAG
ATCTCTGATGCGAGTCGTCAAGGCCTGCGTCGCGACCTGGATGCTAGCCGCGAAGCGAAA
AAACAAGTTGAAAAAGCCCTGGAAGAAGCAAACTCCAAACTGGCGGCCCTGGAAAAACTG
AATAAAGAACTGGAAGAAAGCAAAAAACTGACCGAAAAAGAAAAAGCTGAACTGCAGGCG
AAACTGGAAGCGGAAGCCAAAGCACTGAAAGAACAGCTGGCCAAACAAGCAGAAGAACTG
GCGAAACTGCGTGCTGGCAAAGCGTCAGACTCGCAGACCCCGGATACGAAACCGGGTAAC
AAAGCAGTTCCGGGTAAGGGTCAGGCCCCGCAAGCAGGCACCAAACCGAACCAGAATAAA
GCGCCGATGAAAGAAACGAAACGCGAAAATCTGTACTTCCAAAGCTGGTCTCACCCGCAG
TTTGAAAAATACCCGTATGATGTGCCGGATTATGCCCACCACCACCACCACCAC

>Protein_G-related_alpha-2-
macroglobulin_binding_protein_GRAB_wt_Q7DAL7_SPy_1357
ATGCACCATCATCATCATCATTACCCATACGACGTCCCAGACTACGCTTGGAGCCATCCG
CAGTTCGAGAAGGAGAACCTGTACTTCCAATCCATGGACTCACCGATTGAACAACCGCGC
ATCATCCCGAACGGCGGCACCCTGACCAACCTGCTGGGCAACGCACCGGAAAAGCTGGCA
CTGCGTAACGAAGAACGCGCAATTGATGAACTGAAAAAGCAGGCCATCGAAGATAAAGAA
GCAACCACGGCTATTGAAGCGGCCAGCTCTGATGCGCTGGAAGCACTGGCTGATCAGACG
GACGCGCTGCAATCAGAAGAAGCAGCTGTGGTTAAAGCAGATAACGCAGCATCGGACGCT
CTGGAAGCACTGGCCGATCAGACCGACGCGCTGCAATCTGAAGAAGCCGAAGTCGTGCAG
AGTGATAATGCAGCTTCCGACGCGTGGGAAAAAGCGGCCACGCCGATCGCCCTGGATGTG
AAAAAGACCAAAGACACGAAGCCGGTTGTCAAAAAGGAAGAACGTCAAAACGTTAATACC

>Protein_G-related_alpha-2-macroglobulin_binding_protein_GRAB_R40K_mutant
ATGCACCATCATCATCATCATTACCCATACGACGTCCCAGACTACGCTTGGAGCCATCCG
CAGTTCGAGAAGGAGAACCTGTACTTCCAATCCATGGACTCACCGATTGAACAACCGAAA
ATCATCCCGAACGGCGGCACCCTGACCAACCTGCTGGGCAACGCACCGGAAAAGCTGGCA
CTGCGTAACGAAGAACGCGCAATTGATGAACTGAAAAAGCAGGCCATCGAAGATAAAGAA
GCAACCACGGCTATTGAAGCGGCCAGCTCTGATGCGCTGGAAGCACTGGCTGATCAGACG
GACGCGCTGCAATCAGAAGAAGCAGCTGTGGTTAAAGCAGATAACGCAGCATCGGACGCT
CTGGAAGCACTGGCCGATCAGACCGACGCGCTGCAATCTGAAGAAGCCGAAGTCGTGCAG
AGTGATAATGCAGCTTCCGACGCGTGGGAAAAAGCGGCCACGCCGATCGCCCTGGATGTG
AAAAAGACCAAAGACACGAAGCCGGTTGTCAAAAAGGAAGAACGTCAAAACGTTAATACC

>Protein_G-related_alpha-2-macroglobulin_binding_protein_GRAB_R40Q_mutant
ATGCACCATCATCATCATCATTACCCATACGACGTCCCAGACTACGCTTGGAGCCATCCG
CAGTTCGAGAAGGAGAACCTGTACTTCCAATCCATGGACTCACCGATTGAACAACCGCAG
ATCATCCCGAACGGCGGCACCCTGACCAACCTGCTGGGCAACGCACCGGAAAAGCTGGCA
CTGCGTAACGAAGAACGCGCAATTGATGAACTGAAAAAGCAGGCCATCGAAGATAAAGAA
GCAACCACGGCTATTGAAGCGGCCAGCTCTGATGCGCTGGAAGCACTGGCTGATCAGACG
GACGCGCTGCAATCAGAAGAAGCAGCTGTGGTTAAAGCAGATAACGCAGCATCGGACGCT
CTGGAAGCACTGGCCGATCAGACCGACGCGCTGCAATCTGAAGAAGCCGAAGTCGTGCAG
AGTGATAATGCAGCTTCCGACGCGTGGGAAAAAGCGGCCACGCCGATCGCCCTGGATGTG
AAAAAGACCAAAGACACGAAGCCGGTTGTCAAAAAGGAAGAACGTCAAAACGTTAATACC

>Protein_G-related_alpha-2-macroglobulin_binding_protein_GRAB_R62K_mutant
ATGCACCATCATCATCATCATTACCCATACGACGTCCCAGACTACGCTTGGAGCCATCCG
CAGTTCGAGAAGGAGAACCTGTACTTCCAATCCATGGACTCACCGATTGAACAACCGCGC
ATCATCCCGAACGGCGGCACCCTGACCAACCTGCTGGGCAACGCACCGGAAAAGCTGGCA
CTGAAAAACGAAGAACGCGCAATTGATGAACTGAAAAAGCAGGCCATCGAAGATAAAGAA
GCAACCACGGCTATTGAAGCGGCCAGCTCTGATGCGCTGGAAGCACTGGCTGATCAGACG
GACGCGCTGCAATCAGAAGAAGCAGCTGTGGTTAAAGCAGATAACGCAGCATCGGACGCT
CTGGAAGCACTGGCCGATCAGACCGACGCGCTGCAATCTGAAGAAGCCGAAGTCGTGCAG
AGTGATAATGCAGCTTCCGACGCGTGGGAAAAAGCGGCCACGCCGATCGCCCTGGATGTG
AAAAAGACCAAAGACACGAAGCCGGTTGTCAAAAAGGAAGAACGTCAAAACGTTAATACC

>Protein_G-related_alpha-2-macroglobulin_binding_protein_GRAB_R62Q_mutant
ATGCACCATCATCATCATCATTACCCATACGACGTCCCAGACTACGCTTGGAGCCATCCG
CAGTTCGAGAAGGAGAACCTGTACTTCCAATCCATGGACTCACCGATTGAACAACCGCGC
ATCATCCCGAACGGCGGCACCCTGACCAACCTGCTGGGCAACGCACCGGAAAAGCTGGCA
CTGCAAAACGAAGAACGCGCAATTGATGAACTGAAAAAGCAGGCCATCGAAGATAAAGAA
GCAACCACGGCTATTGAAGCGGCCAGCTCTGATGCGCTGGAAGCACTGGCTGATCAGACG
GACGCGCTGCAATCAGAAGAAGCAGCTGTGGTTAAAGCAGATAACGCAGCATCGGACGCT
CTGGAAGCACTGGCCGATCAGACCGACGCGCTGCAATCTGAAGAAGCCGAAGTCGTGCAG
AGTGATAATGCAGCTTCCGACGCGTGGGAAAAAGCGGCCACGCCGATCGCCCTGGATGTG
AAAAAGACCAAAGACACGAAGCCGGTTGTCAAAAAGGAAGAACGTCAAAACGTTAATACC

>Protein_G-related_alpha-2-macroglobulin_binding_protein_GRAB_R62G_mutant
ATGCACCATCATCATCATCATTACCCATACGACGTCCCAGACTACGCTTGGAGCCATCCG
CAGTTCGAGAAGGAGAACCTGTACTTCCAATCCATGGACTCACCGATTGAACAACCGCGC
ATCATCCCGAACGGCGGCACCCTGACCAACCTGCTGGGCAACGCACCGGAAAAGCTGGCA
CTGGGGAACGAAGAACGCGCAATTGATGAACTGAAAAAGCAGGCCATCGAAGATAAAGAA
GCAACCACGGCTATTGAAGCGGCCAGCTCTGATGCGCTGGAAGCACTGGCTGATCAGACG
GACGCGCTGCAATCAGAAGAAGCAGCTGTGGTTAAAGCAGATAACGCAGCATCGGACGCT
CTGGAAGCACTGGCCGATCAGACCGACGCGCTGCAATCTGAAGAAGCCGAAGTCGTGCAG
AGTGATAATGCAGCTTCCGACGCGTGGGAAAAAGCGGCCACGCCGATCGCCCTGGATGTG
AAAAAGACCAAAGACACGAAGCCGGTTGTCAAAAAGGAAGAACGTCAAAACGTTAATACC

>CovR_O87527
ATGCACCATCATCATCATCATTACCCATACGACGTCCCAGACTACGCTTGGAGCCATCCG
CAGTTCGAGAAGGAGAACCTGTACTTCCAATCCATGACGAAAAAGATTCTGATTATTGAA
GACGAAAAGAACCTGGCACGCTTTGTGTCACTGGAACTGCAACACGAAGGCTACGAAGTC
ATTGTTGAAGTCAACGGCCGTGAAGGTCTGGAAACCGCACTGGAAAAAGAATTTGATCTG
ATTCTGCTGGACCTGATGCTGCCGGAAATGGATGGCTTCGAAGTTACCCGTCGCCTGCAA
ACGGAAAAGACCACGTATATTATGATGATGACGGCGCGTGATTCTATCATGGACGTGGTT
GCAGGCCTGGATCGCGGTGCTGATGACTACATTGTTAAACCGTTTGCCATCGAAGAACTG
CTGGCGCGTATTCGCGCCATCTTCCGTCGCCAGGATATTGAAAGCGAAAAGAAAGTCCCG
TCTCAAGGCATCTATCGTGACCTGGTCCTGAACCCGCAGAATCGTAGTGTGAACCGCGGT
GATGACGAAATTTCCCTGACCAAACGCGAATACGATCTGCTGAATATCCTGATGACCAAC
ATGAATCGTGTGATGACGCGCGAAGAACTGCTGTCAAACGTCTGGAAGTATGATGAAGCC
GTGGAACCAATGTCGTGGACGTTTATATCCGTTACCTGCGCGGCAAAATTGATATCCCGG
GCAAGGAATCGTATATTCAGACGGTGCGTGGCATGGGTTACGTTATCCGCGAAAAATAAT
AAGTCGACAAGCTTGCGGCCGCACTCGAGCACCACCACCACCACCACTGAGATCCGGCTG
CTAACAAAGCCCGAAAGGAAGCTGAGTTGGCTGCTGCCACCGCTGAGCAATAACTAG

>SortaseA_Q99ZN4_SPy_1154
ATGCACCATCATCATCATCATTACCCATACGACGTCCCAGACTACGCTTGGAGCCATCCG
CAGTTCGAGAAGGAGAACCTGTACTTCCAATCCATGCGCAATAGCAATAAGTATCAGGTC
ACCAAAGTTAGCAAAAAGCAGATCAAAAAGAATAAAGAAGCAAAGAGCACGTTCGATTTC
CAGGCAGTCGAACCTGTGAGTACCGAATCCGTTCTGCAGGCTCAAATGGCAGCACAGCAA
CTGCCGGTCATTGGCGGTATTGCGATCCCGGAACTGGGCATTAACCTGCCGATCTTTAAA
GGCCTGGGTAATACCGAACTGATCTATGGCGCGGGTACGATGAAGGAAGAACAGGTGATG
GGCGGTGAAAACAATTACTCACTGGCCTCGCATCACATTTTTGGCATCACCGGTAGCTCT
CAGATGCTGTTCTCCCCGCTGGAACGTGCCCAAAACGGTATGAGCATCTACCTGACCGAT
AAGGAAAAGATCTACGAATACATCATCAAAGATGTTTTCACGGTCGCGCCGGAACGTGTG
GACGTTATTGATGACACCGCCGGCCTGAAAGAAGTGACGCTGGTTACCTGCACGGATATC

GAAGCAACCGAACGCATTATCGTTAAAGGTGAACTGAAGACGGAATATGATTTTGACAAA
GCACCGGCTGACGTCCTGAAGGCTTTCAACCATAGCTACAATCAGGTGTCTACCT

>Immunogenic_secreted_protein_Q99XU7_ISP
ATGCACCATCATCATCATCATTACCCATACGACGTCCCAGACTACGCTTGGAGCCATCCG
CAGTTCGAGAAGGAGAACCTGTACTTCCAATCCATGACGAGCCTGCGCAATAGCACCTCA
TCTACCGACCAGCCGACCACCGCCGACACCGACACCGACGACGAATCAGAAACCCCGAAA
AAAGATAAAAAGAGTAAAGAAACCGCCTCCCAGCATGATACGCAAAAAGACCACAAGCCG
AGTCATACCCACCCGACGCCGCCGTCCAACGACACCAAACAGACGGATCAAGCCAGCTCT
GAAGCAACCGATAAACCGAACAAAGATAAGAATGACACGAAGCAGCCGGATAGTTCCGAC
CAATCAACCCCGTCGCCGAAAGACCAGTCATCGCAAAAAGAAAGCCAGAACAAGGATGGC
CGTCCGACCCCGTCTCCGGATCAGCAAAAAGACCAGACCCCGGATAAAACGCCGGAAAAG
TCTGCGGATAAAACCCCGGAAAAGGGTCCGGAAAAAGCCACCGACAAGACGCCGGAACCG
AATCGCGATGCACCGAAACCGATTCAGCCGCCGCTGGCAGCAGCACCGGTGTTTATCCCG
TGGCGTGAATCAGATAAAGACCTGTCGAAACTGAAGCCGAGCTCTCGTAGTTCCCTGCTG
TCGCGTCGCTACGGTATTACGGCAGAACAGCTGGACGGCTTCCTGAACAGCCTGGGTATC
CATTACGATAAAGAACGTCTGAATGGCAAGCGCCTGCTGGAATGGGAAAAACTGACCGGT
CTGGATGTCCGTGCGATTGTGGCCATCGCAATGGCTGAATCATCGCTGGGCACCCAGGGT
GTGGCTAAAGAAAAGGGCGCGAACATGTTTGGCTATGGTGCCTTTGATTTCAATCCGAAC
AATGCAAAAAAGTACAGCGACGAAGTTGCTATTCGCCACATGGTCGAAGATACCATTATC
GCTAACAAAAATCAGACGTTTGAACGTCAAGATCTGAAGGCGAAAAAGTGGTCCCTGGGC
CAGCTGGACACCCTGATTGATGGCGGTGTGTATTTCACCGATACGAGCGGCTCTGGTCAG
CGTCGCGCAGACATTATGACGAAACTGGATCAATGGATCGATGACCATGGTTCAACCCCG
GAAATTCCGGAACACCTGAAAATCACCAGCGGCACGCAGTTTTCTGAAGTGCCGGTTGGT
TATAAACGCTCGCAGCCGCAAAACGTTCTGACCTATAAGAGCGAAACGTACTCTTTCGGC
CAGTGCACCTGGTATGCGTACAACCGTGTGAAAGAACTGGGCTATCAGGTTGACCGCTAC
ATGGGTAATGGCGGTGATTGGCAACGTAAACCGGGCTTTGTCACCACGCATAAACCGAAA
GTGGGTTATGTGGTTTCTTTCGCTCCGGGTCAGGCAGGTGCAGACGCAACCTACGGCCAC
GTTGCCGTCGTGGAACAAATCAAAGAAGATGGTAGTATCCTGATCAGTGAATCCAATGTC
ATGGGCCTGGGTACGATCAGTTATCGCACCTTCACGGCTGAACAGGCGTCCCTGCTGACC
TACGTTGTCGGTGATAAACTGCCGCGTCCG

>Immunogenic_secreted_protein_ISP-N_mutant
ATGCACCATCATCATCATCATTACCCATACGACGTCCCAGACTACGCTTGGAGCCATCCG
CAGTTCGAGAAGGAGAACCTGTACTTCCAATCCATGACGAGCCTGCGCAATAGCACCTCA
TCTACCGACCAGCCGACCACCGCCGACACCGACACCGACGACGAATCAGAAACCCCGAAA
AAAGATAAAAAGAGTAAAGAAACCGCCTCCCAGCATGATACGCAAAAAGACCACAAGCCG
AGTCATACCCACCCGACGCCGCCGTCCAACGACACCAAACAGACGGATCAAGCCAGCTCT
GAAGCAACCGATAAACCGAACAAAGATAAGAATGACACGAAGCAGCCGGATAGTTCCGAC
CAATCAACCCCGTCGCCGAAAGACCAGTCATCGCAAAAAGAAAGCCAGAACAAGGATGGC
CGTCCGACCCCGTCTCCGGATCAGCAAAAAGACCAGACCCCGGATAAAACGCCGGAAAAG
TCTGCGGATAAAACCCCGGAAAAGGGTCCGGAAAAAGCCACCGACAAGACGCCGGAACCG
AATCGCGATGCACCGAAACCGATTCAGCCGCCGCTGGCAGCAGCACCGGTGTTTATCCCG
TGGCGTGAATCAGATAAAGACCTGTCGAAACTGAAGCCGAGCTCTCGTAGTTCCGCTGCG
TATGTTCGCCATTGGACCGGCGATTCAGCCTATACGCACAATCTGCTGTCGCGTCGCTAC
GGTATTACGGCAGAACAGCTGGACGGCTTCCTGAACAGCCTGGGTATCCATTACGATAAA
GAACGTCTGAATGGCAAGCGCCTGCTGGAATGGGAAAAACTGACCGGTCT

>Superfolder_green_fluorescent_protein_sfGFP
ATGCACCATCATCATCATCATTACCCATACGACGTCCCAGACTACGCTTGGAGCCATCCG
CAGTTCGAGAAGGAGAACCTGTACTTCCAATCCATGGCGCGTAAAGGCGAAGAGCTGTTC
ACTGGTGTCGTCCCTATTCTGGTGGAACTGGATGGTGATGTCAACGGTCATAAGTTTTCC
GTGCGTGGCGAGGGTGAAGGTGACGCAACTAATGGTAAACTGACGCTGAAGTTCATCTGT
ACTACTGGTAAACTGCCGGTACCTTGGCCGACTCTGGTAACGACGCTGACTTATGGTGTT
CAGTGCTTTGCTCGTTATCCGGACCATATGAAGCAGCATGACTTCTTCAAGTCCGCCATG
CCGGAAGGCTATGTGCAGGAACGCACGATTTCCTTTAAGGATGACGGCACGTACAAAACG
CGTGCGGAAGTGAAATTTGAAGGCGATACCCTGGTAAACCGCATTGAGCTGAAAGGCATT
GACTTTAAAGAAGACGGCAATATCCTGGGCCATAAGCTGGAATACAATTTTAACAGCCAC
AATGTTTACATCACCGCCGATAAACAAAAAAATGGCATTAAAGCGAATTTTAAAATTCGC
CACAACGTGGAGGATGGCAGCGTGCAGCTGGCTGATCACTACCAGCAAAACACTCCAATC
GGTGATGGTCCTGTTCTGCTGCCAGACAATCACTATCTGAGCACGCAAAGCGTTCTGTCT

AAAGATCCGAACGAGAAACGCGATCATATGGTTCTGCTGGAGTTCGTAACCGCAGCGGGC
ATCACGCATGGTATGGATGAACTGTACAAATGA
