# Supplementary material for: A quantitative Streptococcus pyogenes–human protein–protein interaction map reveals localization of opsonizing antibodies
Source: Nat Commun. 2019 Jun 21;10:2727. doi: 10.1038/s41467-019-10583-5 (PMC6588558; doi:10.1038/s41467-019-10583-5)
Supplement: Supplementary file 10 — Reporting Summary [file 41467_2019_10583_MOESM10_ESM.pdf]

nature research

Corresponding author(s): Johan Malmström

Last updated by author(s): May 5, 2019

Reporting Summary

Nature Research wishes to improve the reproducibility of the work that we publish. This form provides structure for consistency and transparency in reporting. For further information on Nature Research policies, see Authors & Referees and the Editorial Policy Checklist.

Statistics

For all statistical analyses, confirm that the following items are present in the figure legend, table legend, main text, or Methods section.

n/a	Confirmed	
☐	☒	The exact sample size (*n*) for each experimental group/condition, given as a discrete number and unit of measurement
☐	☒	A statement on whether measurements were taken from distinct samples or whether the same sample was measured repeatedly
☐	☒	The statistical test(s) used AND whether they are one- or two-sided<br>*Only common tests should be described solely by name; describe more complex techniques in the Methods section.*
☒	☐	A description of all covariates tested
☐	☒	A description of any assumptions or corrections, such as tests of normality and adjustment for multiple comparisons
☒	☐	A full description of the statistical parameters including central tendency (e.g. means) or other basic estimates (e.g. regression coefficient) AND variation (e.g. standard deviation) or associated estimates of uncertainty (e.g. confidence intervals)
☒	☐	For null hypothesis testing, the test statistic (e.g. *F*, *t*, *r*) with confidence intervals, effect sizes, degrees of freedom and *P* value noted<br>*Give P values as exact values whenever suitable.*
☒	☐	For Bayesian analysis, information on the choice of priors and Markov chain Monte Carlo settings
☒	☐	For hierarchical and complex designs, identification of the appropriate level for tests and full reporting of outcomes
☒	☐	Estimates of effect sizes (e.g. Cohen's *d*, Pearson's *r*), indicating how they were calculated

Our web collection on statistics for biologists contains articles on many of the points above.

Software and code

Policy information about availability of computer code

Data collection	All mass spectrometric data was collected on Thermo instruments using the vendor specific published Xcalibur software.
Data analysis	All data was analyzed using published software. Mass spectrometric raw data were converted to gzipped and Numpressed mzML using the tool msconvert from the ProteoWizard, v3.0.5930 suite 59. Acquired spectra for the AP-SWATH and the initial SA-SWATH interactomes with the M1 serotype strain AP1 were analyzed using the search engine X! Tandem (2013.06.15.1-LabKey, Insilicos, ISB). Identified peptides were processed and analyzed through the Trans-Proteomic Pipeline (TPP v4.7 POLAR VORTEX rev 0, Build 201403121010) using PeptideProphet.The spectral libraries were generated from the PeptideProphet data using the Fraggle-Franklin-Tramler pipeline. The spectral library was used by DIANA to analyze the SWATH-MS data. The SRM-MS data was analyzed in Skyline. The flow-cytometry data was analyzed using FlowJo version 10.2 (Tree Star) and Prism version 7.0c (GraphPad).

For manuscripts utilizing custom algorithms or software that are central to the research but not yet described in published literature, software must be made available to editors/reviewers. We strongly encourage code deposition in a community repository (e.g. GitHub). See the Nature Research guidelines for submitting code & software for further information.

Data

Policy information about availability of data

All manuscripts must include a data availability statement. This statement should provide the following information, where applicable:

- Accession codes, unique identifiers, or web links for publicly available datasets
- A list of figures that have associated raw data
- A description of any restrictions on data availability

The mass spectrometry data have been deposited in PeptideAtlas with the identifier PASS01167.

Field-specific reporting

Please select the one below that is the best fit for your research. If you are not sure, read the appropriate sections before making your selection.

☒ Life sciences ☐ Behavioural & social sciences ☐ Ecological, evolutionary & environmental sciences

For a reference copy of the document with all sections, see nature.com/documents/nr-reporting-summary-flat.pdf

Life sciences study design

All studies must disclose on these points even when the disclosure is negative.

Sample size	No sample size calculations were performed. Each sample was prepared at least as 3 biological replicates.
Data exclusions	1% false discovery rate was applied for all mass spectrometric analyses. Proteins identified by only one peptide were excluded from the data. analysis.
Replication	Replicate experiments to verify our findings (performed on a selection of the samples, data not included in this manuscript as the purpose was only verification) were successful.
Randomization	The samples were not allocated into experimental groups, as this was not relevant for the study. Each experiment was performed as an entity and analyzed together to avoid batch differences or differences due to mass spectrometric instrument performance.
Blinding	As samples were not allocated into groups, no blinding was performed. However, the person preparing the biological samples was different from the person making the raw mass spectrometric data analysis.

Reporting for specific materials, systems and methods

We require information from authors about some types of materials, experimental systems and methods used in many studies. Here, indicate whether each material, system or method listed is relevant to your study. If you are not sure if a list item applies to your research, read the appropriate section before selecting a response.

Materials & experimental systems

n/a	Involved in the study	
☐	☒	Antibodies
☐	☒	Eukaryotic cell lines
☒	☐	Palaeontology
☒	☐	Animals and other organisms
☒	☐	Human research participants
☒	☐	Clinical data

Methods

n/a	Involved in the study	
☒	☐	ChIP-seq
☐	☒	Flow cytometry
☒	☐	MRI-based neuroimaging

Antibodies

Antibodies used

DyLight 488-conjugated affiniPure F(ab') fragment goat anti-human IgG, supplier: Jackson ImmunoResearch, catalog number: 109-486-097, LOT number: 92587.

Purified mouse anti-human fibrinogen monoclonal antibody, supplier: BD Pharmingen, catalog number: 555866, LOT number: 61181.

Goat anti-mouse IgG horseradish peroxidase (HRP) conjugate, supplier: Bio-Rad, catalog number: 172-1011, LOT number: 1721011.

Sheep anti-human serum albumin antibody, supplier: Bio-Rad, catalog number: AHP102, LOT number: 146513.

Rabbit anti-sheep IgG HRP conjugate, supplier: Bio-Rad, catalog number: 5184-2504, LOT number: 148369.

Affinity-purified protein G HRP conjugate, supplier: Bio-Rad, catalog number:170-6425, LOT number: 1706225.

Pooled human intravenous immunoglobulins (IVIG; Octagam 100 mg ml-1) was from Octapharma; catalog number: 158007, LOT number: K727C854A.

Antibodies present in pooled normal human plasma. The pooled normal human plasma was from Innovative Reasearch, LOT number: 11196.

Validation	DyLight 488-conjugated affiniPure F(ab') fragment goat anti-human IgG, purified mouse anti-human fibrinogen monoclonal antibody, goat anti-mouse IgG horseradish peroxidase (HRP) conjugate, sheep anti-human serum albumin antibody, rabbit anti-sheep IgG HRP conjugate and affinity-purified protein G HRP conjugate have been tested and validated by the suppliers. The DyLight 488-conjugated affiniPure F(ab') fragment goat anti-human IgG and the purified mouse anti-human fibrinogen monoclonal antibody have been discontinued by the manufacturer.<br><br>The antibodies enriched from pooled normal human plasma to specific pathogenic proteins or to the bacterial surface via affinity capture methods were not validated, as were not the antibodies enriched to the M1 protein or to its peptide fragments from pooled immunoglobulins in the ELISA assays.

Eukaryotic cell lines

Policy information about cell lines

Cell line source(s)	Tsuchiya, S. et al. Establishment and characterization of a human acute monocytic leukemia cell line (THP-1). Int. J. Cancer 26, 171–176 (1980).
Authentication	Purchased from ATCC
Mycoplasma contamination	THP-1 cell line tested negative for mycoplasma contamination
Commonly misidentified lines<br>(See ICLAC register)	Not to our knowlegde, not found in the ICLAC register 20190116.

Flow Cytometry

Plots

Confirm that:

☒ The axis labels state the marker and fluorochrome used (e.g. CD4-FITC).

☒ The axis scales are clearly visible. Include numbers along axes only for bottom left plot of group (a 'group' is an analysis of identical markers).

☒ All plots are contour plots with outliers or pseudocolor plots.

☒ A numerical value for number of cells or percentage (with statistics) is provided.

Methodology

Sample preparation	Cell line
Instrument	CytoFlex Beckman-Coulter
Software	CytExpert 2.1 for data collection and FlowJo version 10.2 (Tree Star) for data analysis.
Cell population abundance	15000 gated cells were analyzed per sample.
Gating strategy	Dead cells were excluded by being positive for LIVE/DEAD™ Fixable Violet Dead Cell Stain Kit, for 405 nm excitation (ThermoFisher). Live cells were gated on forward and side scatter, followed by excluding doublets by gating on FSC-H versus FSC-A. Interaction was defined by cells positive for Dylight 650 and internalization by cells positive for Dylight 650 and negative for Dylight 488. Boundaries between positive and negative has been defined using unstained and stained controls.

☒ Tick this box to confirm that a figure exemplifying the gating strategy is provided in the Supplementary Information.
